# Proteomic and metabolic disturbances in lignin-modified *Brachypodium distachyon*

**DOI:** 10.1093/plcell/koac171

**Published:** 2022-06-07

**Authors:** Jaime Barros, Him K Shrestha, Juan C Serrani-Yarce, Nancy L Engle, Paul E Abraham, Timothy J Tschaplinski, Robert L Hettich, Richard A Dixon

**Affiliations:** BioDiscovery Institute and Department of Biological Sciences, University of North Texas, Denton, Texas 76201, USA; Center for Bioenergy Innovation (CBI), Oak Ridge National Laboratory, Oak Ridge, Tennessee 37830, USA; Biosciences Division, Oak Ridge National Laboratory, Oak Ridge, Tennessee 37830, USA; Genome Science and Technology, University of Tennessee, Knoxville, Tennessee 37916, USA; BioDiscovery Institute and Department of Biological Sciences, University of North Texas, Denton, Texas 76201, USA; BioDiscovery Institute and Department of Biological Sciences, University of North Texas, Denton, Texas 76201, USA; Biosciences Division, Oak Ridge National Laboratory, Oak Ridge, Tennessee 37830, USA; Center for Bioenergy Innovation (CBI), Oak Ridge National Laboratory, Oak Ridge, Tennessee 37830, USA; Biosciences Division, Oak Ridge National Laboratory, Oak Ridge, Tennessee 37830, USA; Center for Bioenergy Innovation (CBI), Oak Ridge National Laboratory, Oak Ridge, Tennessee 37830, USA; Biosciences Division, Oak Ridge National Laboratory, Oak Ridge, Tennessee 37830, USA; Center for Bioenergy Innovation (CBI), Oak Ridge National Laboratory, Oak Ridge, Tennessee 37830, USA; Biosciences Division, Oak Ridge National Laboratory, Oak Ridge, Tennessee 37830, USA; BioDiscovery Institute and Department of Biological Sciences, University of North Texas, Denton, Texas 76201, USA; Center for Bioenergy Innovation (CBI), Oak Ridge National Laboratory, Oak Ridge, Tennessee 37830, USA; BioEnergy Science Center (BESC), Oak Ridge National Laboratory, Oak Ridge, Tennessee 37830, USA

## Abstract

Lignin biosynthesis begins with the deamination of phenylalanine and tyrosine (Tyr) as a key branch point between primary and secondary metabolism in land plants. Here, we used a systems biology approach to investigate the global metabolic responses to lignin pathway perturbations in the model grass *Brachypodium distachyon*. We identified the lignin biosynthetic protein families and found that ammonia-lyases (ALs) are among the most abundant proteins in lignifying tissues in grasses. Integrated metabolomic and proteomic data support a link between lignin biosynthesis and primary metabolism mediated by the ammonia released from ALs that is recycled for the synthesis of amino acids via glutamine. RNA interference knockdown of lignin genes confirmed that the route of the canonical pathway using shikimate ester intermediates is not essential for lignin formation in Brachypodium, and there is an alternative pathway from Tyr via sinapic acid for the synthesis of syringyl lignin involving yet uncharacterized enzymatic steps. Our findings support a model in which plant ALs play a central role in coordinating the allocation of carbon for lignin synthesis and the nitrogen available for plant growth. Collectively, these data also emphasize the value of integrative multiomic analyses to advance our understanding of plant metabolism.

## Teaser

Ammonia-lyases play a key role in coordinating the allocation of carbon for lignin synthesis and nitrogen availability for plant growth.

## Introduction

Lignin is a carbon-rich phenolic polymer found in the cell walls of land plants that plays a central role in plant structure, nutrient transport, and adaptation to biotic and abiotic stresses (Whetten and Sederoff, 1995; Boerjan et al., 2003; Bonawitz and Chapple, 2010). Lignin biosynthesis accounts for up to 40% of the dry weight of land plants, representing a major route capturing atmospheric carbon fixed through photosynthesis (Hermann, 1995; Vogel, 2008; Bugg et al., 2011). This natural abundance and chemical properties of lignin make it an attractive source for biological and catalytic conversion into renewable fuels, chemicals, and bioproducts, as well as a target for carbon storage and sequestration (Ragauskas et al., 2014; Bar-On et al., 2018). However, our current understanding of the biosynthesis of lignin is still incomplete, especially in the Poaceae family.

Lignin biosynthesis is developmentally regulated, as well as induced by biotic and abiotic stresses and limitations in nutrients, such as iron, phosphorus, or nitrogen (Dixon and Paiva, 1995). The balance between nitrogen and carbon can affect phenylpropanoid metabolism. For example, supplying nitrogen inhibits the phenylpropanoid pathway (Fritz et al., 2006; Zhao et al., 2021), whereas limiting nitrogen or supplying sucrose as an external carbon source activates phenylpropanoid metabolism (Nose et al., 1995; Nemie-Feyissa et al., 2014). There is still an incomplete understanding of the molecular mechanisms underlying these effects. Some of the theoretical bases include the “carbon-nitrogen,” or “carbon-nutrient,” “growth-differentiation,” and “growth-defense” balance hypotheses (Bryant et al., 1983; Herms and Mattson, 1992; Stamp, 2003; Huot et al., 2014; Monson et al., 2021), postulating that primary and secondary metabolism compete for photoassimilates, and there is a trade-off in the allocation of carbon. These concepts provide a possible explanation of the phenomenon but lack molecular understanding, and are still, therefore, widely debated (Donaldson et al., 2006; Fritz et al., 2006).

The aromatic amino acids phenylalanine (Phe) and tyrosine (Tyr) are the end products of the shikimate pathway and the precursors of all phenylpropanoids. The shikimate pathway primarily occurs in plastids (Maeda and Dudareva, 2012) and involves the transamination of prephenate by prephenate:glutamate aminotransferase (PAT) to form arogenate using glutamate as an amino donor (Supplemental Figure S1). Arogenate can be converted to Phe via arogenate dehydratase (ADT) or Tyr via arogenate dehydrogenase (ADH). The first step of the phenylpropanoid pathway in plants is catalyzed by Phe/Tyr ammonia (NH_3_)-lyases (P/TALs), which deaminate Phe to cinnamate and/or Tyr to *p*-coumarate, yielding equimolar amounts of NH_3_. In the aqueous environment of the cytoplasm, NH_3_ gas is spontaneously converted into more stable free ammonium (${\text{NH}}_{4}^{+}$) ions. The amount of cytosolic ${\text{NH}}_{4}^{+}$ generated from the P/TAL reactions is assumed to be high considering the large flux of carbon channeled to the phenylpropanoid pathway. It has been estimated that the amount of ${\text{NH}}_{4}^{+}$ recycled from the synthesis of phenylpropanoids in terrestrial plants is two-thirds of the total primary nitrogen assimilation, including both atmospheric fixation of dinitrogen and uptake/reduction of nitrite and nitrate (Raven et al., 1992). To avoid nitrogen deficiency, or ${\text{NH}}_{4}^{+}$ toxicity, the ${\text{NH}}_{4}^{+}$ generated during active lignin biosynthesis is efficiently and primarily recycled into glutamine and glutamate via the glutamine synthetase (GS) and glutamate synthase (GOGAT) cycle (Razal et al., 1996; van Heerden et al., 1996; Singh et al.,1998; Pascual et al., 2016). In this P/TAL-derived nitrogen cycle, GS transfers ${\text{NH}}_{4}^{+}$ to form glutamine from glutamate using ATP, and GOGAT transfers the amino group of glutamine to α-ketoglutarate in the presence of NADH, producing two molecules of glutamate. Finally, glutamate donates the amino group to PAT for Phe and Tyr regeneration, closing the cycle, and the free hydroxycinnamic acids cinnamate and *p*-coumarate serve as initial precursors for the synthesis of flavonoids, benzoates, coumarins, suberins, lignans, and lignins (Supplemental Figure S1).

Lignin is produced via a series of tightly controlled enzymatic reactions leading to the formation of three major monomers or monolignols: *p*-coumaryl, coniferyl, and sinapyl alcohols. Monolignols are synthesized in the cytosol, transported to the apoplast, and incorporated into the growing lignin polymer as *p*-hydroxyphenyl (H), guaiacyl (G), and syringyl (S) subunits. Over the last 40 years, both biochemical and genetic evidence has led to the identification of many genes of the monolignol biosynthetic pathway. Early models from the 1980s, mainly based on the activities of purified enzymes from crude protein extracts, suggested that lignin formation starts with the direct hydroxylation and methylation of *p*-coumarate to ferulate and sinapate, followed by the conversion of these acids to monolignols via 4-coumarate: CoA ligase (4CL), cinnamoyl CoA reductase (CCR) and cinnamyl alcohol dehydrogenase (CAD) (Higuchi, 1985). This pathway via free phenolic acids was challenged in the 1990s by the discovery of two methylation pathways in zinnia (*Zinnia elegans*) (Ye et al, 1994; Ye and Varner, 1995) and tobacco (Zhong et al., 1998), mediated by caffeate 3-*O*-methyltransferase (COMT) and caffeoyl CoA 3-*O*-methyltransferase (CCoAOMT), and by genetic and biochemical evidence in Arabidopsis (*Arabidopsis thaliana*) of a ferulate 5-hydroxylase (F5H) acting at the coniferaldehyde and coniferyl alcohol levels (Chapple et al., 1992; Meyer et al., 1996).

Subsequent biochemical and genetic evidence indicating that COMT acts more efficiently at the level of the aldehyde or alcohol than the acid was then reported in Arabidopsis, alfalfa (*Medicago sativa*), and aspen (*Populus tremuloides*) (Humphreys et al., 1999; Li et al., 2000; Parvathi et al., 2001). In the early 2000s, two parallel studies using functional genomics and mutant screening approaches in Arabidopsis reported the characterization of a 4-coumaroyl shikimate 3′-hydroxylase (C3′H) using shikimate and quinate esters of *p*-coumarate as substrates (Schoch et al., 2001; Franke et al., 2002a, 2002b). These findings led to a search for genes involved in the production of shikimate/quinate esters, and hydroxycinnamoyl CoA: shikimate/quinate hydroxycinnamoyltransferase (HCT) was first characterized functionally in tobacco and Arabidopsis (Hoffmann et al., 2004) and later in alfalfa (Shadle et al., 2007), pine (*Pinus radiata*) (Wagner et al., 2007), and black poplar (*Populus nigra*) (Vanholme et al., 2013a). Subsequently, genetic analysis led to the discovery of caffeoyl shikimate esterase (CSE) mediating the hydrolysis of caffeoyl shikimate to caffeate in Arabidopsis (Vanholme et al., 2013b) and *Medicago truncatula* (Ha et al., 2016). These studies provide strong evidence for the role of shikimate esters and aldehydes/alcohols as major intermediates in the phenylpropanoid pathway, primarily in dicot species. However, it was not until recently that grasses were shown to exhibit weaker phenotypes than dicots following the downregulation of some genes of the canonical pathway (Shen et al., 2013; Nelson et al., 2017; Serrani-Yarce et al., 2021) and to have the unique ability to synthesize roughly half of their lignins from Tyr via Phe/Tyr ammonia lyase (PTAL) (Barros et al., 2016). Grasses also possess a direct route to caffeate via 4-coumarate 3-hydroxylase (C3H) (Barros et al., 2019). Additionally, genetic studies in rice suggested that parallel pathways to lignin formation independent of C3′H and F5H act at the shikimate ester and aldehyde/alcohol levels, respectively (Takeda et al., 2018, 2019). Thus, the literature suggests that the conventional pathway involving free acids should be reconsidered and further explored in grasses.

In this study, to further investigate lignin metabolism in grasses, we undertook a combined genetic, proteomic, and ^13^C-isotopic labeling approach in the model grass *Brachypodium distachyon*. We first developed five RNA interference (RNAi)-mediated gene-silenced lines targeting lignin pathway genes *PAL2*, *PTAL1*, *C4H1*, *C3′H1*, and *HCT1*, displaying a variety of lignin and growth phenotypes. We then identified the complete set of monolignol pathway proteins and showed by proteomic analysis that the key enzymes in the formation of free hydroxycinnamic acids PTAL, C3H/APX, and COMT were among the most abundant proteins in mature stems. Overall proteomic and metabolomic changes observed in the lignin knockdown (KD) lines support metabolic crosstalk between the phenylpropanoid and other pathways, particularly those related to nitrogen metabolism and ${\text{NH}}_{4}^{+}$ recycling. Our ^13^C-labeling data reveal a degree of metabolic separation of the carbon flux from Phe and Tyr into different lignin subunits and between flavonoid classes and suggest yet uncharacterized enzymatic steps in the lignin pathway. Altogether, our data provide a deeper understanding of lignin metabolism in the economically important Poaceae family of grasses.

## Results

### Lignin biosynthesis pathway gene KD lines exhibit altered lignin deposition, shifts in lignin monomer subunit composition, and a range of growth phenotypes

We first applied an RNAi approach for silencing five early lignin pathway genes: *PAL2*, *PTAL1*, *C4H1*, *C3′H1*, and *HCT1*. The target RNAi fragments to silence these genes are shown in Supplemental Figure S2. Multiple R0 generation transgenic lines were obtained for each construct, and lines combining the best transcript level downregulation with minimal off-target effects on related lignin pathway gene transcripts were selected as parental lines to develop R1 generation plants. For example, the optimal *BdPAL2i* line should have close to wild-type (WT) *PTAL1* expression, and vice versa. This led to the selection of *BdPALi-77*, with *PAL2* downregulated and *PTAL1* transcript levels most similar to the control, and *BdPTAL1i-27*, with similar expression levels of *PAL2* compared with the control and downregulation of *BdPTAL1* transcripts (Supplemental Figure S3, A and B). All the Brachypodium RNAi lines selected in the R1 generation showed >75% reduction in target gene transcript levels relative to the WT controls, except for the *BdPAL2i* lines, which displayed a ∼40% reduction in *PAL2* transcript levels (Supplemental Figure S3C).

**Figure 1:**
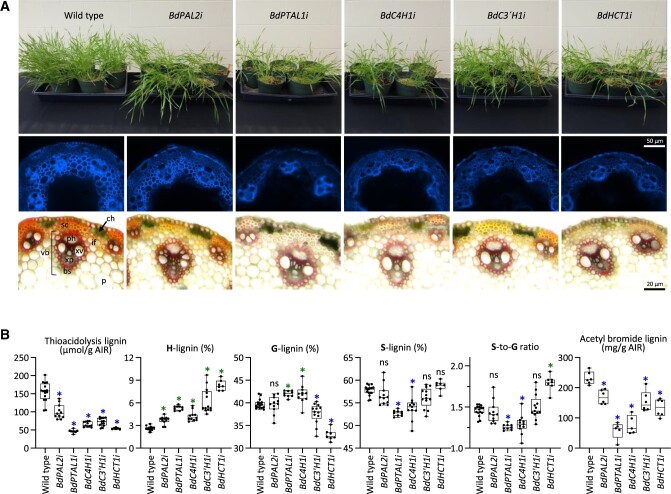
Plant growth and lignin phenotypes of Brachypodium RNAi lines. A, Visual phenotypes of lignin pathway gene KD lines and WT control Brachypodium plants grown in pots in the greenhouse for 30 days after germination. UV-microscopy and phloroglucinol-HCl staining of transverse mature stem sections of the genotypes studied. B, Relative yields of the lignin-derived thioacidolysis monomers recovered from H, G, or S β-O-4-linked lignin units (in micromoles per gram of AIR, relative (%) monolignol composition, and S/G ratios in the same stem internodes. ch, chlorenchyma; sc, sclerenchyma; if, interfascicular fibers; ph, phloem; xv, xylem vessels; bs, bundle sheath; xp, xylem parenchyma, vb, vascular bundle; p, parenchyma. Lignin monomers H, G, and S. Box plots indicate the median (center lines), interquartile range (hinges), and whiskers represents min and max values. Green and blue asterisks indicate significant upregulated and downregulated lignin content/composition, respectively (*P* < 0.05, one-way ANOVA with post hoc Dunnett’s test). ns, denotes no significant difference from control plants. Plants were harvested at 30 days after germination when the first inflorescence was emerging, equivalent to the booting stage in the BBCH-scale for cereals (Lancashire et al., 1991). The more lignified lower internodes were used for microscopy, whereas the upper internodes from each plant were combined and used for lignin, metabolomic, and proteomic analyses.

Seeds from the R1 generation were harvested and all five KD lines were planted along with the WT controls and grown under greenhouse conditions (Figure 1A). For each genotype, ∼25 seeds were grown in five different pots. All transgenic plants exhibited a lodging phenotype and more but shorter internodes (Supplemental Figure S4A). With the exception of *BdHCT1i*, all RNAi lines showed a significant reduction in above-ground biomass (>50% for *BdPTAL1i* and *BdC4H1i* lines), which was consistent among different aerial plant parts (Supplemental Figure S4B). Analyses of transverse sections of the stems of all RNAi lines revealed reduced lignin autofluorescence and less intense phloroglucinol-HCl staining in vascular bundles and sclerenchyma and interfascicular fibers compared with control plants (Figure 1A). The lignin content measured either by the acetyl bromide method or as the relative yields of the lignin-derived thioacidolysis monomers showed highly significant reductions (>50%) in all RNAi lines, which also showed increased H-lignin subunit levels, with the largest increases found for the *BdHCT1i* (220%), *BdC3′H1i* (140%), and *BdPTAL1i* (104%) lines compared with WT (Figure 1B). Downregulation of *PAL2* had only modest effects on lignin composition (i.e. slightly increased levels of H-units). The proportion of G-lignin was significantly reduced in the *BdHCT1i* lines but increased in the *BdPTAL1i* and *BdC4H1i* lines compared with the WT. Accordingly, the S:G ratios were increased in the *BdHCT1i* lines and reduced in *BdPTAL1i*, and *BdC4H1i*. The *BdHCT1i* lines showed strong reductions in lignin content with no impact on biomass yield. We recently reported an increased saccharification efficiency in these lines (Serrani-Yarce et al., 2021), making *HCT1i* a promising target for the development of improved lignocellulosic biomass from grasses. Taken together, these data suggest that whereas *PTAL1* is preferentially involved in the biosynthesis of S-units (Barros et al., 2016), *C3′H1* and *HCT1* participate more actively in the biosynthesis of G-lignin.

### Targeted metabolomics reveals *p*-coumarate as the most abundant phenylpropanoid intermediate and efficient blockage of the shikimate shunt in *BdC3′H1* lines

**Figure 2:**
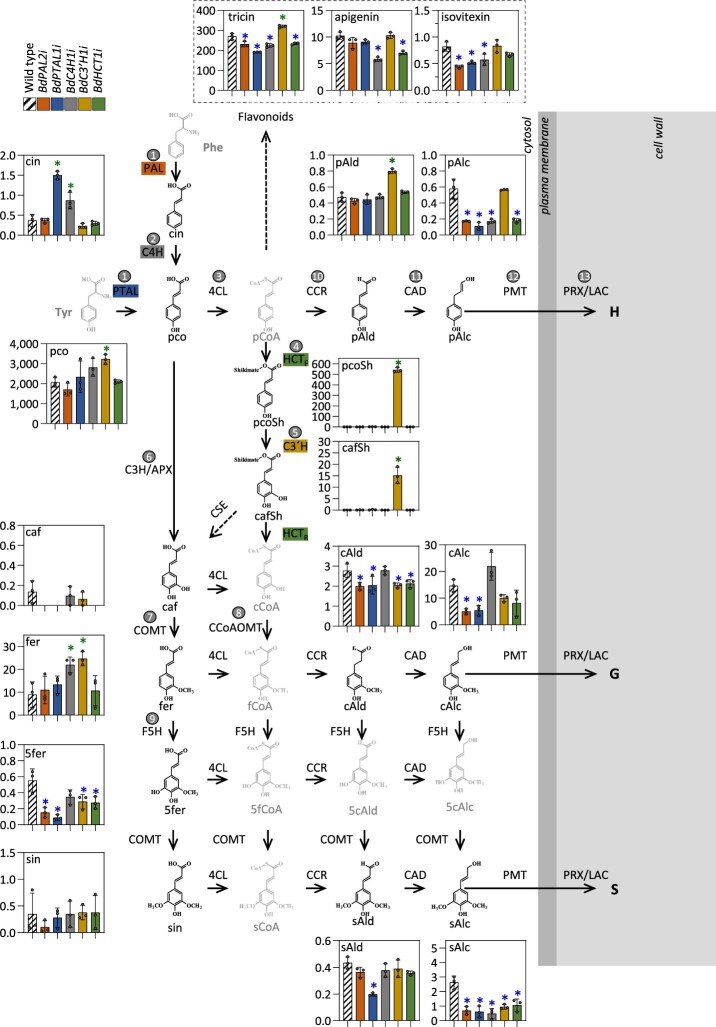
Concentrations of soluble phenylpropanoids in mature stem tissues of Brachypodium RNAi lines. Concentrations of soluble metabolites in micromoles per gram dry weight in mature stem internodes harvested from plants at 30 days after germination and measured by LC–MS/MS. Metabolites shown in light gray were not quantified. The five genes targeted for RNA silencing and characterized in this study are highlighted with different colors. Gray circles depict the enzymatic steps presented in Table 1. The CSE gene, which is absent in many grasses, is depicted with a discontinuous arrow. cin, cinnamate; pco, p-coumarate; caf, caffeate; fer, ferulate; sin, sinapate; pCoA, *p*-coumaroyl CoA; pcoSh, *p*-coumaroyl shikimate; cafSh, caffeoyl shikimate; cCoA, caffeoyl CoA; fCoA, feruloyl CoA; 5fCoA, 5-hydroxy-feruloyl CoA; sCoA, sinapoyl CoA; pAld, p-coumaraldehyde; cAld, coniferaldehyde; 5cAld, 5-hydroxy-coniferaldehyde; sAld, sinapaldehyde; pAlc, p-coumaryl alcohol; cAlc, coniferyl alcohol; 5cAlc, 5-hydroxy-coniferyl alcohol; sAlc, sinapyl alcohol. Lignin monomers H, G, and S. Error bars indicate ± sd (*n* = 3). Green and blue asterisks indicate significant upregulated and downregulated phenylpropanoid metabolite content, respectively (*P* < 0.05, one-way ANOVA with post-hoc Dunnett’s test).

To gain further insight into the regulation of monolignol biosynthesis in the RNAi lines, we carried out a targeted metabolomics approach focused on a subset of phenylpropanoid pathway metabolites using liquid chromatography coupled mass spectrometry (LC–MS/MS) (Figure 2). Overall, the levels of most soluble phenolics in WT stems at 30 days after germination were ˂15 nmol/g dry weight, whereas the concentration of *p*-coumarate reached ∼2,000 nmol/g dry weight. The methoxylated flavone tricin was the second most abundant soluble phenylpropanoid of those targeted, at 270 nmol/g dry weight.

The RNAi lines with the largest growth defects and largest lignin reductions (*BdPTAL1i* and *BdC4H1i*) showed increased levels of cinnamate, the substrate of C4H and first intermediate in the lignin biosynthetic pathway (Figure 2). As expected based on the role of C3′H1 in catalyzing the 3′-hydroxylation of *p*-coumaroyl shikimate to caffeoyl shikimate, the *BdC3*′*H1i* lines over-accumulated *p*-coumaroyl shikimate, with up to a 360-fold (540 nmol/g dry weight) increase compared with the controls. However, the levels of caffeoyl shikimate detected in the *BdC3′H1* KD lines (∼3% of *p*-coumaroyl shikimate quantity) demonstrate the presence some residual 3′-hydroxylase activity in these lines. The *BdC3′H1i* lines also over-accumulated the most abundant free phenolic acids (*p*-coumarate and ferulate), the flavonoid tricin, and the precursor of the H-units of lignin *p*-coumaraldehyde. All RNAi lines showed levels of monolignol precursors in good agreement with the decreased lignin content. Moreover, the levels of both *p*-coumaraldehyde and *p*-coumaryl alcohol in the *BdC3′H1i* lines paralleled the increased H-unit levels of these lines, whereas the increased levels of coniferaldehyde and coniferyl alcohol in the *BdC4H1i* lines correspond to their relatively higher proportion of G-lignin. Similarly, the low levels of 5-hydroxy-ferulate and sinapaldehyde in the *BdPTAL1i* lines correspond to their lower proportions of S-units. Furthermore, the reduced concentration of 5-hydroxy-ferulate in all KD lines suggests that 5-hydroxy-ferulate is a lignin pathway intermediate (Figure 2).

### PTAL1, COMT1, and C3H/APX1 are the most abundant lignin biosynthesis pathway proteins in mature Brachypodium stems

We applied an integrated genomic and proteomic approach to identify the enzymes involved in the chemical reactions and pathways resulting in the formation of lignin expressed in Brachypodium stems. First, we obtained the sequences of all members of the Arabidopsis lignin biosynthetic gene family from TAIR and performed a BLAST search for Brachypodium homologs based on their DNA sequence similarity in Phytozome. Candidates with similarities ˂60%, or 50% for cytochrome P450s, were excluded from analysis. Second, we qualitatively rated the in silico gene expression intensity observed in the first stem internode of 35-days-old Brachypodium plants using microarray data from the Bio-Analytic Resource (BAR) for plant biology (Waese and Provart, 2017). Finally, we annotated the abundance of these proteins in WT plants as raw peak areas and as relative percentages to the most abundant lignin biosynthetic enzyme PTAL1. Following these criteria, we identified a total of 82 potential candidate lignin biosynthetic proteins, including 18 homologs of the Arabidopsis laccases (LACs) and peroxidases (PRXs) involved in lignification (Supplemental Data Set S1). Among these candidates, we selected 48 proteins with detectable levels in the stems (Supplemental Data Set S2). By removing the less abundant members of each family, we narrowed down the number of core lignin enzymes in the stems of Brachypodium to 23 proteins, as shown in Table 1. This approach allowed us to identify the closest Brachypodium homologs to annotated lignin biosynthetic enzymes in Arabidopsis. However, some of these genes still remain to be functionally characterized.

**Table 1:** Families of lignin biosynthetic genes/proteins in the model grass Brachypodium

At Gene	At ID	Bd Gene	Bd ID	Siml. %	UniProt ID	RNAi target	In silico expression	Protein level
Step 1: PTAL:								
At *PAL1*	At2g37040	Bd PTAL1*	Bradi3g49250	77	I1IBR5	Y	High	100
		Bd PAL2*	Bradi3g49260	79	I1IBR6	Y	High	17.2 ± 0.7
Step 2: Cinnamate 4-hydroxylase (C4H, CYP73A5, REF3):								
At *C4H*	At2g30490	Bd C4H1*	Bradi2g53470	86	I1HKP5	Y	Low	2.7 ± 0.7
		Bd *C4H2*	Bradi2g31510	78	I1HSV5	N	Moderate	2.1 ± 0.3
Step 3: 4-Coumarate:CoA ligase (4CL)								
At *4CL1*	At1g51680	Bd *4CL2*	Bradi3g05750	75	I1HXV5	N	High	5.1 ± 0.3
		Bd *4CL3*	Bradi1g31320	66	I1GVQ1	N	High	2.6 ± 0.1
Step 4: Hydroxycinnamoyl CoA:shikimate/quinate HCT:								
*At HCT*	At5g48930	Bd HCT1*	Bradi5g14720	77	I1IZB2	Y	High	0.3 ± 0.03
		Bd *HCT2*	Bradi3g48530	75	I1IBH8	N	Moderate	0.6 ± 0.06
Step 5: 4-Coumaroyl shikimate/quinate 3′-hydroxylase (C3′H, CYP98A3, REF8):								
At *C3′H1*	At2g40890	Bd C3′H1*	Bradi2g21300	76	I1HI50	Y	High	1.5 ± 0.1
Step 6: C3H /ascorbate PRX (C3H/APX):								
At *C3H/APX1*	At1g07890	Bd *C3H/APX1*	Bradi1g65820	79	I1H6P1	N	n.a.	21.2 ± 2.4
Step 7: COMT:								
At *COMT*	At5g54160	Bd *COMT1*	Bradi3g16530	68	I1I1F5	N	High	46.3 ± 2.5
Step 8: CCoAOMT:								
At *CCoAOMT1*	At4g34050	Bd *CCoAOMT2*	Bradi3g39420	67	I1I899	N	n.a.	7.3 ± 1.2
		Bd *CCoAOMT4*	Bradi3g39380	64	A0A0Q3FGP6	N	High	1.5 ± 0.1
Step 9: F5H/coniferaldehyde 5-hydroxylase (F5H, CYP84A1, FAH):								
At *F5H1*(*CYP84A1*)	At4g36220	Bd *F5H1*	Bradi3g30590	68	A0A0Q3FH21	N	High	3.2 ± 0.4
Step 10: CCR:								
At *CCR1*	At1g15950	Bd *CCR1*	Bradi3g36887	74	I1I7E4	N	Moderate	0.8 ± 0.05
Step 11: CAD:								
At *CAD4* (*CAD-C*)	At3g19450	Bd *CAD1*	Bradi3g06480	82	I1HY48	N	High	8.0 ± 1.0
At *CAD5* (*CAD-D*)	At4g34230	Bd *CAD2*	Bradi4g29780	64	I1IPY8	N	Low	2.3 ± 0.3
Step 12: PMT:								
–	–	Bd *PMT1*	Bradi2g36910	100	I1HM65	N	High	1.6 ± 0.2
Step 13: Monolignol-oxidizing LAC:								
At *LAC7*	At3g09220	Bd *LAC7*	Bradi2g55050	61	I1HTF1	N	Moderate	0.02 ± 0.003
At *LAC12*	At5g21100	Bd *LAC12*	Bradi1g37620	67	I1GXW0	N	Moderate	0.2 ± 0.05
Step 13: Monolignol-oxidizing PRX:								
At *PRX39*	At4g11290	Bd *PRX39*	Bradi2g11295	52	I1HER4	N	n.a.	0.06 ± 0.02
At *PRX64*	At5g42180	Bd *PRX64*	Bradi3g60880	62	A0A2K2D641	N	Moderate	0.3 ± 0.07
At *PRX72*	At5g66390	Bd *PRX72*	Bradi2g40590	74	I1HNE3	N	Moderate	1.1 ± 0.2

Candidates selected based on their similarity to Arabidopsis homologs and in silico transcript expression and protein abundance in mature stems. The percent of similarity (Siml. %) was obtained from Phytozome (https://phytozome.jgi.doe.gov). In silico transcript expression levels were qualitatively rated (high-moderate-low) based on the signal observed in microarray data from 35-day-old first stem Brachypodium internodes (http://bar.utoronto.ca/). Protein intensity levels are reported as percentages of the most abundant lignin protein (BdPTAL1). The protein IDs of all family members and actual protein intensity peak areas of the four independent replicates are provided in Supplemental Data Set S2. The target genes for RNA interference evaluated in this study are highlighted in bold with asterisks (see Figure 1 to pinpoint each enzymatic step).

Our results show that PTAL1 is the most abundant lignin biosynthesis protein, followed by the proteins involved in the two subsequent steps: the 4-*O*-methylation of caffeate to ferulate (COMT1; 46% of PTAL1) and the 3-hydroxylation of *p*-coumarate to caffeate (C3H/APXs; 13%–21% of PTAL1) (Supplemental Figure S5A). All monofunctional PALs showed abundance levels ∼10% that of PTAL1. Overall, the high levels of proteins involved in the formation of free hydroxycinnamic acids (PTAL, C3H/APX, and COMT) contrast with the low abundance of the enzymes of the parallel shikimate shunt (HCT, C3′H, and CSE). Our approach revealed three C3H/APX1 protein homologs, two isoforms I1H6P1 and I1H6P2 encoded by Bradi1g65820, and I1GQU0 encoded by Bradi1g16510 (Supplemental Figure S5A and Supplemental Data Set S1). I1H6P1 and I1H6P2 differ in the final amino acids at their C-termini. The distinctive C-terminal residues of I1H6P2 (VCLLLLSTKPGCRDM) are not part of the PRX domain and have no predicted GO terms or domains associated with it in InterPro. 1GQU0 showed high similarity to I1H6P1 (81%) and the strongest in silico expression in mature roots and stems (Supplemental Data Set S1). Proteins with moderate abundance included most cytochrome P450s, CADs, *p*-coumaroyl-CoA: monolignol transferase (PMT), other OMTs, and the flavonoid pathway enzymes chalcone synthase (CHS; TT4), chalcone isomerase (CHI; TT5), and flavonoid 3′hydroxylase (F3′H; TT7). All CCRs, and the monolignol oxidizing enzymes LACs and PRXs, showed abundances ˂1% that of PTAL1 in WT stems.

To better evaluate the abundances of LACs and PRXs, we explored the recently published cell-wall proteome data from Brachypodium WT stem internodes (Douche et al., 2021). We found that only 9 out of the 25 LACs and 72 out of the 186 PRXs annotated in Phytozome were cell-wall related enzymes (Supplemental Figure S5B). Among these, 3 LACs and 13 PRXs displayed a Protein Abundance Index (Rappsilber et al., 2002) >2. In the entire dataset of 2,116 proteins, the most abundant LAC (Bradi3g59210) ranked #76, whereas the most abundant PRX (Bradi5g27220) ranked #15. LAC 8 (Bradi2g23370, LAC8), recently characterized by Le Bris et al. (2019), ranked #873, but LAC 5 (Bradi1g66720, LAC5) was not identified in the cell-wall proteome. The weak lignin phenotype of the *lac5 lac8* mutants in metaxylem cells (Le Bris et al., 2019) suggests that in vascular tissues, other monolignol oxidizing enzymes may compensate for the loss-of-function of *LAC8* and *LAC5*. The cell-wall proteome data narrow down the number of candidates to be tested in future studies.

PTAL1 and COMT1 were among the top 60 most abundant enzymes in our dataset that included more than 11,000 plant proteins, and PTAL1 was the fourth most abundant protein after the small and large RuBisCO subunits and Elongation Factor 1 (Supplemental Figure S5C and Supplemental Data Set S3). Furthermore, all three C3H/APX homologs were within the 180 most abundant proteins in Brachypodium stems. To determine whether the unexpectedly high abundance of certain monolignol pathway enzymes was a general phenomenon across plant species, we mined previously published proteomic results from various tissues of rice (*Oryza sativa*) (Wang et al., 2014; Wu et al., 2016; Lin et al., 2017), maize (*Zea mays*) (Marcon et al., 2015; Feng et al., 2017), switchgrass (*Panicum virgatum*) (Ye et al., 2016; Qiao et al., 2021), and Arabidopsis (Li et al., 2016; Miller et al., 2017) and found that PTAL, C3H/APX, and COMT ranked among the most abundant proteins in vascular tissues (roots and stems), irrespective of the plant species tested and methods used for sample preparation and protein identification/quantification (Supplemental Figure S6 and Supplemental Data Set S4). It is noteworthy that only the root proteome dataset in Arabidopsis showed all three shikimate shunt enzymes (HCT, C3′H, and CSE) among the top 20% most abundant proteins.

### RNAi lines show co-downregulated levels of other enzymes involved in monolignol biosynthesis

**Figure 3:**
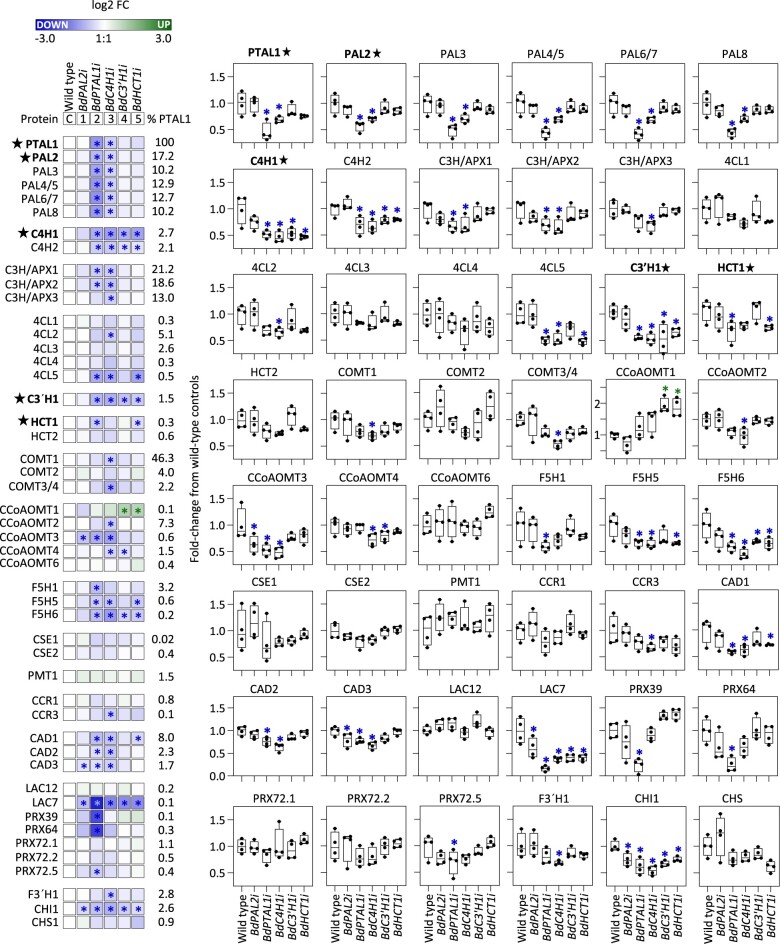
Levels of lignin biosynthetic protein families in mature stem tissues of Brachypodium RNAi lines. Protein levels were determined in mature stem internodes harvested from plants at 30 days after germination and measured by LC–MS/MS. Heatmap displays log2 FCs in abundance of lignin protein homologs in RNAi-mediated lignin gene silenced lines. The target genes for RNA interference are shown in bold and highlighted with a black star. Each square represents the mean of four biological replicates. Right column shows relative intensity levels as proportion of the most abundant lignin protein (PTAL1). Independent replicated FC values compared with WT controls are shown in the box plots on the right. Raw protein abundances values are provided in Supplemental Data Set S2. CHS, CHI, and flavonoid 3′hydroxylase (F3′H) were included in the study as representative flavonoid pathway proteins. Other proteins are abbreviated as described in Table 1. Blue and green asterisks indicate significantly downregulated and upregulated proteins, respectively (*P* *<* 0.05, one-way ANOVA with post hoc Dunnett’s test). Box plots indicate the median (center lines), interquartile range (hinges) and whiskers represents min and max values.

To better understand the basis for the lignin phenotypes observed, we compared the levels of the lignin biosynthetic enzymes in mature stem tissues from the RNAi lines and WT plants (Figure 3). With the exception of PAL2 protein in *BdPAL2i* lines, all other targets for RNAi were also silenced at the protein level (Figure 3). For example, the abundance of PTAL1 was 55% lower in the *BdPTAL1i* lines than the control plants. Similarly, the levels of C4H1, C3*′*H1, and HCT1 in the *BdC4H1i*, *Bd C3′H1i*, and *BdHCT1i* lines were 54%, 47%, and 25% lower than in WT plants, respectively. The severely reduced lignin deposition and impaired growth phenotypes observed in the *BdPTAL1i* and *BdC4H1i* lines were associated with decreased levels of several lignin biosynthetic proteins, including (among others) all ammonia-lyases (ALs), both C4Hs, several C3H/APXs, C3*′*H1, several F5Hs, and all CADs (Figure 3). LAC7 was the only lignin protein downregulated in all transgenic lines, whereas several monolignol P450 monooxygenases (C4Hs, C3*′*H1, and F5H6) were repressed in all KD lines except in the *BdPAL2i* lines. Interestingly, CCoAOMT1 was the only lignin biosynthetic enzyme with increased levels, up to two-fold in the *BdC3′H1i* and *BdHCT1i* lines. Moreover, the reduced levels of the flavonoid biosynthetic enzyme CHI, encoded by a single gene in Brachypodium (*CHI1*), suggest co-downregulation of flavonoid and lignin biosynthesis in all lignin gene KD lines.

### RNAi lines display broad changes in the levels of primary metabolites and their associated enzymes

**Figure 4:**
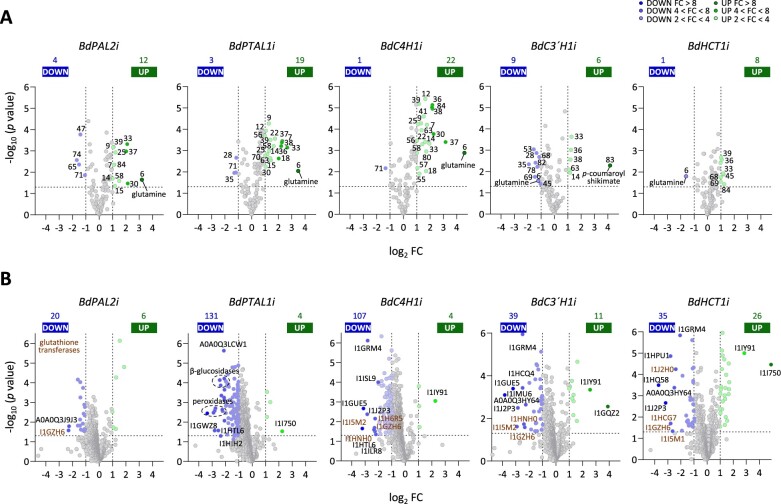
Integrated metabolomic and proteomic analysis in mature stem internodes of *Brachypodium* RNAi lines. Volcano plots showing differentially expressed metabolites (A) and proteins (B) in stem internodes of transgenic low lignin RNAi lines versus WT plants. Metabolites and proteins were plotted based on their log2 FC in abundance (RNAi line/WT) and their −log10 *P*-value in significance based on four biological replicates. Dashed horizontal lines indicate *P* = 0.05, vertical dashed lines indicate FCs >2. Metabolites highlighted in (A): glutamine (6); asparagine (7); aspartate (9); alanine (12); phytol (14); N-metabolite (15); N-metabolite (18); threonate (22); shikimate (25); glycerol-1/3-phosphate (28); 5-hydroxytryptamine (30); glutamate (33); G-lignan (35); N-metabolite (36); N-metabolite (37); N-metabolite (38); quinate (39); N-metabolite (41); SA 2-*O*-glucoside (45); leucine (47); lysine (53); adenosine (55); Phe (56); monopalmitin (57); 3-hydroxyproline (58); fucosterol (63); citraconate (65); SA (68); gentisic acid-5-O-glucoside (69); phosphoethanolamine (70); glucuronic acid conjugate (71); 1,2,4-benzenetriol (74); ascorbate conjugate (78); azelaic acid (80); G-lignan (82); *p*-coumaroyl shikimate (83); guanine (84). Proteins identified in (B): *BdPAL2i* (L-gulonolactone oxidase, A0A0Q3J9J3; glutathione transferase, I1GZH6); *BdPTAL1i* (chlorophyll a-b binding protein, A0A0Q3LCW1; alpha-amylase, I1GWZ8; chitinase, I1HTL6; aldo-keto reductase, I1HIH2; 3-methylcrotonyl-CoA carboxylase, I1I750); *BdC4H1i* (magnesium chelatase, I1GRM4; FAD diphosphatase, I1ISL9; beta-ureidopropionase, I1GUE5; asparaginase, I1J2P3; glutathione transferase, I1I5M2; glutathione transferase, I1H6R5; glutathione transferase, I1GZH6; glutathione transferase, I1HNH0; chitinase, I1HTL6; GPI-anchor transamidase, I1ILR8; gamma-glutamylcyclotransferase, I1IY91); *BdC3*′*H1i* (magnesium chelatase, I1GRM4; uncharacterized protein, I1HCQ4; beta-ureidopropionase, I1GUE5; Calmodulin kinase, I1IMU6; uncharacterized protein, A0A0Q3HY64; asparaginase, I1J2P3; glutathione transferase, I1HNH0; glutathione transferase, I1I5M2; glutathione transferase, I1GZH6; gamma-glutamylcyclotransferase, I1IY91; hexosyltransferase, I1GQZ2); *BdHCT1i* (magnesium chelatase, I1GRM4; 5′-deoxynucleotidase, I1HPU1; 3-oxoacyl-ACP synthase, I1J2H0; ribokinase, I1HQ58; uncharacterized protein, A0A0Q3HY64; asparaginase, I1J2P3; glutathione transferase, I1HCG7; glutathione transferase, I1GZH6; glutathione transferase, I1I5M1; gamma-glutamylcyclotransferase, I1IY91; 3-methylcrotonyl-CoA carboxylase, I1I750). Full metabolic shifts by RNAi line are provided in Supplemental Figures S8–S12.

We next carried out untargeted metabolomics coupled with proteome data analyses to identify broader molecular changes in response to lignin modification in the RNAi lines, generating volcano plots to display differentially regulated metabolites and proteins (Figure 4) and plotting these data on kyoto encyclopedia of genes and genomes (KEGG) metabolic maps for a visual comparison of the impact of lignin pathway perturbations on key areas of plant metabolism (Supplemental Figures S7–S12). All primary and secondary metabolites measured in this study are plotted to the reference pathways in KEGG in Supplemental Figure S7, including six unidentified nitrogen-containing metabolites (15, 18, 36, 37, 38, and 41), one glucuronic acid conjugate (71), one ascorbate conjugate (78), and three lignans (28, 35, and 82) designated by their retention time and key *m*/*z* ratios, as described in the “Materials and methods.” The bar plots and heatmap depicting the relative levels of the primary metabolites in the five RNAi lines are shown in Supplemental Figures S13 and S14.

#### BdPAL2i lines

PALs are soluble cytoplasmic proteins and the largest family of cytosolic lignin biosynthetic enzymes. Brachypodium has eight members in this family, including *PTAL1*, described below. We targeted *PAL2* (Bradi3G49260) for KD because it has the highest transcript levels in the stems among all monofunctional PALs (Supplemental Figure S15; Cass et al., 2015). Glutamine (6) was the metabolite with the most highly increased levels (greater than eight-fold) in the *BdPAL2i* lines, followed by glutamate (33), an N-containing metabolite (37) and serotonin (30) (greater than four-fold), whereas leucine (47), citraconate (65), a glucuronic acid conjugate (71), and 1,2,4-benzenetriol (74) were the metabolites with the most highly reduced levels (greater than two-fold) (Figure 4A). The *BdPAL2i* lines also over-accumulated the aromatic amino acids Tyr (48) and Phe (56), and the upstream aromatic amino acid precursors shikimate (25) and quinate (39) (Supplemental Figures S13 and S14). These transgenic lines showed the most symmetrical volcano plots for protein levels, with only two oxidoreductases with a role in ascorbate metabolism, a cytosolic glutathione-dependent dehydroascorbate reductase (I1GZH6), and gulonolactone oxidase (A0A0Q3J9J3) highly downregulated (greater than four-fold) (Figure 4B). Impacts at the metabolite and related protein levels were found for succinate (40, up) with succinate dehydrogenase (I1H3W3, up); adenosine (55, up) with adenosine kinase (I1IZP9, down); Phe (56, up) with Phe AL (I1IBR6, down); 3-hydroxyproline (58, up) with prolyl 4-hydroxylase (I1I533, down); and all three monolignols (J, K, and L, down) and a G-lignan (82, down) with one lignin PRX (I1GUH6, down) (Supplemental Figure S8).

#### BdPTAL1i lines

PTALs deaminate both Phe and Tyr to form cinnamate and *p*-coumarate, respectively. We previously reported that PTAL1 has higher catalytic efficiency toward Tyr (Barros et al., 2016). Together with the *BdC4H1i* lines described below, the *BdPTAL1i* lines displayed the most asymmetrical volcano plots for both protein and metabolite levels and the most severe reductions in lignin levels and growth (Figures 1 and 4). Similar to the *BdPAL2i* lines, glutamine (6) was the metabolite with the most highly increased level (greater than eight-fold), followed by glutamate (33), four N-containing metabolites (18, 36, 37, and 38), and asparagine (7), whereas an S-lignan glycoside (28), a G-lignan (35), leucine (47), and a glucuronic acid conjugate (71) were among the metabolites with most highly reduced levels (greater than two-fold) in these transgenic lines (Figure 4A). The *BdPTAL1i* lines also accumulated Tyr (48), Phe (56), shikimate (25), and quinate (39) (Supplemental Figures S13 and S14). The most highly upregulated protein in these lines was a methylcrotonyl-CoA carboxylase (I1I750) involved in leucine degradation, whereas over 20 lignin PRXs and 10 β-glucosidases were among the most repressed proteins (greater than four-fold) (Figure 4B). Several hexosyltransferases with a role in pectin biosynthesis (A0A0Q3H0X2, I1H4M2, I1J2B7, and I1I2T0) were also repressed up to 2.5-fold in the *BdPTAL1i* lines (Supplemental Figure S9).

Metabolic perturbations at both the metabolite and protein levels were found for sucrose (1, down) with sucrose synthase (I1H5N7, up); glutamine (6, up) with glutamine-dependent carbamyl phosphate synthetase (I1HXQ9, down); asparagine (7, up) with asparaginase (I1J2P3, down); alanine (12, up) with alanine transaminase (I1HIG7, down); fumarate (27, up) with succinate dehydrogenase (I1H3W3, up); glutamate (33, up) with glutamate-glyoxylate aminotransferase (I1HIG7, down); glycolate (49, down) with glyoxylate reductase (I1IVV9, down); and adenine (79, up) with adenine phosphoribosyl transferase (I1IGY4, down). In some cases, the changes in levels of linked metabolites and proteins were not in the direction predicted (see “Discussion”), such as example 5-oxoproline (4, up) with gamma-glutamylcyclotransferase (I1IY91, down); aspartate (9, up) with cyanoalanine nitrilase (I1IBW1, down); linoleate (31, up) with phospholipase A2 (I1GP43, down); and α-ketoglutarate (42, up) with two isocitrate dehydrogenases (I1IB67, I1IYV8, down). In the phenylpropanoid pathway, the reduced concentrations of all three monolignols (J, K, L) and the identified lignans (28, 35, 82) in the *BdPTAL1i* lines was accompanied by downregulation of one CAD (I1I1X8) and 23 PRXs (Supplemental Figure S9). Among these PRXs, 16 were also found in the cell-wall proteome (Douche et al., 2021), providing indirect evidence for their role in facilitating lignin polymerization.

#### BdC4H1i lines

C4H is a membrane-bound cytochrome P450 monooxygenase that catalyzes the formation of *p*-coumarate from cinnamate. There are two members of this gene family in Brachypodium. We chose *C4H1* as a target for RNAi because of its higher transcript levels in mature stem internodes (Supplemental Figure S15). Similar to the *BdPAL2i* and *BdPTAL1i* lines, the *BdC4H1i* lines showed increased levels of Tyr (48), Phe (56), shikimate (25), and quinate (39), and glutamine (6) was the metabolite with the most highly increased level (>16-fold) (Figure 4A andSupplemental Figures S13 and S14). These lines also showed increased levels of three N-containing metabolites (36,37,38), serotonin (30), and guanine (84) (greater than four-fold), whereas only a glucuronic acid conjugate (71) showed highly reduced levels compared with control plants (greater than two-fold) (Figure 4A). Gamma-glutamylcyclotransferase (I1IY91, up) and several glutathione transferases (A0A2K2DD42, I1I5M2, I1GZH6, I1HNH0, and I1H6R5, down) involved in glutathione metabolism were among the proteins with the most highly altered levels (greater than four-fold; Figure 4B).

Impacts at both metabolite and related protein levels were observed for 5-oxoproline (4, up) with gamma-glutamylcyclotransferase (I1IY91, up); asparagine (7, up) with asparaginase (I1J2P3, down); glycine (26, up) with glycine cleavage system H protein (I1HXP5, down); glutamate (33, up), and α-ketoglutarate (42, down) with glutamate dehydrogenase (I1J084, down); glycolate (49, down) with two glyoxylate reductases (A0A0Q3MVN2 and A0A0Q3E1W6, down); and an ascorbate conjugate (78, down) with cytosolic dehydroascorbate reductase (I1GZH6, down) (Supplemental Figure S10). Unexpected changes occurred for aspartate (9, up) with asparaginase (I1J2P3, down); threonine (23, up) with threonine synthase (I1HQL5, down); and Tyr (48, up) and Phe (56, up) with ADH (I1GWX1, down) and dehydratases (I1I5J4 and I1ISZ5, down). Several metabolites and a large number of enzymes involved in glycerolipid metabolism were differentially regulated in the *BdC4H1i* lines, such as glycerol 3-phosphate (29, up) and glycerol (34, up) with two glycerol-3-phosphate acyltransferases (I1GTT3 and I1HWB0, down) and glycerol kinase (I1I299, down); and ethanolamine (8, up) and phosphoethanolamine (70) with two ethanolamine kinases (I1HCQ4 and A0A2K2CRK8, down). The reduced concentrations of sinapyl alcohol (L), *p*-coumaryl alcohol (J), and G-lignan (28) were associated with the strong downregulation of one CAD (I1I1X8) and five PRXs (I1H555, I1HF21, I1GUH6, I1HD49, and I1H7Q3). Moreover, four cytoplasmic ferredoxins (I1H2D5, I1GL62, I1GPH9, and I1I0B2) involved in photosynthetic electron transport and fructose-bisphosphatases (I1HJ10, I1J0H4, I1GYQ5, and I1I3V8) involved in gluconeogenesis were also downregulated in these transgenic lines. Another particular feature of the *BdC4H1i* lines was the increased levels of purines (55, 72, 79, and 84) and most phytosterols (13, 53, 50, and 59) identified (Supplemental Figure S10).

#### BdC3′H1i lines

The cytochrome P450 C3′H catalyzes the formation of caffeoyl shikimate from *p*-coumaroyl shikimate. Brachypodium possesses only one member of this family that is highly expressed in mature stems (Supplemental Figure S15). The *BdC3′H1i* lines showed fewer upregulated metabolites than the other RNAi lines, but *p*-coumaroyl shikimate, the substrate of C3′H, showed a 16-fold increase in levels compared with the controls (Figure 4A). The levels of phytol (14), two N-containing metabolites (36 and 38), glutamate (33) and fucosterol (63) (greater than two-fold) were increased, whereas an ascorbate conjugate (78), two G-lignans (82 and 35) one S-lignan glycoside (28) and lysine (53) showed reduced levels in these transgenic lines. In contrast to the *PAL2*, *PTAL1*, and *C4H1* KD lines, the *BdC3′H1i* lines displayed reduced levels of glutamine (6) and similar levels of Tyr (48), and Phe (56) as the control plants (Supplemental Figures S13 and S14). The impacts at the linked metabolite and protein levels were similar to those of the *BdC4H1i* lines, with ascorbate conjugate (78, down) and a cytosolic dehydroascorbate reductase (I1GZH6, down); glutamate (33, up) and α-ketoglutarate (42, down) with glutamate dehydrogenase (I1J084, down); and glycerol 3-phosphate (29, up) with two glycerol-3-phosphate acyltransferases (I1GTT3, down) (Supplemental Figure S11). Among the monolignol intermediates, the *BdC3′H1i* lines had elevated levels of *p*-coumarate, *p*-coumaraldehyde, and *p*-coumaroyl shikimate, supporting the role of C3′H in the 3-hydroxylation of *p*-coumaroyl moieties, as well as lower levels of sinapyl alcohol (L) and all the lignans identified (28, 35, and 82). A defining trait of the *BdC3′H1i* lines was the reduced levels of salicylic acid (SA; 68), SA 2-*O* glucoside (45) and gentisic acid 5-*O*-glucoside (69) (Supplemental Figure S11).

#### BdHCT1i lines

HCT is a BAHD acyltransferase that catalyzes the conversion of *p*-coumaroyl-CoA to *p*-coumaroyl shikimate. HCT can operate in the reverse direction from caffeoyl shikimate to caffeoyl CoA (HCT_R_ in Figure 2), although this enzymatic step is inefficient both in vitro and in vivo (Vanholme et al., 2013a, 2013b; Serrani-Yarce et al., 2021). Of the two members of the HCT family in Brachypodium, we targeted *HCT1* for KD because of its higher expression levels in mature stems (Supplemental Figure S15). These transgenic lines were particularly interesting because they exhibited severe reductions in lignin contents with no impact on plant growth, the most symmetrical metabolite volcano plots, and the largest number of highly upregulated proteins (greater than two-fold) (Figures 1 and 4). Moreover, glutamine (6) was the only metabolite with highly reduced levels (greater than two-fold), whereas SA (68), SA 2-*O* glucoside (45), and gentistic acid 5-*O*-glucoside (69) exhibited increased levels in the *BdHCT1i* lines (Figure 4A). These lines also showed increased levels of guanine (84), glutamate (33), N-containing metabolite (36), and quinate (33) (greater than two-fold). *BdHCT1i* lines had similar levels of Tyr (48) and Phe (56) as the control plants but higher levels of quinate and shikimate (Supplemental Figures S13 and S14). The most highly upregulated proteins were gamma-glutamylcyclotransferase (I1IY91), with a role in glutathione homeostasis, and methylcrotonoyl-CoA carboxylase (I1I750), which is involved in leucine degradation, whereas four glutathione transferases (I1I5M1, I1GZH6, I1HCG7, and I1HNH0) were among the most downregulated proteins (greater than four-fold) (Figure 4B). A magnesium chelatase (I1GRM4) with a role in photosynthesis was highly repressed in all three C4H1, C3′H1, and HCT1 KD lines, whereas the abundance of a phosphopantetheine adenylyltransferase (A0A0Q3HY64) involved in CoA biosynthesis was reduced in both the *BdHCT1i* and *BdC4H1i* lines (greater than four-fold) (Figure 4B). Change in the levels of metabolites and related proteins occurred for 5-oxoproline (4, down) with gamma-glutamylcyclotransferase (I1IY91, up); fumarate (27, up) and succinate (40, up) with succinate dehydrogenase/fumarate reductase (I1H3W3, up); and phosphoethanolamine (70, up) with two ethanolamine kinases (I1HCQ4 and A0A2K2CRK8, down). In the lignin pathway, the reduced abundance of one PRX (A0A0Q3KBL7), one CAD (I1I1X8), and one 4CL (I1I2A9) were observed simultaneously with decreased concentrations of ferulate (E), *p*-coumaryl (J), and sinapyl (L) alcohols (Supplemental Figure S12).

### Stable isotope labeling supports a separation of pathways from Phe and Tyr into different phenylpropanoids

**Figure 5:**
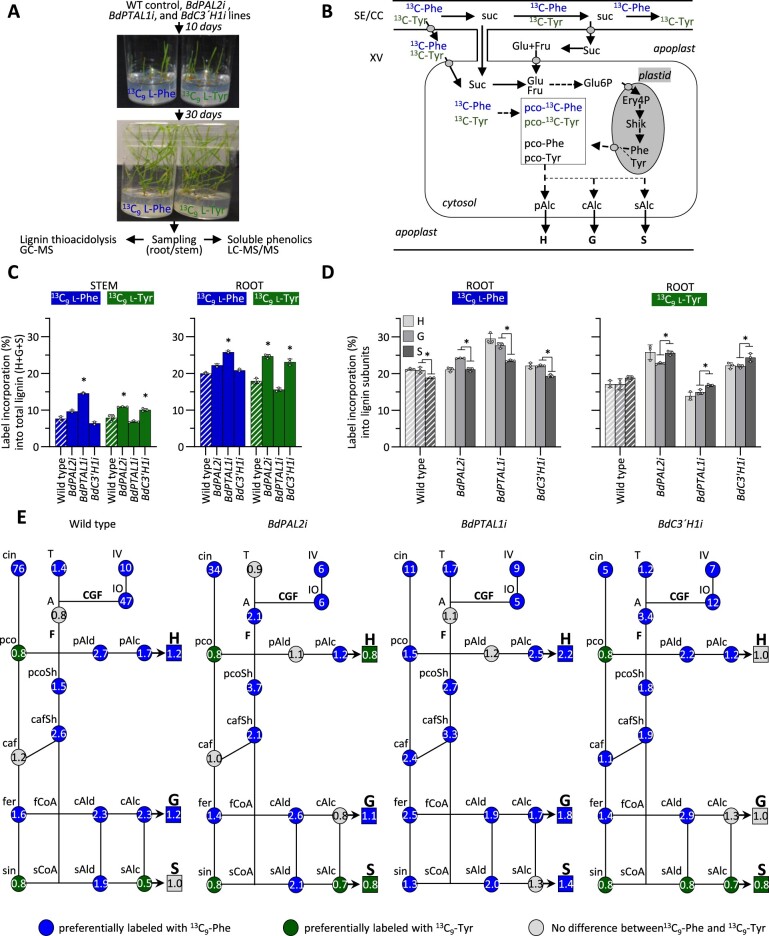
Stable isotope labeling experiments. A, Layout of the in vivo stable isotope labeling experiment. B, Conceptualization of the labeled precursor feeding experiments showing the different pools of labeled and unlabeled *p*-coumarate (pco). C, Proportion of labeled precursors incorporated into total lignin in stem and root tissues of 30 days-old in vitro grown plants. D, Proportion of labeled precursors incorporated into each individual monolignol (H-, G-, and S-units) in root tissues of 30-days-old in vitro grown plants. E, Simplified lignin pathways depicting the ratio between incorporation of ^13^C_9_-Phe and ^13^C_9_-Tyr for each pathway intermediate and lignin subunit in roots of Brachypodium RNAi lines. Dashed bars are control plants, error bars indicate ±sd (*n* = 3). Asterisks indicate significant differences: *P* < 0.0001, one-way ANOVA with post-hoc Dunnett’s test in (C), and *P* <0.05, two-sided unpaired t test in (D). In (E), blue and green colors indicate metabolites that preferentially incorporate ^13^C_9_-Phe or ^13^C_9_-Tyr, respectively (*P* < 0.05, two-sided unpaired *t* test).

To further study the operation of the phenylpropanoid pathway following downregulation of individual monolignol biosynthesis enzymes, we measured the incorporation of ^13^C-labeled Tyr and Phe into lignin and its precursors in WT and *BdPALi*, *BdPTAL1i*, and *BdC3′H1i* lines. The design of the in vitro isotopic labeling experiment is provided in Figure 5A. Using this method, labeled lignin precursors in the culture medium are readily available in the cytosol of lignifying cells. Additionally, unlabeled pools of Phe and Tyr are endogenously synthesized in the plastids. Both labeled and unlabeled pools of *p*-coumarate derived from Phe (via PAL and C4H) and Tyr (by PTAL) can be used for the synthesis of monolignols in the cytosol, which are trafficked to the apoplast and polymerized into lignin (Figure 5B). We measured the incorporation of both ^13^C-labeled substrates into the H, G, and S monolignols and several monolignol pathway intermediates, including free phenolic acids, shikimate esters, aldehydes, and alcohols, as well as some flavonoids. We found good incorporation of both ^13^C_9_-labeled Phe (^13^C_9_-Phe) and Tyr (^13^C_9_-Tyr) into the three major monolignols (H, G, and S) in the extracted lignin fraction (Figure 5, C and D). The patterns of label incorporation into the lignin polymer across the transgenic lines were similar for both root and stem tissues. However, as a result of their more direct contact with the labeled precursors, roots showed overall higher label incorporation levels than stems (Figure 5C). Notable in both root and stem tissues were the increased incorporation of ^13^C_9_-Phe into all monomers in the *BdPTAL1i* lines and the higher incorporation of ^13^C_9_-Tyr into the lignins of the *BdPAL2i* and *BdC3′H1i* lines (Figure 5C). Overall, the different genotypes showed higher incorporation of ^13^C_9_-Phe into G- than into S-units, whereas ^13^C_9_-Tyr showed moderately better incorporation into S- than into G-units. ^13^C_9_-Phe and ^13^C_9_-Tyr were both incorporated very well into H-lignin (>15% of total H-units pool) (Figure 5D). Brachypodium grown in vitro deposited a higher proportion of H-lignin (>13%) than plants grown in pots in the greenhouse or growth chamber (<6%) (Supplemental Figure S16).

We next examined the distribution of label in monolignol pathway intermediates and biosynthetically related flavonoids among the RNAi lines (Supplemental Figure S17). We chose root tissues for this analysis because they showed a larger proportion of isotopically labeled lignin and yielded significantly more material (fresh weight) than stem tissues. The levels of label incorporated into soluble free phenolic acids (*p*-coumarate, ferulate, and sinapate) resembled the pattern observed in lignin, with increased incorporation from ^13^C_9_-Phe in the *BdPTAL1i* lines and relatively better incorporation from ^13^C_9_-Tyr in the *BdPAL2i* and *BdC3′H1i* lines. Somewhat higher incorporation of ^13^C_9_-Tyr in the *BdPAL2i* and *BdC3′H1i* lines was also observed for the three monolignols: *p*-coumaryl, coniferyl, and sinapyl alcohols. Cinnamate and both *p*-coumaroyl and caffeoyl shikimate esters showed overall less label incorporation than other pathway intermediates. Most striking was the observation that ^13^C_9_-Tyr incorporated well into the flavones apigenin and tricin, but very poorly into two flavone C-glycosides characteristic of the grass family: isovitexin (apigenin 6-C-glucoside) and isoorientin (luteolin 6-C-glucoside). Among all the soluble phenylpropanoid intermediates, only *p*-coumarate, sinapate, and sinapyl alcohol showed preferential incorporation of ^13^C_9_-Tyr in all transgenic lines except *BdPTAL1i*; this finding is consistent with a pathway proceeding from Tyr to sinapyl alcohol via soluble free phenolic acids (Supplemental Figure S17).

We then calculated the ^13^C_9_-Phe/^13^C_9_-Tyr labeling ratios of the pathway intermediates and lignin subunits (Figure 5E). Cinnamate, isoorientin, and isovitexin almost exclusively incorporated ^13^C_9_-Phe, whereas other phenylpropanoids and all the lignin subunits incorporated both ^13^C_9_-Phe and ^13^C_9_-Tyr. All transgenic lines showed preferential incorporation of ^13^C_9_-Phe into *p*-coumaryl alcohol, the two shikimate esters, ferulate, and coniferaldehyde. *PAL2* downregulation led to marked shifts in the ^13^C_9_-Phe/^13^C_9_-Tyr labeling ratios of apigenin (from 0.8 to 2.1) and coniferyl alcohol (from 2.3 to 0.8). The most pronounced changes in ^13^C_9_-Phe/^13^C_9_-Tyr labeling ratios among the RNAi lines were observed for the *BdPTAL1i* lines, with reduced incorporation ratios of ^13^C_9_-Tyr into *p*-coumarate (from 0.8 to 1.5), caffeate (from 1.2 to 2.4), ferulate (from 1.6 to 2.5), sinapate (from 0.8 to 1.3), and particularly sinapyl alcohol (from 0.5 to 1.3). In contrast, the *BdC3′H1i* lines displayed extensive incorporation of ^13^C_9_-Phe into apigenin, and preferential incorporation of ^13^C_9_-Tyr into *p*-coumarate, sinapate, sinapaldehyde, sinapyl alcohol, and S-lignin (Figure 5E). We found significant differences in the ratios of labeled sinapaldehyde to its product sinapyl alcohol formed by the CAD reaction, especially in *BdPAL2i* (2.1 versus 0.7) and WT (1.9 versus 0.5) plants. Compared with the WT controls, the Phe-labeled pools of sinapaldehyde were increased in the *BdPAL2i* lines and reduced in the *BdC3′Hi* lines, whereas the Tyr-labeled pools of sinapaldehyde were only increased in the *BdC3′Hi* lines (Supplemental Figure S17). These observations suggest that at least a portion of the Phe-derived pools of sinapate could derive from the corresponding aldehydes via aldehyde dehydrogenase (ALDH) (Nair et al., 2004), as discussed below.

## Discussion

### Implications of the high abundance of PTAL1

Our interest in lignin biosynthesis in commelinid monocots has been driven by the characteristic properties of grass lignins (Hatfield et al., 2009), their unique ability to use Tyr as a substrate (Rosler et al., 1997; Barros et al., 2016), and their phylogenetically widespread lack of orthologous genes potentially encoding CSE enzymes (Vanholme et al., 2013a, 2013b; Ha et al., 2016; Barros et al., 2019). In this study, we performed an integrated metabolomic, proteomic, and stable isotopic labeling analysis of a set of KD lines for several enzymes in the monolignol pathway using the model grass Brachypodium. We used this approach to analyze the labeling of intermediates in phenylpropanoid metabolism and gain a deeper understanding of how grasses adapt this metabolism to alterations in the levels of lignin pathway enzymes. Our data support the notion that parallel pathways to lignins in grass cells exhibit cross-talk with other metabolic pathways.

The extraordinarily high abundance of the three soluble proteins that provide a direct route from Phe and Tyr to ferulate (PTAL, C3H/APX, and COMT), together with the observation that PTAL and C3H enzymatic activities copurify and coelute in the same FPLC fractions (Barros et al., 2019), suggests that a cytosolic route to monolignols via free phenolic acids may exist as a complex that engenders high substrate turnover, NH_3_ release, and protein–protein interactions. The Km value of recombinant Brachypodium C3H for *p*-coumarate (∼600 µM) is higher than that of the competing reaction catalyzed by 4CLs in rice (100–300 µM) and sorghum (*Sorghum bicolor*) (4–15 µM) (Saballos et al., 2012; Sun et al., 2013; Barros et al., 2019). However, the specific activity of C3H toward *p*-coumarate in maize root extracts is higher than that of 4CL (76 versus 43 pkat/mg protein) (Barros et al., 2019), suggesting that the high soluble protein concentration and a potential protein complex might help divert flux toward the acids pathway. Flexible regulation of very high abundance proteins such as PTAL, COMT, and C3H/APX requires rapid post-translational modifications, and ALs, *O*-methyltransferases, and ascorbate PRXs were found to be phosphorylated (Bolwell, 1992; Allwood et al., 1999; Wang et al., 2015; Hu et al., 2021), highlighting the need to further investigate the kinases that may regulate the early steps of the lignin pathway in cereals, as recently reviewed by Sulis and Wang (2020).

### Cross-talk between primary and secondary metabolism

Several studies have shown that lignin-modified plants have altered expression of several genes in the same or different pathways (Rohde et al., 2004; Sibout et al., 2005; Vanholme et al., 2012; Scully et al., 2018). Our data show co-downregulation of proteins within the lignin biosynthetic pathway (Figure 3), and crosstalk between lignin biosynthesis and other pathways, particularly in the RNAi lines with the most severe reductions in lignin levels and growth inhibition (Supplemental Figures S8–S12). Using the PAL mutants in Arabidopsis, Rohde et al. (2004) reported that the hyperaccumulation of Phe, required as a substrate for lignification, might cause adjustments in the overall amino acid pools and consequent changes in other metabolic pathways. Consistent with this observation, the *BdPAL2i*, *BdPTAL1i*, and *BdC4H1i* lines accumulated Phe, Tyr, Asp, Asn, Ser, Ala, and Thr and displayed highly increased levels of metabolites involved in nitrogen metabolism, such as glutamine, glutamate, and their cyclic lactam 5-oxoproline (Supplemental Figure S13).

**Figure 6:**
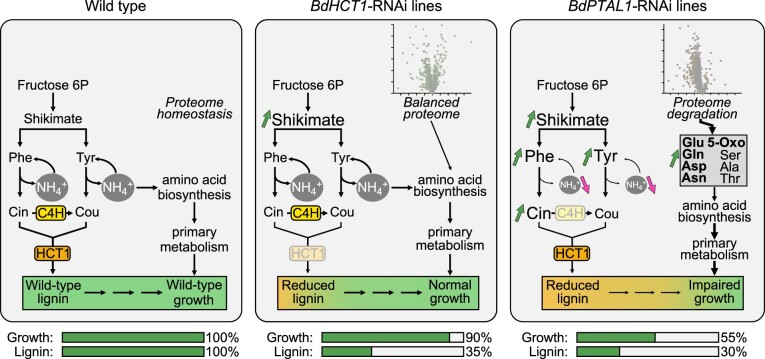
Model of major impacts on lignin, metabolomic, and proteomic profiles in Brachypodium RNAi lines with contrasting growth phenotypes. Fructose 6P, fructose 6-phosphate; Cou, *p*-coumarate; Cin, cinnamate; Glu, glutamate; Gln, glutamine; Asp, aspartate; Asn, asparagine; 5-Oxo, 5-oxoproline; Ser, serine; Ala, alanine; Thr, threonine; ${\text{NH}}_{4}^{+}$, recycled ${\text{NH}}_{4}^{+}$ ions (see Supplemental Figure S1); C4H1, cinnamate 4-hydroxylase 1; HCT1, hydroxycinnamoyl CoA:shikimate hydroxycinnamoyltransferase 1.

We hypothesize that blocking both ALs may lead to a marked reduction in the availability of ${\text{NH}}_{4}^{+}$ recycled for the synthesis of amino acids that could be replaced by amino acids derived from protein breakdown (Figure 6). Our data show that the *BdPTAL1i* and *BdC4H1i* lines had strong co-downregulation of other lignin proteins, including all ALs, as well as increased levels of Phe, Tyr, and several other free amino acids, along with reduced levels of most proteins and severe growth impairment. On the other hand, the *BdHCT1i* lines, with similar levels of reduction in lignin content (∼35% of WT) and no ALs being co-downregulated, exhibited unchanged levels of most amino acids, more balanced overall protein abundance, and a WT growth phenotype. This model is consistent with the increase in amino acid levels, particularly those involved in nitrogen recycling, and the molecular adjustment in pathways of primary metabolism observed in the RNAi lines with major growth defects. Similar mechanisms by which proteins are used as reservoirs of amino acids that can be broken down for the synthesis of nutrients or defense metabolites were previously reported in senescent and stressed plants (Watanabe et al., 2013; Zeier, 2013; Hildebrandt et al., 2015). Considering the abundance, turnover, and major role of ALs in partitioning carbon and nitrogen, it is expected that blocking P/TALs would have major impacts on plant development, as evidenced by the stunted growth of the PAL quadruple mutants in Arabidopsis (Huang et al., 2010). Further studies are needed to test this hypothesis and to determine if trade-offs between the allocation of carbon and nitrogen could be associated with the growth inhibition observed in lignin-modified plants.

One reason that could explain unexpected metabolic/protein relationships observed in our integrated metabolomic and proteomic analysis is the involvement of multiple enzymes in the synthesis of some metabolites. For example, the increased levels of aspartate in the *BdPTAL1i* lines, along with the reduced abundance of cyanoalanine nitrilase, which catalyzes the conversion of 3-cyanoalanine to aspartate, suggest that other enzymatic steps might be involved in the accumulation of aspartate, for instance via β-alanine metabolism. Similarly, the formation of linoleate with downregulated levels of phospholipase A2 may indicate linoleate synthesis from oleic acid desaturation rather than phosphatidylcholine hydrolysis. There is also a possibility of metabolite accumulation due to the downregulation of downstream enzymes. In particular, the hyperaccumulation of Phe and Tyr in the *BdPTAL1i* lines is likely the result of multiple ALs being downregulated rather than from the direct conversion of arogenate, via ADT and ADH, respectively.

Regarding the metabolic changes in the monolignol pathway, the *BdPTAL1i* lines exhibited the largest reductions in lignin content concomitant with reduced levels of all three monolignols, several lignans, and downregulated abundance of 23 proteins annotated in the KEGG database as lignin PRXs. Although LACs are not annotated in KEGG as oxidizing enzymes involved in lignin polymerization, LAC7 was found to be the most significantly downregulated lignin-related protein in the *BdPTAL1i* lines (Figure 3). However, based on current models of monolignol transport by passive diffusion driven by polymerization (Vermaas et al., 2019; Perkins et al., 2022), the reduced lignin levels and absence of oxidizing enzymes in the apoplast could be expected to result in an overaccumulation of monolignols in the cytosol in the *BdPTAL1i* lines. Our data show that suppressing lignin formation by efficiently blocking the early steps of the monolignol biosynthetic pathway results not only in coordinated downregulation of later polymerization steps, but also in a general metabolic shutdown, as evidenced by the large number of repressed proteins in the *BdPTAL1i* and *BdC4H1i* lines (Figure 4B andSupplemental Figures S9 and S10). Together, these data also emphasize the value of integrated proteomic and metabolomic analyses to advance our understanding of lignin metabolism in plants. In view of the importance of microRNAs in the transcriptional repression of lignin LACs in multiple plant species (Lu et al., 2013; Zhang et al., 2013; Wang et al., 2014; Yu et al., 2020; Wei et al., 2021), future studies will also need to incorporate transcriptomic data to better understand the regulation of lignin synthesis.

### SA and other plant growth regulators

SA is a phenolic phytohormone with roles in plant-defense signaling and growth (Shah, 2003; Berens et al., 2017). SA is primarily synthesized from chorismate in the shikimate pathway via isochorismate synthase, but it can also be generated from Phe via PAL, thereby sharing some enzymatic steps with the biosynthesis of lignin (Torrens-Spence et al., 2019; Peng et al., 2021). The high levels of SA observed in the *HCT* KD lines in Arabidopsis and alfalfa were previously suggested to be the cause of dwarfism in these lines (Gallego-Giraldo et al., 2011). Knockout lines of the SA biosynthetic enzyme SID2 in the *HCT* downregulated background alleviated the impaired growth phenotype without affecting lignin content. However, this is not a general mechanism linking lignin biosynthesis and growth phenotypes, because preventing the formation of SA using the *sid2* mutation was not sufficient to recover the stunted growth of the *C3′H* mutants (Bonawitz et al., 2014) or the lignin-deficient *med5* mutants (Mao et al., 2019) in Arabidopsis. Interestingly, however, exogenous ${\text{NH}}_{4}^{+}$ supply reduced the content of SA in the severely dwarf Arabidopsis *siz1* mutants lacking SUMO E3 ligase to WT levels and completely recovered the growth phenotype (Kim et al., 2021). In our study, the levels of SA and two SA and gentisic acid glucosides increased up to three-fold in the *BdHCT1i* lines and decreased up to 2.5-fold in the *BdC3′H1i* lines. However, neither of these lines is dwarf in Brachypodium; the *BdC3′H1i* lines exhibited a 25% biomass reduction, and the *BdHCT1i* lines exhibit a WT growth phenotype. In contrast, blocking the shikimate esters pathway by downregulating *C3′H* and *HCT* led to a highly dwarfed growth phenotype in Arabidopsis and alfalfa (Shadle et al., 2007; Li et al., 2010). Together, these results indicate that increased SA levels are not the cause of the growth phenotype observed in the lignin pathway gene KD lines, and they further suggest that the shikimate shunt is not essential for lignin biosynthesis in Brachypodium.

Some lignin intermediates, such as cinnamate and lignans, were previously suggested to have phytohormone-like activity in promoting germination, cell division, and plant growth (Teutonico et al., 1991; Savy et al., 2017; Vanholme et al., 2019; Ahmad et al., 2021; El Houari et al., 2021), whereas the overaccumulation of flavonoids and ferulate was found to be related to the reduced growth phenotype in some lignin mutants (Besseau et al., 2007; Xue et al., 2015). The levels of cinnamate, ferulate, lignans, and flavonoids are unlikely to be associated with the growth inhibition observed in our study. On the other hand, all RNAi lines, irrespective of growth phenotype, showed wide and non-collapsed xylem vessels (Figure 1), suggesting that the ability to transport water and nutrients might not be the cause of the growth differences of the RNAi lines either.

### Alternative pathways to lignin formation in grasses

A surprising result of the labeling experiments was the lack of label incorporation from ^13^C-Tyr into the B-ring of isoorientin and isovitexin, suggesting that grass C-glycosylated flavones are largely synthesized from *p*-coumarate pools derived from Phe (via PAL and C4H). Flavonoid metabolons formed by weakly bound multi-enzyme complexes generally involving ER-bound cytochrome P450s and soluble flavonoid enzymes have been reported in multiple plant species (Zhao, 2015; Fujino et al., 2018; Gou et al., 2018). Our results further support the concept of two different *p*-coumarate pools derived from Phe and Tyr (Barros et al., 2016), and they suggest that an early pathway metabolon specifically directs *p*-coumarate pools generated from Phe into C-glycosylated flavonoid biosynthesis. An alternative explanation is that C-glycosyl flavone synthesis occurs in cell types distinct from where PTAL1 is localized. Studies are needed to determine whether the compartmentalization of this pathway occurs at the cellular or subcellular level.

The flexibility of the phenylpropanoid pathway was evident from examining the *BdPTAL1i* lines, where most lignin intermediates were preferentially labeled from ^13^C_9_-Phe. Blocking PTAL1 increased the incorporation of ^13^C_9_-Phe into *p*-coumarate, caffeate, ferulate, and sinapate, suggesting that a routing of the pathway via free phenolic acids depends on substrate availability. The labeled precursor feeding experiments also pointed to the operation of some enzymatic steps that are still poorly characterized. For example, the high levels of ^13^C_9_-Tyr incorporated into *p*-coumarate, sinapate, sinapaldehyde, sinapyl alcohol, and S-units in the *Bd C3′H1i* lines support the notion of a Tyr-derived lignin pathway for the synthesis of S-lignin via sinapate. An efficient 4CL-like enzyme with a preference for sinapate has been identified in Arabidopsis (Hamberger and Hahlbrock, 2004) but still remains to be characterized in grasses. Additionally, Arabidopsis F5H (CYP84A1) shows preferential kinetics toward coniferaldehyde and coniferyl alcohol rather than ferulate (Humphreys et al., 1999), but the kinetics toward feruloyl CoA have not been reported to date, and a dedicated F5H enzyme with preferential affinity for ferulate has not yet been identified in plants.

The Tyr- and Phe-derived metabolomes were recently examined in a study combining ^13^C-labeling and LC–MS untargeted metabolomics in sorghum (Simpson et al., 2021). The results of this study in sorghum provide not only further evidence for the existence of distinct metabolic pools associated with the PAL and TAL pathways, but they also reveal a preferential incorporation of Phe and Tyr into different phenylpropanoids in a tissue-specific manner. Unfortunately, monolignols and most lignin precursors were not identified, preventing us from making any further conclusions regarding potential parallel routes to lignin formation in sorghum.

The labeling experiments also showed high levels of Phe-labeled sinapaldehyde (27%) that were not reduced by CAD to form Phe-labeled sinapyl alcohol (10%), particularly in WT plants (Supplemental Figure S17). Sinapaldehyde can also be a substrate of ALDH, which has been genetically characterized in Arabidopsis (*AtALDH*, At3g24503) and kinetically tested in crude protein extracts from several plant species including grasses (Nair et al., 2004). ALDHs are capable of oxidizing sinapaldehyde and coniferaldehyde to sinapic and ferulic acids, respectively. Brachypodium possesses one bona fide *AtALDH* homolog (Bradi1g43770) with relatively abundant protein levels (∼10% of PTAL1) in mature WT stems. Three additional ALDH homologs (Bradi1g37090, Bradi2g42360, and Bradi2g42380) with >70% similarity to *AtALDH* displayed protein abundance levels ˂1% of PTAL1 in lignified stem tissues. This observation suggests that at least part of the pools of ferulate and sinapate derived for ^13^C_9_-Phe could be formed by direct NADP+-dependent oxidation from the corresponding aldehydes, rather than being precursors of those aldehydes and hence the G and S monolignols.

It is well established that grass cell walls possess significant levels of arabinoxylans ester-linked to ferulic, *p*-coumaric, and sinapic acids (Hartley et al., 1990; de O Buanafina, 2009; Bunzel, 2010). This metabolic sink for free-hydroxycinnamic acids could reflect the high abundance of PTAL, C3H, and COMT and the presence of ALDH suggested by the isotope labeling data. Additionally, our ^13^C_9_-Tyr feeding experiments implicated *p*-coumarate, caffeate, ferulate, and sinapate in sinapyl alcohol biosynthesis, and COMT preparations from grasses efficiently catalyzed the methylation of both caffeate and 5-hydroxyferulate (Shimada et al., 1973; Barros et al., 2019). Moreover, the F5H (CYP84A1) knockout mutants in rice display mostly unaffected levels of lignin deposition and no impact on the grass-specific γ-*p*-coumaroylated monomers (Takeda et al., 2018, 2019), suggesting that a route to sinapyl alcohol acting at the free acids level may exist in lignified grass tissues. The biological significance of these parallel pathways remains to be determined. Future studies in this area will benefit from the integration of multiomic approaches coupled with time-series isotope labeling experiments in a tissue- and cell-specific manner.

In conclusion, our study showed that (1) PTAL is one of the most abundant proteins in lignifying tissues in grasses; (2) there is crosstalk between lignin biosynthesis and primary metabolic pathways, likely mediated through the NH_3_/${\text{NH}}_{4}^{+}$ released from the P/TAL reaction that is recycled for the synthesis of amino acids; (3) the canonical pathway via shikimate ester intermediates (shikimate shunt) is not essential for lignin formation; and (4) there are parallel pathways to lignins in grasses, with a route that preferentially converts Tyr to soluble free hydroxycinnamates destined for S-units of lignin, in parallel to a dicot-like Phe-derived lignin pathway for the synthesis of G-lignin and S-lignin involving the shikimate shunt. Our data further suggest that a trade-off between the allocation of carbon for lignin synthesis and nitrogen recycled from the P/TAL reaction for the synthesis of nucleotides, amino acids, and proteins could be associated with the growth inhibition observed in lignin-modified Brachypodium plants.

## Materials and methods

### Plant materials and growth conditions

Brachypodium (*B.distachyon*) inbred line Bd21-3 was used as the WT control and genetic background for transformation. Plants were grown in half-gallon pots containing Metro-Mix 360 potting soil, regularly watered with 24-8-16 soluble fertilizer, and moved to minimize spatial variation of growth conditions. Plants were grown in both growth chamber and greenhouse conditions. The growth chamber conditions were 16-h light: 8-h dark photoperiod, cool-white fluorescent lighting at a level of 100 µEm^−2^ s^−1^, and temperatures of 22°C during the day and 18°C at night. The greenhouse conditions were 25°C–28°C day/22°C night with a 14-h photoperiod of natural irradiance with supplemental lighting to a minimum level of 120 µmol m^−2^ s^−1^ PAR. Multiple R0 transgenic lines were generated from transformed callus and grown in growth chambers. Stem internodes of R0 lines were harvested at 45 days after germination for RNA isolation and RT-qPCR analysis as described below. Seeds from the R0 lines with minimal off-target transcript level effects were selected as parental lines for the R1 generation. Individual R1 lines for each RNAi construct were screened by RT-qPCR at 45 days after germination, and R2 seeds were harvested from senesced R1 plants. All five lignin pathway gene R2 KD lines were planted along with WT controls and grown under greenhouse conditions. Five different 1/2-gallon pots were used to grow ∼25 seeds of each genotype until 30 days after germination when the first inflorescence was emerging, equivalent to the booting stage in the BBCH-scale for cereals (Lancashire et al., 1991). Plants were then separated into leaves (only leaf blades) and stems (with leaf sheaths) and weighed in the greenhouse using a digital precision scale to obtain the total above-ground biomass. Tissues were stored at −80°C for subsequent analysis.

### Generation of Brachypodium RNAi lines

A vector designed to generate RNAi constructs for monocot plants (pANIC8A) was used for Agrobacterium (*Agrobacteriumtumefaciens*)-mediated transformation following previous protocols with some minor modifications (Vogel and Hill, 2008; Mann et al., 2012). Five different RNAi constructs targeting *PTAL1*, *PAL2*, *C4H1*, *C3′H1*, and *HCT1* gene transcripts were generated by amplifying the nucleotide fragments from Brachypodium cDNA from developing stems using the primers listed in Supplemental Table S1. The five amplified fragments of *PAL2* (287 bp), *PTAL1* (371 bp), *C4H1* (249 bp), *C3′H1* (272 bp), and *HCT1* (259 bp) were introduced into the destination vector pANIC8A using Gateway cloning technology. The conserved coding regions of *PAL/PTAL*, *C4H*, and *HCT* family genes were analyzed to determine the specificity of the target RNAi fragments. C3′H1 is a unique enzyme in Brachypodium, allowing the target region to be easily chosen. Gene sequence analysis, primer design, and vector assembly were performed with Geneious (https://www.geneious.com) and SnapGene (https://www.snapgene.com) software. Agrobacterium strain EHA105 harboring the recombinant plasmids was cultured at 28°C in LB medium with 25 mg/L rifampicin and 75 mg/L spectinomycin until OD600 = 0.6. Immature Brachypodium embryos picked from newly filled seed were sterilized and transfected with the Agrobacterium cell suspensions for 10 min. The EHA105 suspension was removed by pipetting, and transfected calli were placed on callus initiation medium containing Linsmaier and Skoog medium (4.43 g L^−1^), sucrose 30 g  L^−1^ and CuSO_4_ 0.6 mg L^−1^ at pH = 5.8 with 5 mg/mL 2,4-dichlorophenoxyacetic acid added after autoclaving, and incubated in the dark at 24°C. Three days after transformation, the calli were transferred to callus initiation medium plates supplemented with 150 mg L^−1^ timentin and 40 U mL^−1^ hygromycin B. Calli were individually transferred once a week for 3–4 weeks to new callus initiation medium, and independent healthy whitish friable callus pieces were then cultured on differentiation medium (LS 4.43 g L^−1^, maltose 30 g L^−1^ and phytagel 2.5 g L^−1^ pH = 5.8, supplemented with 9.3 μM kinetin, 150 mg L^−1^ timentin, and 40 U mL L^−1^ hygromycin B. Once transgenic shoots with three to four leaves appeared (typically 2–6 weeks after transferring to differentiation medium), plantlets were then transferred to rooting medium (containing Murashige and Skoog [MS] medium w/vitamins 4.42 g L^−1^, sucrose 30 g L^−1^ and Phytagel 2 g L^−1^ pH = 5.8 supplemented with 150 mg L^−1^ timentin). Developed R0 generation plantlets were transferred to greenhouse conditions in half-gallon pots and grown as described above.

### RT-qPCR

RNA was extracted from frozen stem internode samples (∼100 mg) with Trizol (Thermo Fisher Scientific, Waltham, Massachusetts, USA) according to the manufacturer’s protocol. Total RNA (∼3 µg) was quantified with a NanoDrop ND-1000 spectrophotometer (NanoDrop Technologies, Wilmington, Delaware, USA) and treated with an Invitrogen TURBO DNA-free kit (Fisher scientific) to remove genomic DNA. First-strand cDNA was synthesized using a High-Capacity cDNA Reverse Transcription Kit (Thermo Fisher) following the manufacturer’s instructions. RT-qPCR was used to quantify the expression of selected genes and determine silencing efficiency in R0 and R1 transgenic plants. Brachypodium *Tubulin* (Bradi1g10150) was used as a housekeeping gene. RT-qPCR was performed using Power SYBR Green Master Mix (Thermo Fisher) in a QuantStudio 6 Flex Real-Time PCR System (Thermo Fisher). The RT-PCR cycling conditions were as follows: 95°C for 10 min followed by 40 cycles of 95°C for 15 s, 60°C for 30 s and 72°C for 30 s. Results were analyzed by the comparative 2^–ΔΔCt^ method (Schmittgen and Livak, 2008). Each experiment comprised at least three independent replicates from stem internode samples harvested from different plants at 45 day after germination and three technical replicates for each cDNA sample. Genomic DNA was extracted from the leaves of R2 generation transgenic plants using the cetyl trimethylammonium bromide method (Rogers and Bendich, 1989) for screening RT-PCR detection and confirming the presence of the hygromycin resistance gene of the pANIC 8A silencing vector. PCR-positive plants were used for further phenotypic characterization.

### Histochemistry of lignin deposition

Transverse sections (∼100 µm) of the first internode were made using razor blades and an HM 650 V vibrating blade microtome (Thermo Fisher). The sections were transferred to 24 well tissue culture plates containing distilled water. Fluorescence microscopy was performed using an EVOS FL Cell Imaging System equipped with a DAPI led light cube at 360 nm excitation and 447 nm emission to visualize lignin autofluorescence. The cross-sections were subjected to phloroglucinol staining using Wiesner reagent. The sections were treated with the reagent (80 µM phloroglucinol-ethanol in 14 mM HCl) for 2 min to stain and visualize lignin. Stained cross-sections were mounted on microscope slides and visualized using an EVOS XL Core Imaging System (Thermo Fisher).

### Cell wall preparation and thioacidolysis of lignins

Alcohol insoluble residues (AIRs) of whole cell wall material were prepared from 200 mg of ground frozen stem internodes by sequential extraction with 100% methanol (once), chloroform/methanol (2:1) (twice), 100% methanol (once), and water (twice) at room temperature and lyophilized overnight. Thioacidolysis followed by gas chromatography–MS (GC–MS) quantification of the trimethylsilyl (TMS) derivatives of the lignin-derived monomers was carried out on 10 mg of AIR using a previously published protocol (Chen et al., 2021). GC–MS analyses were carried out on a Hewlett–Packard 7890A gas chromatograph with a 5975C series mass selective detector with column DB-5MS 60 m, 0.25 mm, 0.25 µm (Agilent Technologies, Santa Clara, California, USA). The TMS derivatives of the thioethylated coumaryl (H), coniferyl (G), and sinapyl (S) monomers were identified at 239, 269, and 299 *m*/*z*, respectively. The extracted ion peak areas of each lignin monomer were obtained and transformed to micromoles per gram AIR by considering the relative concentration of the internal standard docosane. Total thioacidolysis lignin yields were calculated as the sum of the lignin-derived thioacidolysis monomers recovered from H, G, or S β-O-4-linked lignin subunits and used to obtain the relative percentage lignin composition.

### Acetyl-bromide-soluble lignin

The acetyl-bromide-soluble lignin procedure was used to determine the lignin content of the AIR samples as previously described (Moreira-Vilar et al., 2014). Briefly, AIR (∼20 mg) was incubated with 72% H_2_SO_4_, 50°C, 10 min with shaking. The samples were autoclaved at 121°C for 30 min and centrifuged at 3,500 rpm to separate the soluble and insoluble lignin fractions. A 100 µL aliquot from the soluble fraction was transferred to 96-well plates and the absorbance measured at 280 nm using a Synergy HTX Multi-Mode Microplate Reader (BioTek Instruments, Winooski, Vermont, USA). The lignin content was determined using a calibration curve made with pure lignin standard solutions (Sigma, St. Louis, Missouri, USA; 370959) and expressed as milligram per gram AIR.

### LC–MS/MS quantification of soluble phenylpropanoids

To estimate the levels of phenylpropanoid pathway intermediates, lyophilized stem internode samples (∼20 mg) were weighed into centrifuge tubes and extracted with 80% methanol. The extracts were then transferred to 3 kDa Amicon columns, spun at 14,000 × g for 30 min, and the eluted extracts used for LC–MS/MS analyses. A 10 µL aliquot of the extracts was diluted in 90 µL of acetonitrile/water (60:40 v/v) solution and transferred to HPLC vials. The detection and quantification of phenylpropanoid intermediates was conducted as previously described (Cocuron et al., 2017, 2019). Briefly, the compounds were separated using an Agilent 1290 Infinity II liquid chromatography system coupled to a hybrid Triple Quadrupole 6500+ triple quadrupole from ABSciex. The extracts were kept at 10°C in an auto-sampler. The metabolites were resolved at 30°C using a reverse phase C18 Symmetry column (4.6 × 75 mm; 3.5 µm) associated with a Symmetry C18 pre-column (3.9 × 20 mm; 5 µm) from Waters. The liquid chromatography gradient was made of 0.1% (v/v) acetic acid in acetonitrile (A) and 0.1% (v/v) acetic acid in water (B). The total LC–MS/MS run was 15 min with a flow rate of 800 µL/min. The following gradient was applied to separate the phenolic compounds: 0–1 min 85% B, 1–7 min 42% B, 7–9 min 20% B, 9–9.1 min 15% B, 9.1–12 min 15% B, 12–12.1 min 85% B, and 12.1–15 min 85% B. Metabolite detection was conducted using an AB Sciex hybrid Triple Quadrupole/Ion trap mass spectrometer QTRAP 6500+. Electrospray ionization with polarity switch was utilized to acquire mass spectra of the different analytes. The settling time between each polarity was 15 msec. The source parameters such as curtain gas, temperature, nebulizer gas (GS1), heating gas (GS2), and collision activated dissociation (CAD) were kept constant during MRM survey scan. The dwell time was set to 10 msec. Analyst 1.7 software from AB Sciex was used to acquire and process the LC–MS/MS data. The injection volume was 20 µL for the extracts or 5 µL for the external standard mixture, and the needle was rinsed with 50% methanol between injections. Compounds were identified and quantified using a mixture of known external standards run at the same time as the biological extracts. Stem internodes from three different plants harvested at 30 day after germination were processed for each genotype.

### Proteomics sample preparation

Frozen stem internode samples (∼500 mg) were solubilized in 1 mL lysis buffer (4% sodium dodecyl sulfate and 10 mM dithiothreitol in 100 mM ${\text{NH}}_{4}^{+}$ bicarbonate). The tissue samples were vortexed and placed in a heat-block for 5 min at 90°C. The samples were further disrupted by sonication (30% amplitude, 10 s pulse with 10 s rest, 1 min total pulse time) and boiled for an additional 5 min at 90°C. The samples were centrifuged at maximum speed for 5 min and the supernatants were collected. The samples were then alkylated by incubating with 30 mM iodoacetamide for 15 min in the dark to prevent reformation of disulfide bonds. Proteins were then extracted using a chloroform–methanol extraction protocol (Jiang et al., 2004) using methanol, chloroform, and LC/MS grade water in the ratio of 4:1:3. This was followed by 10 min of centrifugation at 4,000 g. The protein layer was extracted and washed using 100% methanol. The protein layer was air-dried, and the dry pellet was reconstituted in 300 µL sodium deoxycholate solution made up of 2% sodium deoxycholate in 100 mM ${\text{NH}}_{4}^{+}$ bicarbonate. Protein concentration was measured using a NanoDrop OneC spectrophotometer (Thermo Scientific). Each sample was adjusted to be 250 µg of total protein. Proteins were digested with two separate and sequential aliquots of sequencing grade trypsin (Promega, Madison, Wisconsin, USA) of 1:75 (wt/wt) protein:trypsin ratio. The samples were first digested for 3 h, followed by dilution of sodium deoxycholate to 1% for overnight digestion. After digestion, sodium deoxycholate was removed by precipitating with 1% formic acid, followed by an ethyl acetate wash for a total of three times. The samples were then lyophilized/dried in a SpeedVac concentrator. Peptide samples were desalted on Pierce peptide desalting spin columns (Thermo Scientific) as per the manufacturer’s instructions. After speed-vac concentration, dry samples were suspended in 100 µL of 0.1% formic acid solution. Peptide concentrations were then measured using a NanoDrop, and 2 μg of protein of each sample was used for LC–MS/MS measurement.

### Proteomic LC–MS/MS analysis

All samples were analyzed on a Q Exactive Plus mass spectrometer (Thermo Fisher Scientific) coupled with an automated Proxeon EASY-nLC 1200 liquid chromatography pump as previously described (Villalobos Solis et al., 2020). In brief, peptides were separated on an in-house-pulled nanospray emitter of 75 μm inner diameter packed with 30 cm of 1.7 μm of Kinetex C18 resin (Phenomenex). For each sample, a single 2 μg injection of peptides was loaded in buffer A (0.1% formic acid, 2% acetonitrile) and eluted with a linear 210 min organic gradient, washed, and re-equilibrated: 0%–2% solvent B over 27 min, 2%–25% solvent B over 148 min, 25%–50% solvent B over 10 min, 50%–0% solvent B over 10 min, hold at 0% solvent B for 15 min. MS data were acquired with Thermo Xcalibur software using the top 10 data-dependent acquisition.

### Proteome database searching

All MS/MS spectra collected were processed in Proteome Discoverer version 2.3 using MS Amanda version 2.0 (Dorfer et al., 2014) and Percolator (Käll et al., 2007). Spectral data were searched against the *B.distachyon* reference proteome database from UniProt to which common laboratory contaminants were appended. The following parameters were set up in MS Amanda to derive fully tryptic peptides: MS1 tolerance = 5 ppm; MS2 tolerance = 0.02 Da; missed cleavages = 2; Carbamidomethyl (C, +57.021 Da) as the static modification; and oxidation (M, +15.995 Da) as dynamic modifications. The percolator FDR threshold was set to 1% at the PSM and peptide levels. FDR-controlled peptides were then quantified according to the chromatographic area-under-the-curve and mapped to their respective proteins. Areas were summed to estimate protein-level abundance.

### Protein data analysis

For differential abundance analysis of proteins, the protein table with at least two peptides evidence was exported from Proteome Discoverer. Proteins were filtered to remove stochastic sampling; all proteins present in three out of four biological replicates in any condition were considered valid for quantitative analysis. Data were log2 transformed, LOESS normalized between the biological replicates, and mean-centered across all the conditions using InfernoRDN software (Polpitiya et al., 2008). Missing data were imputed by random numbers drawn from a normal distribution (width = 0.3 and downshift = 2.8) using Perseus software (http://www.perseus-framework.org) (Tyanova et al., 2016). Protein sequence homology inherent to plant proteomes can lead to protein identifications that cannot be qualitatively or quantitatively differentiated. This ambiguity was addressed by reporting protein groups that are clustered based on the sequence homology (>90%) using uclust version 5.0 (Edgar, 2010).

### Identification of lignin biosynthetic proteins in Brachypodium

All members of the Arabidopsis lignin biosynthetic gene family were retrieved from TAIR, and BLAST searches for Brachypodium homologs were performed based on DNA sequence similarities obtained from Phytozome (https://phytozome.jgi.doe.gov). Homologous genes with similarities ˂60% (50% for CSE and cytochrome P450s) were excluded in these analyses. The first Arabidopsis homolog was used as the bait gene. For example, for AtPALs, *AtPAL1* (AT2G37040) was used as the bait gene. Brachypodium gene and protein IDs were retrieved from UniProt (https://www.uniprot.org/). In silico expression levels of the Brachypodium genes were qualitatively rated based on the expression signal observed in microarray data from 35-day-old first stem Brachypodium internodes from BAR (http://bar.utoronto.ca/). The raw protein intensity peak areas obtained from four replicates of actively lignifying mature WT Brachypodium stems were used to report the levels of other lignin proteins as percentages of the most abundant lignin protein PTAL1 (Bradi3g49250).

### GC–MS untargeted metabolomics

For GC–MS untargeted metabolic profiling, ∼75 mg of frozen ground mature Brachypodium stem samples were weighed into microcentrifuge tubes and extracted twice overnight with 2 mL of 80% ethanol. Sorbitol (50 μL at 1 mg mL^−1^) was added to the first extract as an internal standard to correct for volume changes and variations in extraction or derivatization efficiency. The samples were extracted in a tube rotator overnight at room temperature and then centrifuged at 3,200 g for 20 min. The supernatants were transferred into scintillation vials and stored at −20 °C. A 1.5 mL aliquot was dried under nitrogen, dissolved in 0.5 mL acetonitrile, and silylated with 0.5 mL of N-methyl-*N*-trimethylsilyltrifluoroacetamide with 1% trimethylchlorosilane to generate TMS derivatives. Samples were injected (1 µL) into an Agilent 7890A gas chromatograph coupled to a 5975 C inert XL mass spectrometer operated in electron impact ionization mode (EI; 70 eV) with a 50–650 Da scan range as previously reported (Abraham et al., 2016). The peak areas of each compound were extracted using a characteristic mass-to-charge (*m*/*z*) ratio to minimize interference with the *m*/*z* of co-eluting metabolites. Peaks were quantified by area integration and normalized to the intensity of the internal standard recovered and the amount of sample extracted, derivatized, and injected in the GC–MS. The NIST database library and a user-created database of >2,400 metabolites were used to identify the metabolites in the samples. A total of 84 metabolites were identified, including several unidentified metabolites that were designated by their retention times and key *m*/*z* ratios: N metabolite.1 (RT = 9.88; 98 288 390 *m*/*z*); N metabolite.2 (RT = 12.09; 232 449 464 *m*/*z*); N metabolite.3 (RT = 9.78; 174 329 314 *m*/*z*); N metabolite.4 (RT = 10.74; 173 156 116 335 *m*/*z*); N metabolite.5 (RT = 10.07; 174 114 232 *m*/*z*); N-metabolite.6 (RT = 8.86; 188 262 100 *m*/*z*); glucuronic acid conjugate (RT = 19.04; 219 365 *m*/*z*); ascorbic acid conjugate (RT = 15.88; 332 404 214 *m*/*z*); S lignan glycoside (RT = 16.39; 311 *m*/*z*); G lignan.1 (RT = 16.12; 411 *m*/*z*); and G lignan.2 (RT = 17.39; 411 *m*/*z*).

### Construction of KEGG metabolic pathways

We first used volcano plots with a threshold of fold-change (FC) > 2 and *P*-value < 0.05 to identify highly significant differentially expressed metabolites and proteins. All 84 metabolites identified by GC–MS untargeted metabolomics were assigned to their specific pathways and plotted in the Brachypodium KEGG metabolic map (https://www.genome.jp/kegg-bin/show_pathway?bdi01100). These metabolic maps also incorporated the levels of soluble phenylpropanoid metabolites quantified by LC–MS/MS. The concentration of each metabolite in the RNAi lines was compared with that of WT plants, and the differentially regulated metabolites were colored in the KEGG maps of each RNAi line in dark blue if accumulated, dark green if depleted, or gray if the concentration remained unchanged compared with WT plants. A total of 2,268 Brachypodium genes associated to specific metabolic pathways were extracted from the KEGG database, and the corresponding protein abundance levels in mature stem internodes were obtained for the RNAi lines and control plants. Induced and repressed proteins (FC > 2 and *P*-value < 0.05) in each RNAi line were associated to their corresponding enzymatic steps and colored in dark blue if repressed or light green if induced. Metabolites were highlighted in red when changes also occurred in their biosynthetically related proteins.

### Stable isotope labeling experiments

R2 transgenic lines and WT plants were grown on culture tubes containing MS medium supplemented with 3% sucrose (pH 5.8) in 0.5% Phytagel and 0.1 mM ^13^C_9_-Tyr or ^13^C_9_-Phe (Cambridge Isotope Laboratories, Tewksbury, Massachusetts, USA). Control experiments with WT plants using unlabeled Phe and Tyr were conducted in a previous study (Barros et al., 2016). Plants were grown under continuous light conditions at 22°C and harvested at 30 days after germination. Roots, stems and leaves of 10–20 plants for each individual RNAi line and WT controls were carefully separated and stored at −80°C until use.

To estimate the levels of ^13^C-labeled monolignols, roots and stems from transgenic and control plants were lyophilized, the lignin extracted, and the peak areas of the thioacidolysis products of lignin (H-, G-, and S-units) identified by GC–MS as described above. Monolignol fragments lose two carbons of the side-chain during GC–MS fragmentation after thioacidolysis; therefore, the m0 and m7 masses were obtained and used to calculate the percentage of label incorporated into each monolignol as follows: peak areas of labeled monolignols (m7) divided by the total (labeled m0 + unlabeled m7) monolignol levels in the samples and multiplied by 100.

To estimate the levels of ^13^C-labeled monolignol pathway intermediates, lyophilized root samples (∼10 mg) were prepared for LC–MS/MS targeted metabolomics as described above. Both ^12^C- and ^13^C-phenylpropanoid intermediates were simultaneously detected and quantified as precursor ion/product ion pair (m_0_ and m_9_ isotopomers) using multiple reaction monitoring-based MS (Supplemental Table S2). The percentage of labeled precursors (^13^C_9_-Tyr ^13^C_9_-Phe) incorporated into the lignin intermediates was obtained as the concentration of labeled metabolite (m9) divided by the total (labeled m0 + unlabeled m9) metabolite concentration in the samples and multiplied by 100.

### Data analysis

One-way ANOVA was used followed by a Dunnett’s post hoc test as indicated for data analysis for multiple comparisons. Statistical tests were performed using Prism software. Bar plots show means ± SEM, and *P* ≤ 0.05 was considered statistically significant. Box plots indicate the median (center lines) and interquartile range (hinges), and whiskers represents min and max values. The log_2_ FC and -log_10_ of the *P*-value from Student’s *t* tests was calculated using Microsoft Office Excel 2019. All ANOVA and *t* test results are provided in Supplemental Data Set S5.

## Accession numbers

Sequence data for the genes and proteins targeted for RNAi silencing in this article can be found in the EMBL/GenBank/UniProt databases using the following accession numbers: *PAL2* (Bradi3G49260, I1IBR6), *PTAL1*(Bradi3G49250, I1IBR5); *C4H1*(Bradi2G53470, I1HSV5), *C3’H1* (Bradi2G21300, I1HI50), *HCT1* (Bradi5G14720, I1IZB2). Gene model IDs of all lignin biosynthetic genes from both the reference genome (*B.distachyon* Bd21) and the genome of Brachypodium inbred line Bd21-3 are provided in Supplemental Data Set S1.

The raw sequence, msf and tab files for the MS data sets are available in the ProteomeXchange Consortium (https://www.proteomexchange.org/) via the MassIVE repository (https://massive.ucsd.edu/) under the dataset identifiers PXD026665 (ProteomeXchange) and MSV000087614 (MassIVE).

## Supplemental data

The following materials are available in the online version of this article.

**Supplemental Figure S1.** Nitrogen recycling in the phenylpropanoid pathway in grasses.

**Supplemental Figure S2.** RNA interference sequences.

**Supplemental Figure S3.** qRT-PCR selection of Brachypodium RNAi lines used in this study.

**Supplemental Figure S4.** Phenotypic effects of lignin modification in Brachypodium RNAi lines.

**Supplemental Figure S5.** Protein levels in mature stems from WT Brachypodium.

**Supplemental Figure S6.** Abundance levels of several early lignin pathway enzymes in different plant tissues and species obtained from this work and previous studies.

**Supplemental Figure S7.** Metabolic KEGG maps overlaid with primary and secondary metabolites identified in this work.

**Supplemental Figure S8.** Metabolic shifts as a result of downregulation of the lignin biosynthetic pathway gene *PAL2.*

**Supplemental Figure S9.** Metabolic shifts as a result of downregulation of the lignin biosynthetic pathway gene *PTAL1*.

**Supplemental Figure S10.** Metabolic shifts as a result of downregulation of the lignin biosynthetic pathway gene *C4H1.*

**Supplemental Figure S11.** Metabolic shifts as a result of downregulation of the lignin biosynthetic pathway gene *C3′H1.*

**Supplemental Figure S12.** Metabolic shifts as a result of downregulation of the lignin biosynthetic pathway gene *HCT1.*

**Supplemental Figure S13.** Metabolite levels in mature stem tissues of Brachypodium RNAi lines.

**Supplemental Figure S14.** Heatmap of metabolite levels in mature stem tissues of Brachypodium RNAi lines.

**Supplemental Figure S15.** Organ-specific expression of monolignol pathway genes in Brachypodium WT plants.

**Supplemental Figure S16.** Lignin composition of in vitro grown Brachypodium RNAi lines fed with ^13^C-labeled precursors.

**Supplemental Figure S17.** Proportion of ^13^C9-Phe (blue) and ^13^C9-Tyr (green) labeled lignin intermediates in the roots of Brachypodium RNAi lines.

**Supplemental Table S1.** Oligonucleotides used in this work.

**Supplemental Table S2.** LC/MS-MS parameters used for the identification of intermediate metabolites in the lignin biosynthetic pathway.

**Supplemental Data Set S1.** Closest homologs to Arabidopsis lignin biosynthetic genes in the model grass Brachypodium and protein abundances in stems from 30 days-old WT Brachypodium plants.

**Supplemental Data Set S2.** Lignin biosynthetic protein intensity levels in stems from 30 days-old WT controls and RNAi KD Brachypodium lines.

**Supplemental Data Set S3.** Top 60 most abundant proteins in stems from 30 days-old WT Brachypodium.

**Supplemental Data Set S4.** Overall protein abundance levels of PTAL, PAL, COMT, and C3H/APX in several plant tissues and species obtained from published studies and our work.

**Supplemental Data Set S5.** ANOVA and *t* test results.

## Supplementary Material

koac171_Supplementary_Data

## Notes

R.A.D, T.J.T., R.L.H., and J.B. conceived and designed the study. J.C.S.-Y. generated and performed the qRT-PCR selection of the Brachypodium RNAi lines. J.B., H.K.S., and P.E.A. prepared the samples for proteomics and performed the proteomic analysis. T.J.T. and N.E. conducted the GC–MS metabolomics analysis. J.B. analyzed the data. J.B and R.A.D. wrote the manuscript and all authors read and approved the final manuscript.

The authors responsible for the distribution of materials integral to the findings presented in this article in accordance with the policy described in the Instructions for Authors (https://academic.oup.com/plcell) are: Jaime Barros (Jaime.Barros@missouri.edu) and Richard A. Dixon (Richard.Dixon@unt.edu).
