# Supplementary material for: Proteomic and metabolic disturbances in lignin-modified Brachypodium distachyon
Source: Plant Cell. 2022 Jun 7;34(9):3339–63. doi: 10.1093/plcell/koac171 (PMC9421481; doi:10.1093/plcell/koac171)
Supplement: koac171_Supplementary_Data [file koac171_supplementary_data.zip › tpc.22.00295_SupplementalFiguresandTables.pdf]

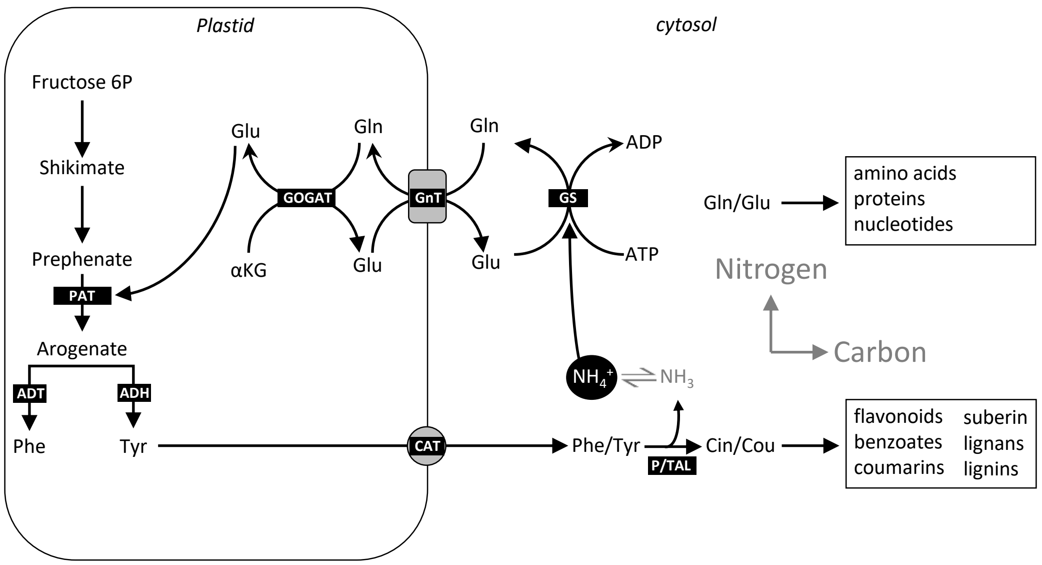

Supplemental Figure S1. Nitrogen recycling in the phenylpropanoid pathway in grasses (Supports Figure 6). Up to 40% of the dry weight of grasses derives from phenylalanine (Phe) and tyrosine (Tyr). Each phenylalanine/tyrosine ammonia-lyase (P/TAL) reaction to form cinnamate (Cin) and coumarate (Cou) releases an equimolar amount of ammonia (NH_3) in equilibrium with cytosolic ammonium (NH_4^+) ions, which are primarily recycled into glutamine (Gln) and glutamate (Glu) via the glutamine synthetase (GS) and glutamate synthase (GOGAT) cycle. The NH_4^+ recycled from the early phenylpropanoids pathway represents 2/3 of the total primary nitrogen assimilated in plants, including soil uptake and atmospheric fixation (Raven et al., 1992; Razal et al., 1996). GnT, glutamine/glutamate translocator; PAT, prephenate aminotransferase; ADT, arogenate dehydratase; ADH, arogenate dehydrogenase; CAT, cationic amino acid transporter; αKG, alpha-ketoglutarate.

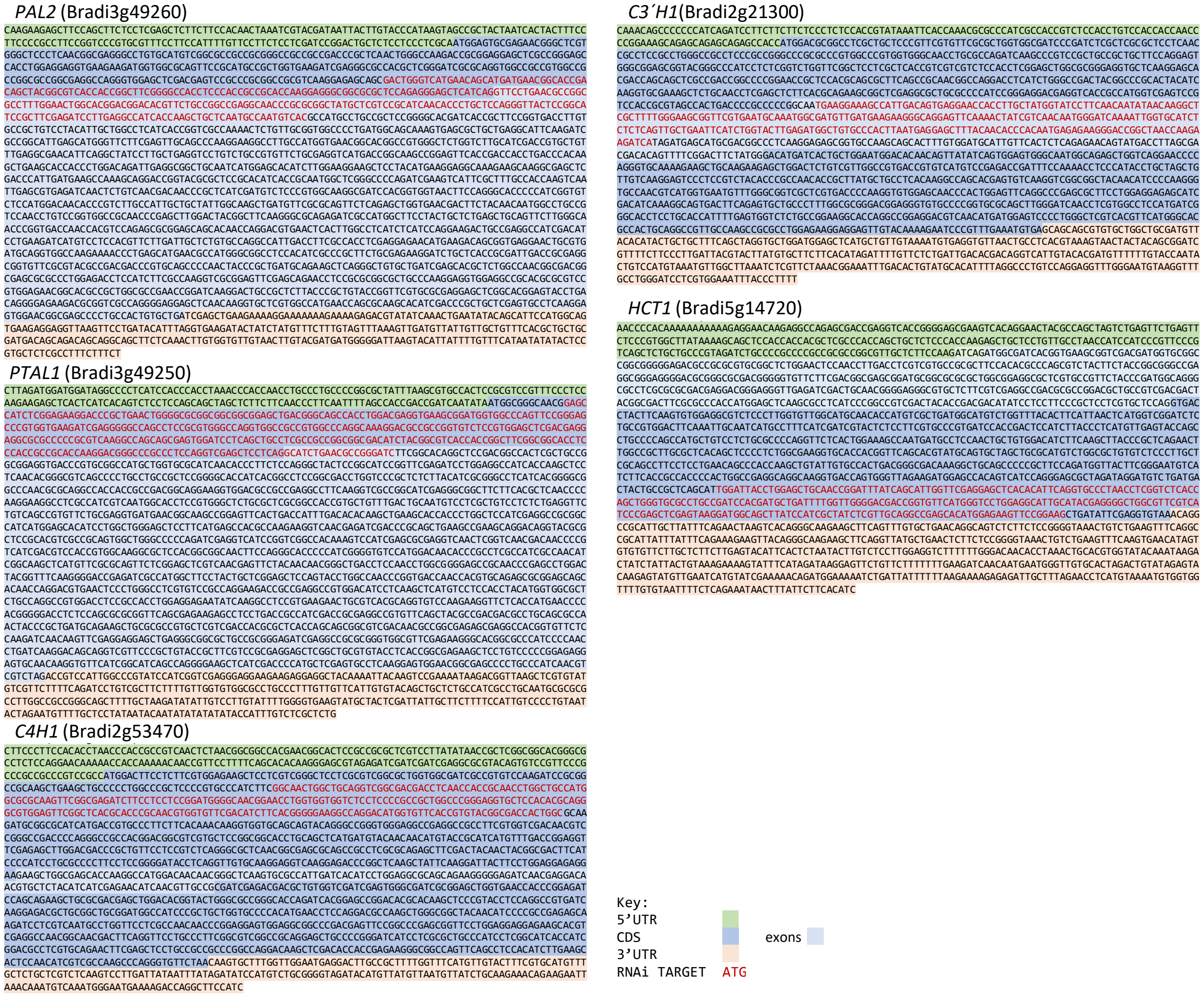

Supplemental Figure S2. RNA interference sequences (Supports Figure 1). Nucleotide sequences of target lignin genes showing 5'-UTR (green shade) and 3´-UTR regions (orange shade), coding sequence (CDS, blue shade), and target sequence for gene silencing (red letters). Custom pANIC8A vectors were cloned to target messenger RNA of *PAL2*, *PTAL1*, *C4H1*, *C3´H1* and *HCT1* genes, as described in the methods section.

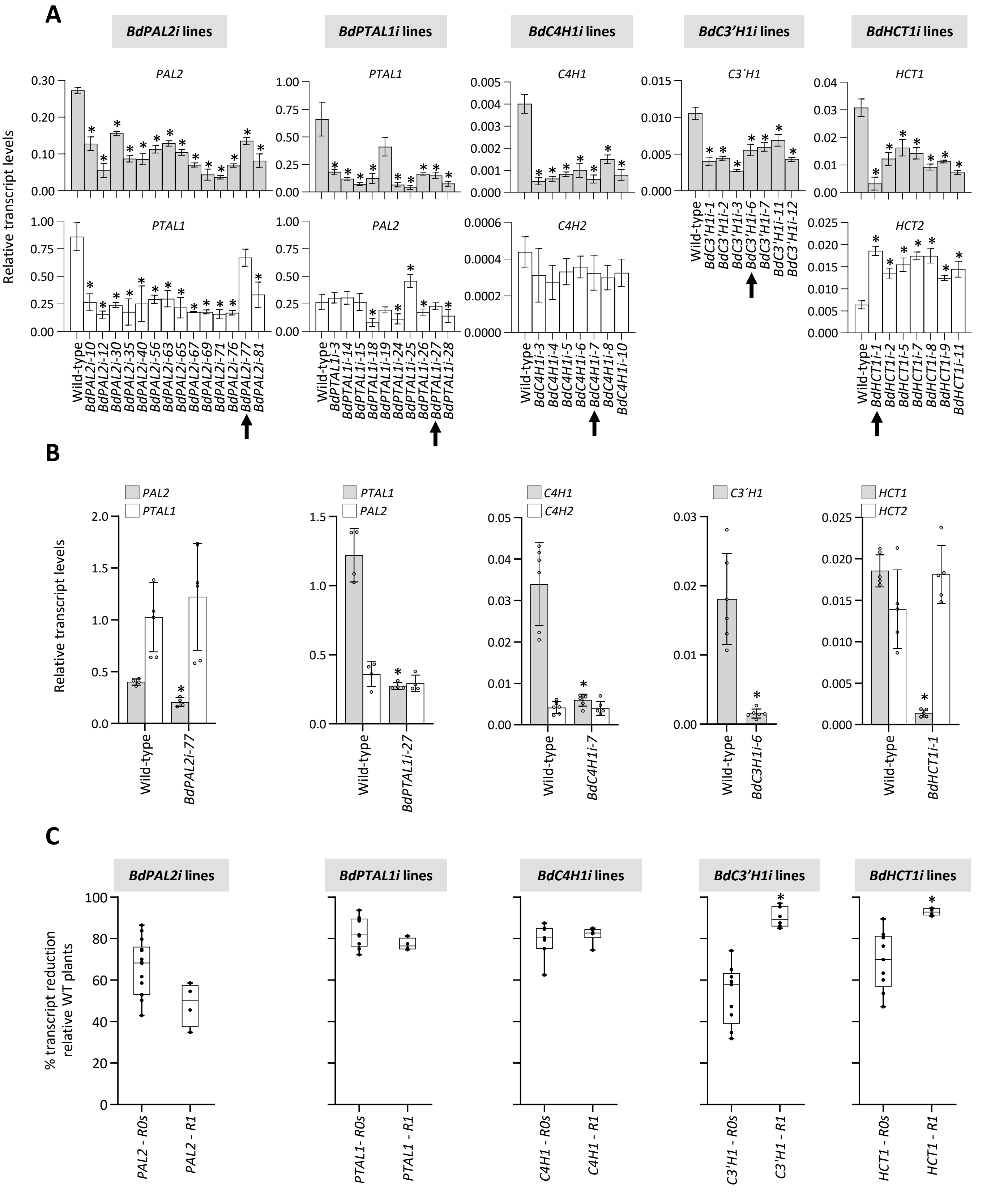

Supplemental Figure S3. qRT-PCR selection of Brachypodium RNAi lines used in this study (Supports Figure 1). Multiple R0 lines were generated for each construct from transformed callus. Seeds from the R0 lines with minimal off-target effects were harvested and the R1 generation was grown in soil under greenhouse conditions. **(A)** qRT-PCR selection of R0 generation lines. The parental lines for the R1 generation are indicated with an arrow. **(B)** Transcript levels of target genes in the selected R1 generation lines. **(C)** Reduction in relative expression of target genes in several R0 lines and selected R1 generation Brachypodium RNAi lines. Among all monofunctional PALs only *PAL2* showed significant transcript levels in the stems. Error bars in bar plots indicate ± SDs (n = 3). Box plots indicate the median (center lines), interquartile range (hinges) and whiskers represents min and max values. Data points for all biological replicates are shown. Asterisks denote significant differences compared to control groups ($P < 0.05$, two-sided unpaired *t*-test). Stem internodes 3–5 were harvested from plants at 45 days after germination and used for RNA extraction and real-time qRT-PCR analyses.

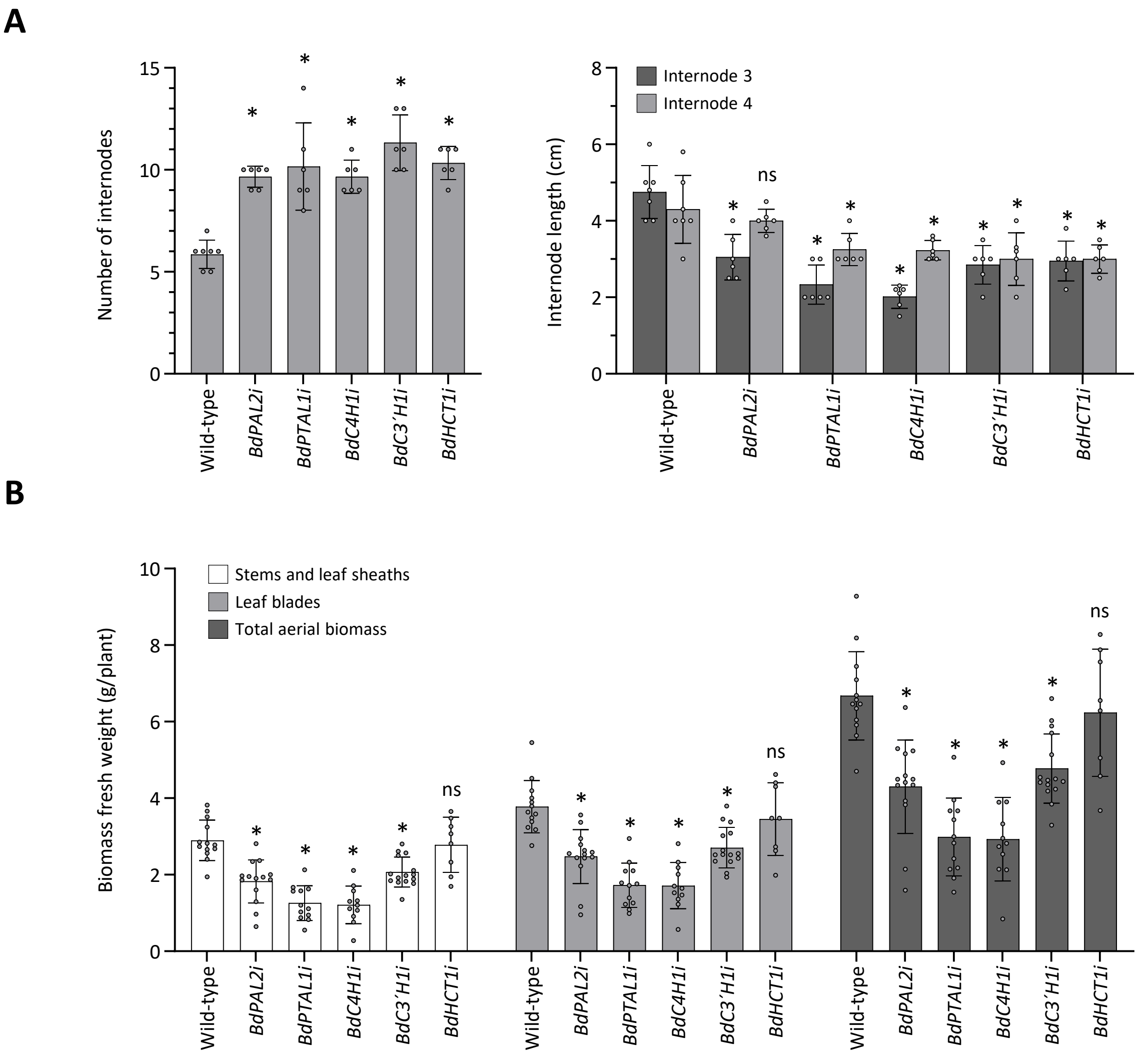

Supplemental Figure S4. Phenotypic effects of lignin modification in Brachypodium RNAi lines (Supports Figure 1). (**A**) Number and length of internodes. (**B**) Above-ground biomass. Error bars indicate ± SDs (n = 6 in panel A and n > 8 in panel B). Asterisks denote significant differences compared to control plants ($P < 0.05$, one-way ANOVA with post-hoc Dunnett's test). ns, denotes not significant differences.

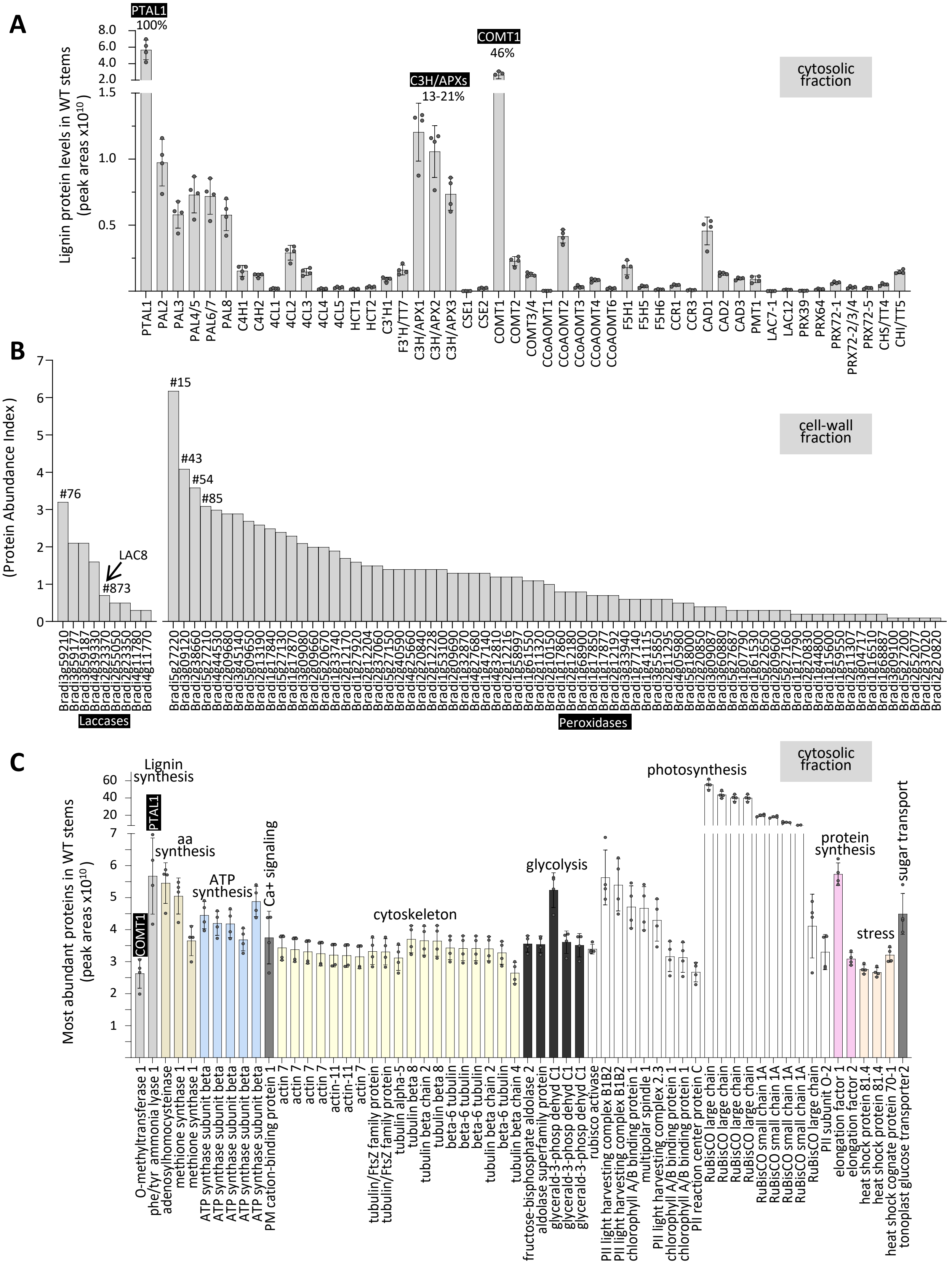

Supplemental Figure S5. Protein levels in mature stems from WT Brachypodium (Supports Figure 3). (**A**) Peak area intensities of 48 lignin and early flavonoid pathway enzymes. Percentages are relative proportions to the most abundant lignin enzyme PTAL1. Identical peptides and subsequent protein intensities were identified for PAL4/5, PAL6/7, COMT3/4 and PRX74-2/3/4 protein homologs (Supplemental Data Set S1). Independent replicated data for all the RNAi lines is provided in Supplemental Data Set S2. (**B**) Abundance levels of cell wall laccases and peroxidases in WT Brachypodium stem internodes as reported by Douché et al. (2021). Units are Protein Abundance Index, as defined by Rappsilber et al. 2002. Rank numbers (#) of protein abundance in the whole dataset (2116 proteins) are indicated. Lignin-related laccase 8 (Bradi2g23370, LAC8), recently characterized by Le Bris et al. (2019), is highlighted. (**C**) Top 60 most abundant proteins out of a total of 11,417 proteins identified. The slash between numbers separates homologs identified with identical protein peptides. Note that both caffeoyl shikimate esterase (CSE) proteins (Bradi4g45360 and Bradi2G12020) were only 50% similar to their Arabidopsis ortholog (AT1g52760). Error bars indicate ± SDs (n = 4). Data points for all biological replicates are shown.

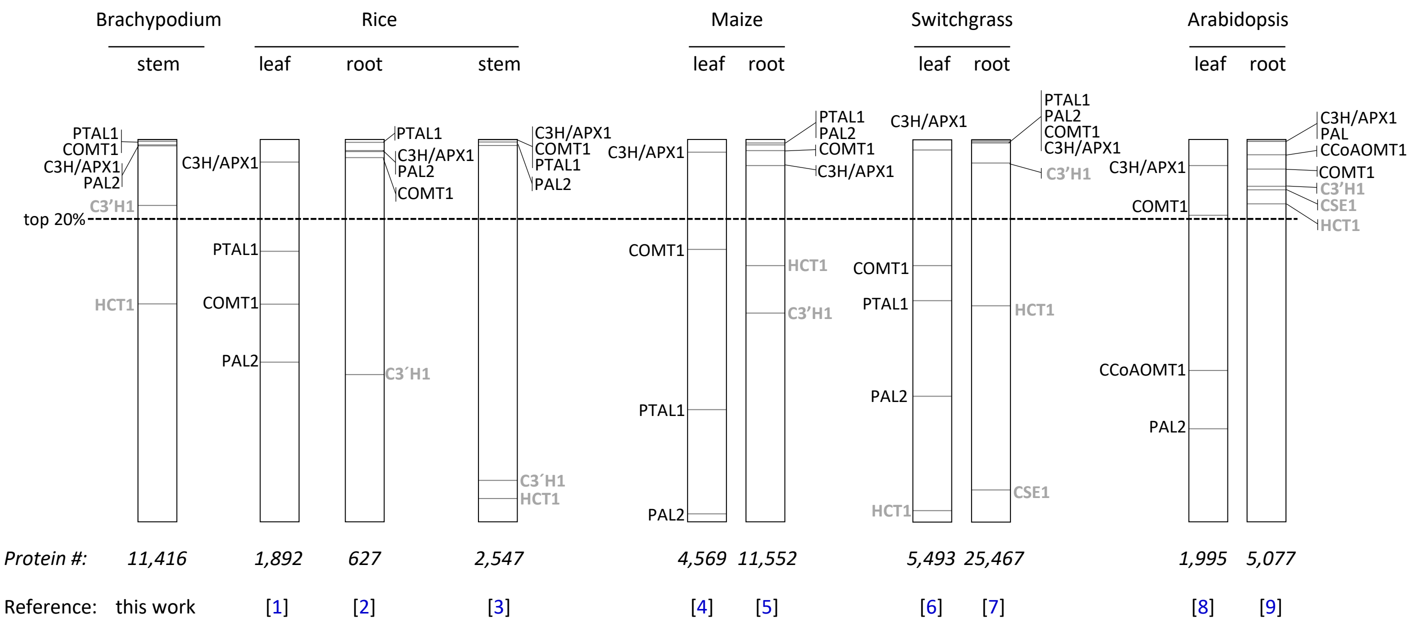

Supplemental Figure S6. Abundance levels of several early lignin pathway enzymes in different plant tissues and species obtained from this work and previous studies (Supports Figure 3). Proteins are ranked by abundance. The discontinuous line indicates the top 20 % most abundant proteins. The central enzymes of the shikimate shunt or esters route (HCT, C3'H and CSE) are shown in bold grey letters. Protein # is the total number of proteins identified in each plant species/tissue. References: Wu et al., 2016 [1]; Wang et al., 2014 [2]; Lin et al., 2017 [3]; Feng et al., 2017 [4]; Marcon et al., 2015 [5]; Ye et al., 2016 [6]; Qiao et al., 2021 [7]; Miller et al., 2017 [8]; Li et al., 2016 [9]. Gene IDs: Brachypodium PTAL1 (Bradi3g49250), PAL2 (Bradi3g49260), COMT1 (Bradi3g16530), C3H/APX1 (Bradi1g65820), HCT1 (Bradi5G14720), and C3'H1 (Bradi2G21300); Rice PTAL1 (Os02g41630), PAL2 (Os04g43760), COMT1 (Os08g06100), C3H/APX1 (Os03g17690), HCT1 (Os04g42250), and C3'H1 (Os05g41440), and CSE1 (Os02g11720); Maize PTAL1 (GRMZM2G074604), PAL2 (GRMZM2G441347), COMT1 (AC196475), C3H/APX1 (GRMZM2G137839), HCT1 (GRMZM2G051005) and C3'H1 (GRMZM2G138074); Switchgrass PTAL1 (Pavir.1NG356200), PAL2 (Pavir.7NG355500), COMT1 (Pavir.6KG071170), C3H/APX1 (Pavir.2NG612001), HCT1 (Pavir.1KG378900), C3'H (Pavir.3KG235800), and CSE1 (Pavir.1KG122700); Arabidopsis PAL (AT2G37040), COMT1 (AT5G54160), CCoAOMT1 (AT4G34050), C3H/APX (AT1G07890), HCT1 (AT5G48930), C3'H1 (AT2G40890) and CSE1 (AT1G52760). PTAL1 is not present in Arabidopsis; HCT1 was not detected in rice (both leaf and root tissues), and in maize and Arabidopsis leaf tissues. CSE1 is not present in Brachypodium and maize, and it was not detected in any tissue in rice or in switchgrass and Arabidopsis leaves. Full dataset provided in Supplemental Data Set S4.

Primary metabolites (GC-MS)

1. sucrose
2. citric acid
3. malic acid
4. 5-oxo-proline
5. glucose
6. glutamine
7. asparagine
8. ethanolamine
9. aspartic acid
10. galactose
11. serine
12. alanine
13. B-sitosterol
14. phytol
15. N-metabolite 1
16. fructose
17. valine
18. N-metabolite 2
19. aconitic acid
20. raffinose
21. campesterol
22. threonic acid
23. threonine
24. allantoin
25. shikimic acid
26. glycine
27. fumaric acid
28. S-lignan glycoside
29. glycerol-1/3-phosphate
30. 5-hydroxytryptamine
31. alpha-linolenic acid
32. palmitic acid
33. glutamic acid
34. glycerol
35. G-lignan.1
36. N-metabolite 3
37. N-metabolite 4
38. N-metabolite 5
39. quinic acid
40. succinic acid
41. N-metabolite 6
42. a-ketoglutaric acid
43. glyceric acid
44. 1-hexacosanol
45. salicylic acid 2-O-glucoside
46. isoleucine
47. leucine
48. tyrosine
49. glycolic acid
50. stigmastanol
51. myo-inositol
52. benzylglucuronate
53. lysine
54. oxalic acid
55. adenosine
56. phenylalanine
57. monopalmitin
58. 3-hydroxyproline
59. stigmasterol
60. maleic acid
61. monostearin
62. tetracosanoic acid
63. fucosterol
64. N-metabolite 7
65. citraconic acid
66. digalactosylglycerol
67. glucose 6-phosphate
68. salicylic acid
69. gentisic acid-5-O-glucoside
70. phosphoethanolamine
71. glucuronic acid conjugate
72. guanosine
73. monogalactosylglycerol
74. 1,2,4-benzenetriol
75. fructose 6-phosphate
76. hexacosanoic acid
77. docosanoic acid
78. ascorbate conjugate
79. adenine
80. azelaic acid
81. hydroquinone
82. G lignan.2
83. *p*-coumaroyl shikimate
84. guanine

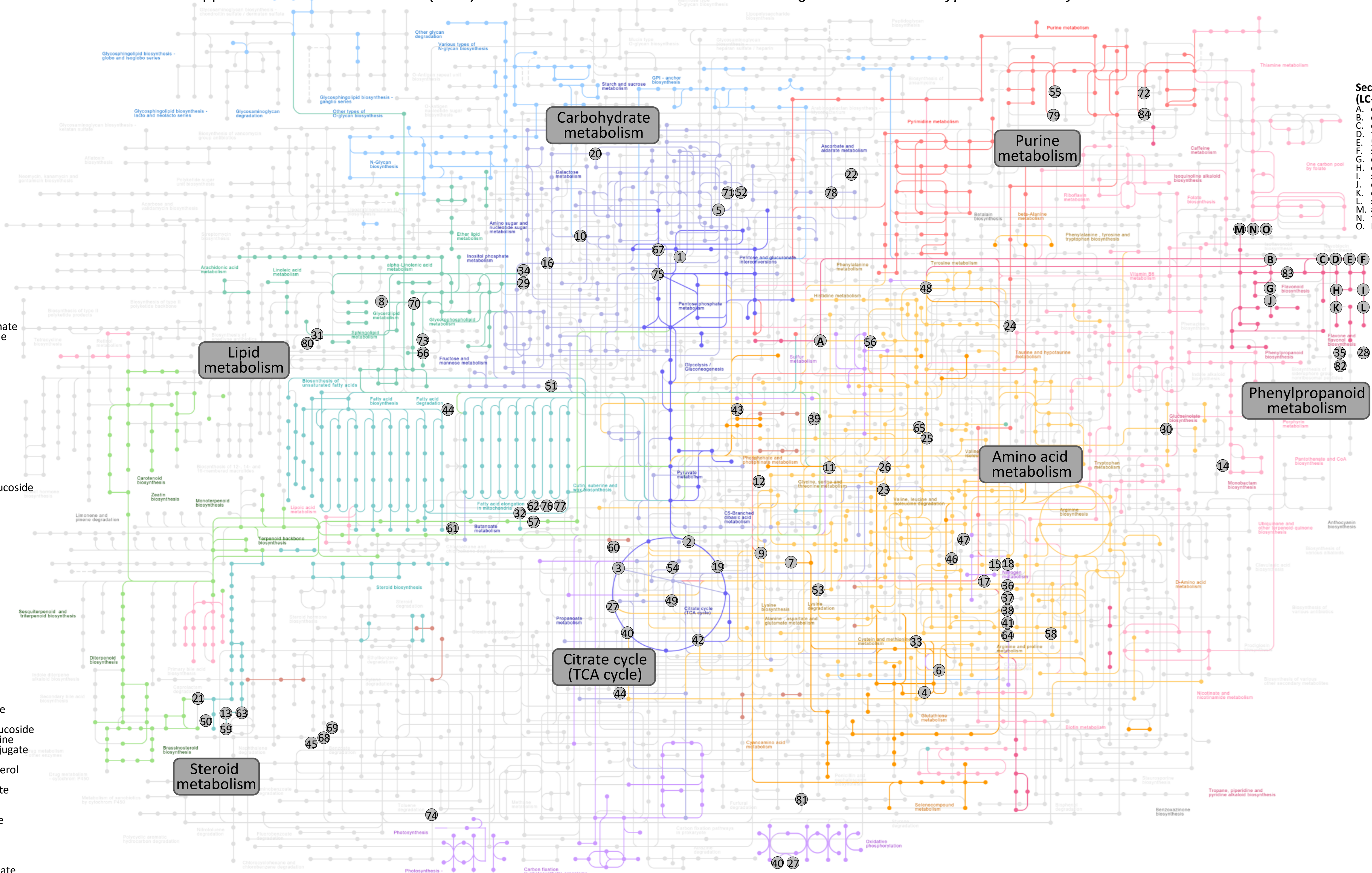

Secondary metabolites (LC-MS/MS)

A. cinnamate
B. coumarate
C. caffeate
D. ferulate
E. 5H-ferulate
F. sinapate
G. coumaraldehyde
H. coniferaldehyde
I. sinapaldehyde
J. coumaryl alcohol
K. coniferyl alcohol
L. sinapyl alcohol
M. apigenin
N. tricin
O. Isovitexin

Supplemental Figure S7 (Supports Figure 4). Metabolic KEGG maps overlaid with primary and secondary metabolites identified in this work.

BdPAL2i lines

RNAi target: *PAL2* (Bradi3G49260, I1IBR6)

Primary metabolites (GC-MS)

UPREGULATED:
3. malic acid
4. 5-oxo-proline
6. glutamine
7. asparagine
8. ethanolamine
9. aspartic acid
11. serine
12. alanine
14. phytol
15. N-metabolite.1
22. threonic acid
23. threonine
25. shikimic acid
29. glycerol-1/3-phosphate
30. serotonin
31. alpha-linolenic acid
33. glutamic acid
34. glycerol
36. N-metabolite. 3
37. N-metabolite. 4
38. N-metabolite.5
39. quinic acid
40. succinic acid
41. N-metabolite.6
42. alpha-ketoglutaric acid
43. glyceric acid
48. tyrosine
55. adenosine
56. phenylalanine
58. 3-hydroxyproline
63. fucosterol
70. phosphoethanolamine
80. azelaic acid
84. guanine

DOWNREGULATED:
10. galactose
17. valine
46. isoleucine
47. leucine
49. glycolic acid
65. citraconic acid
71. glucuronate conjugate
74. 1,2,4-benzenetriol
82. **G**-lignan

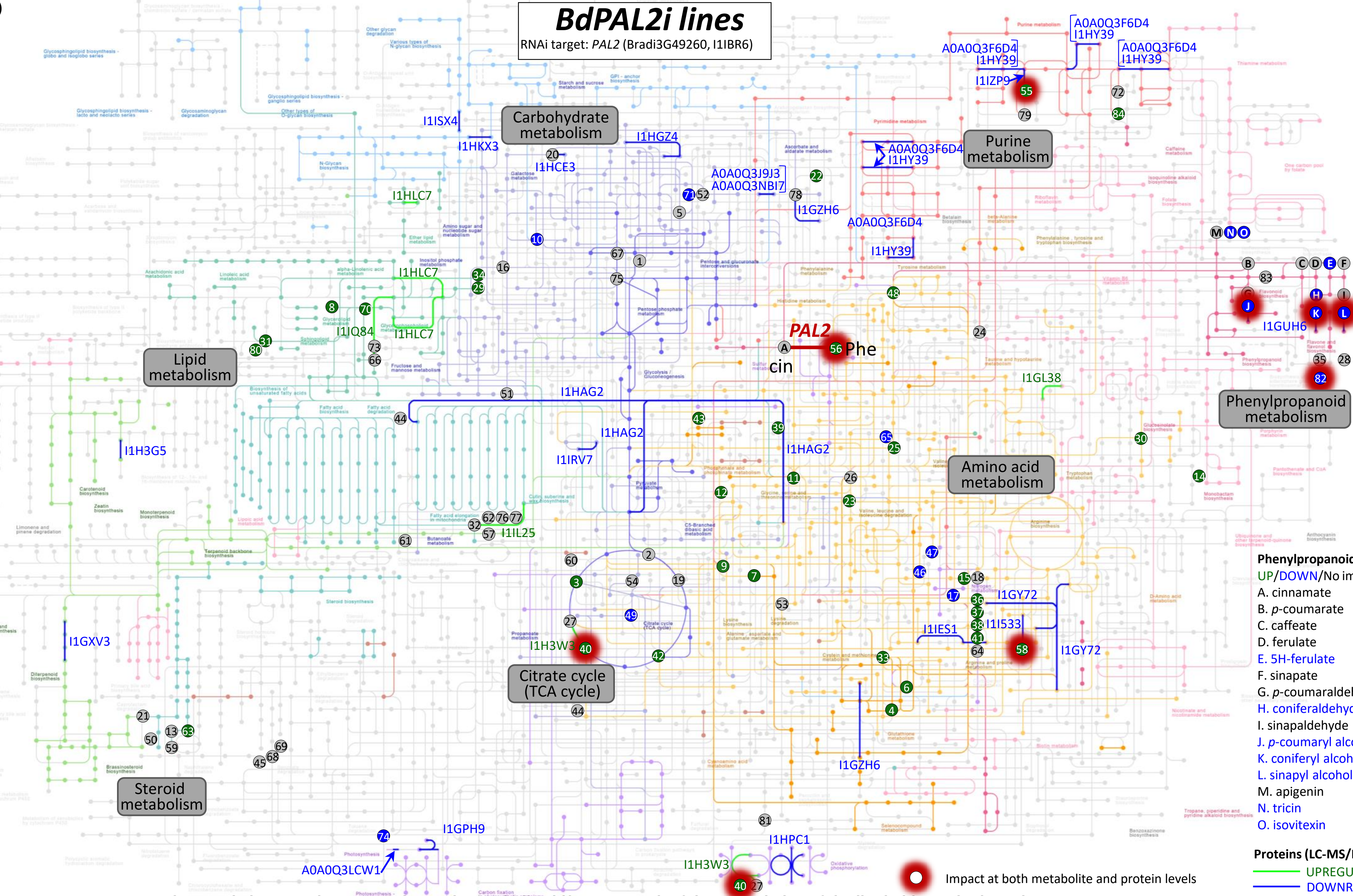

Phenylpropanoids (LC-MS)

UP/DOWN/No impact
A. cinnamate
B. *p*-coumarate
C. caffeate
D. ferulate
E. 5H-ferulate
F. sinapate
G. *p*-coumaraldehyde
H. coniferaldehyde
I. sinapaldehyde
J. *p*-coumaryl alcohol
K. coniferyl alcohol
L. sinapyl alcohol
M. apigenin
N. tricin
O. isovitexin

Proteins (LC-MS/MS)

UPREGULATED
DOWNREGULATED

Supplemental Figure S8 (Supports Figure 4). Metabolic shifts as a result of downregulation of the lignin biosynthetic pathway gene *PAL2*.

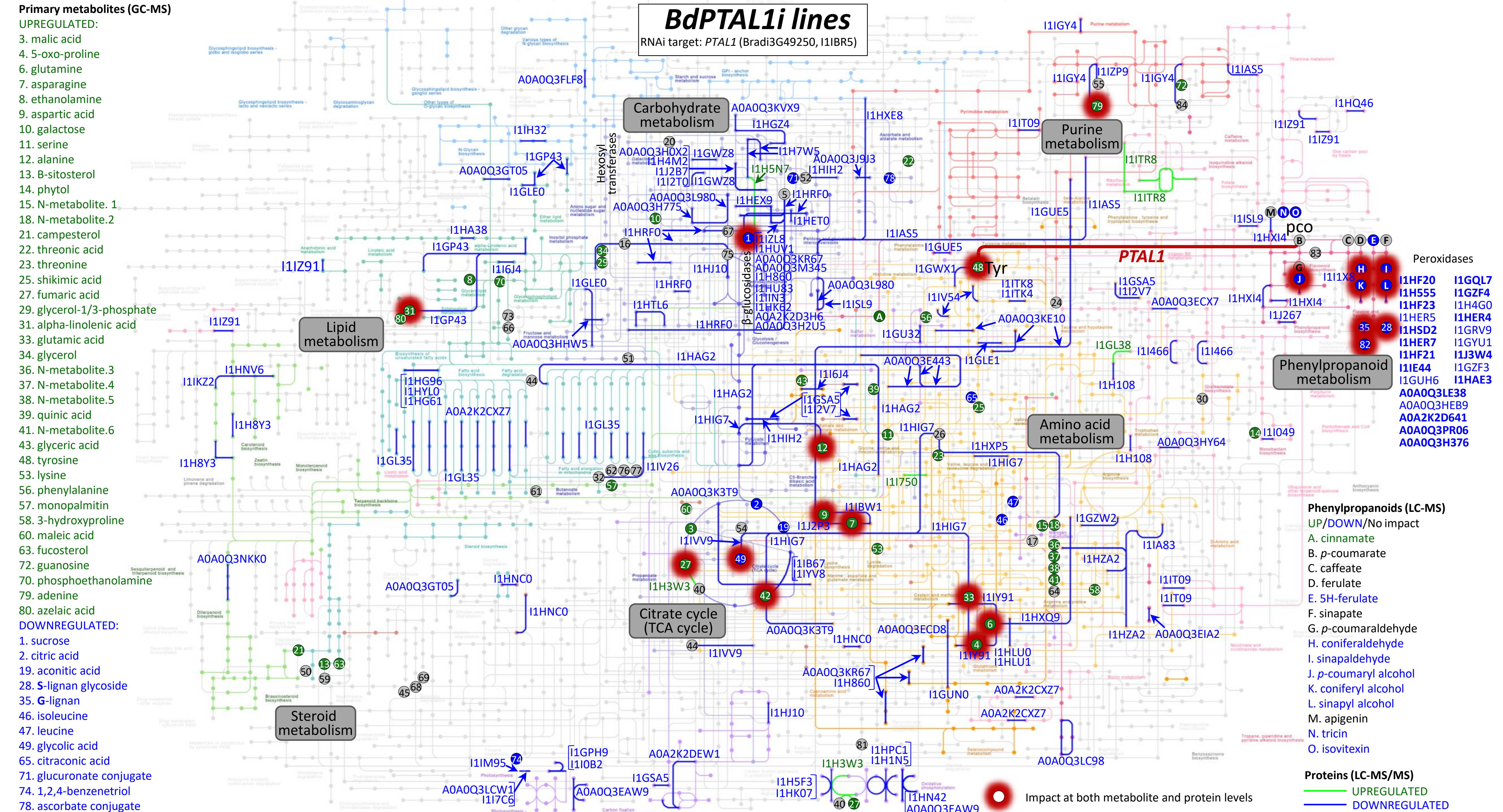

Supplemental Figure S9 (Supports Figure 4). Metabolic shifts as a result of downregulation of the lignin biosynthetic pathway gene *PTAL1*.

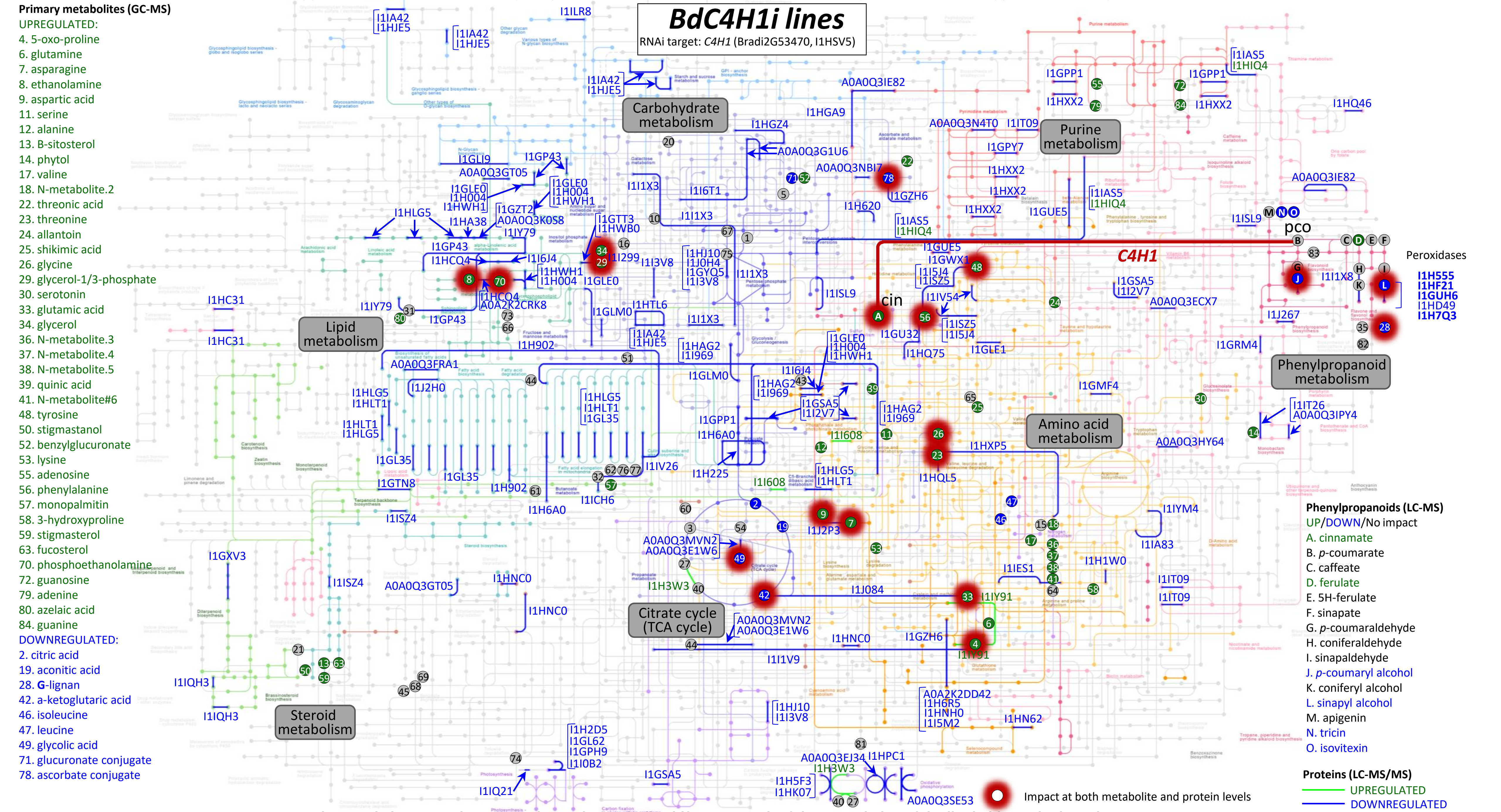

Supplemental Figure S10 (Supports Figure 4). Metabolic shifts as a result of downregulation of the lignin biosynthetic pathway gene *C4H1*.

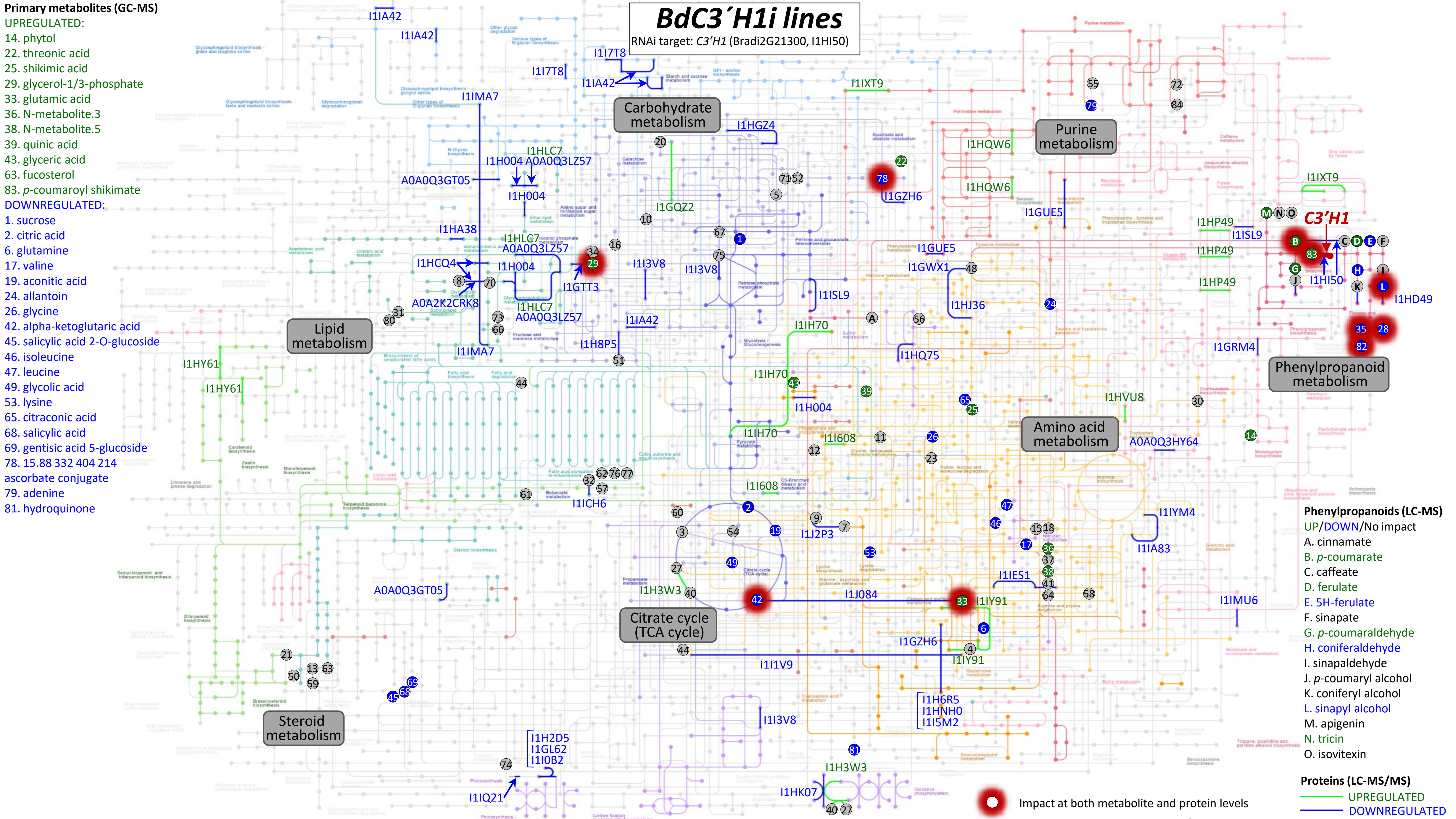

Supplemental Figure S11 (Supports Figure 4). Metabolic shifts as a result of downregulation of the lignin biosynthetic pathway gene *C3´H1*.

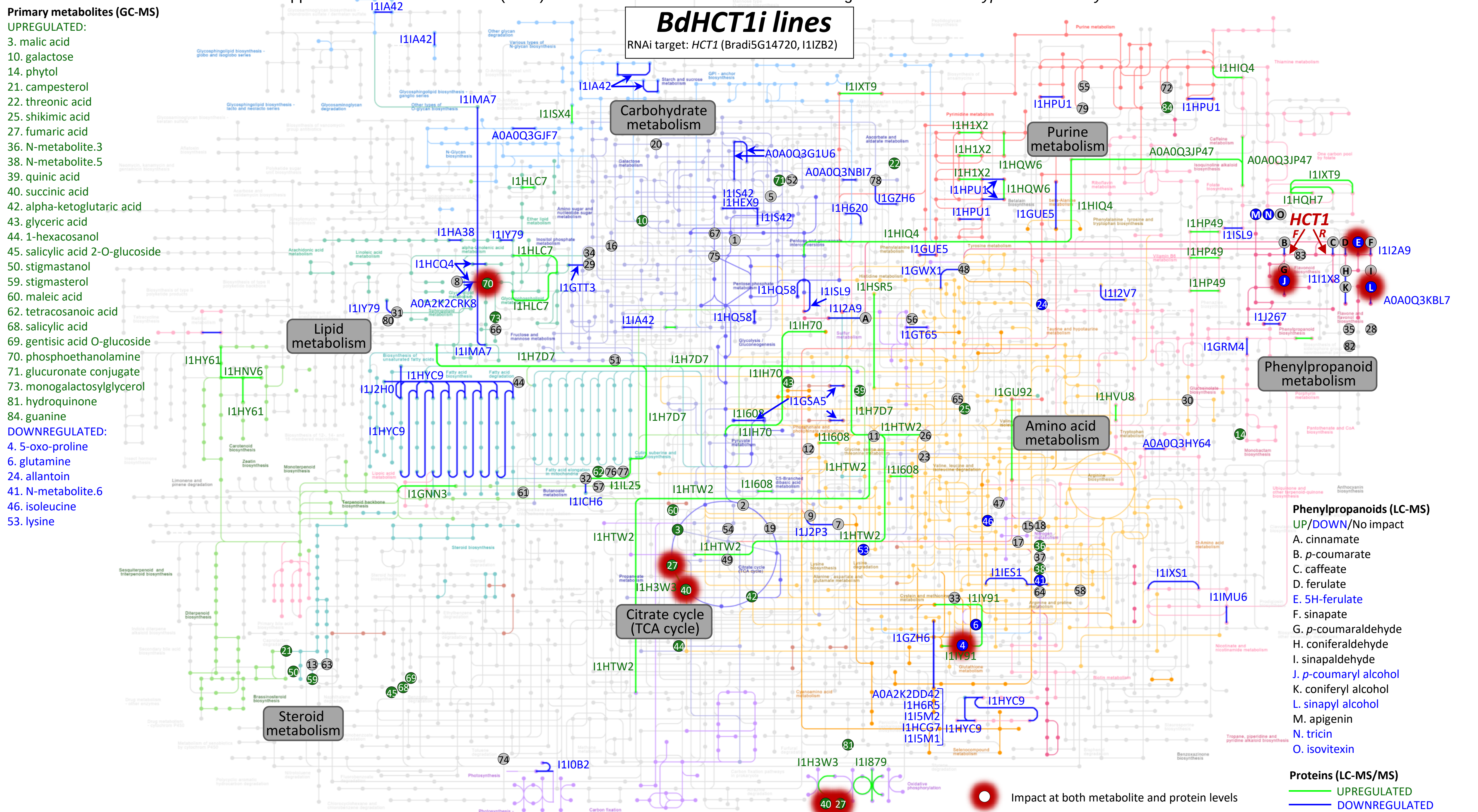

Supplemental Figure S12 (Supports Figure 4). Metabolic shifts as a result of downregulation of the lignin biosynthetic pathway gene *HCT1*.

Supplemental Data. Barros et al. (2022).
Proteomic and metabolic disturbances in lignin-modified *Brachypodium distachyon*. Plant Cell.

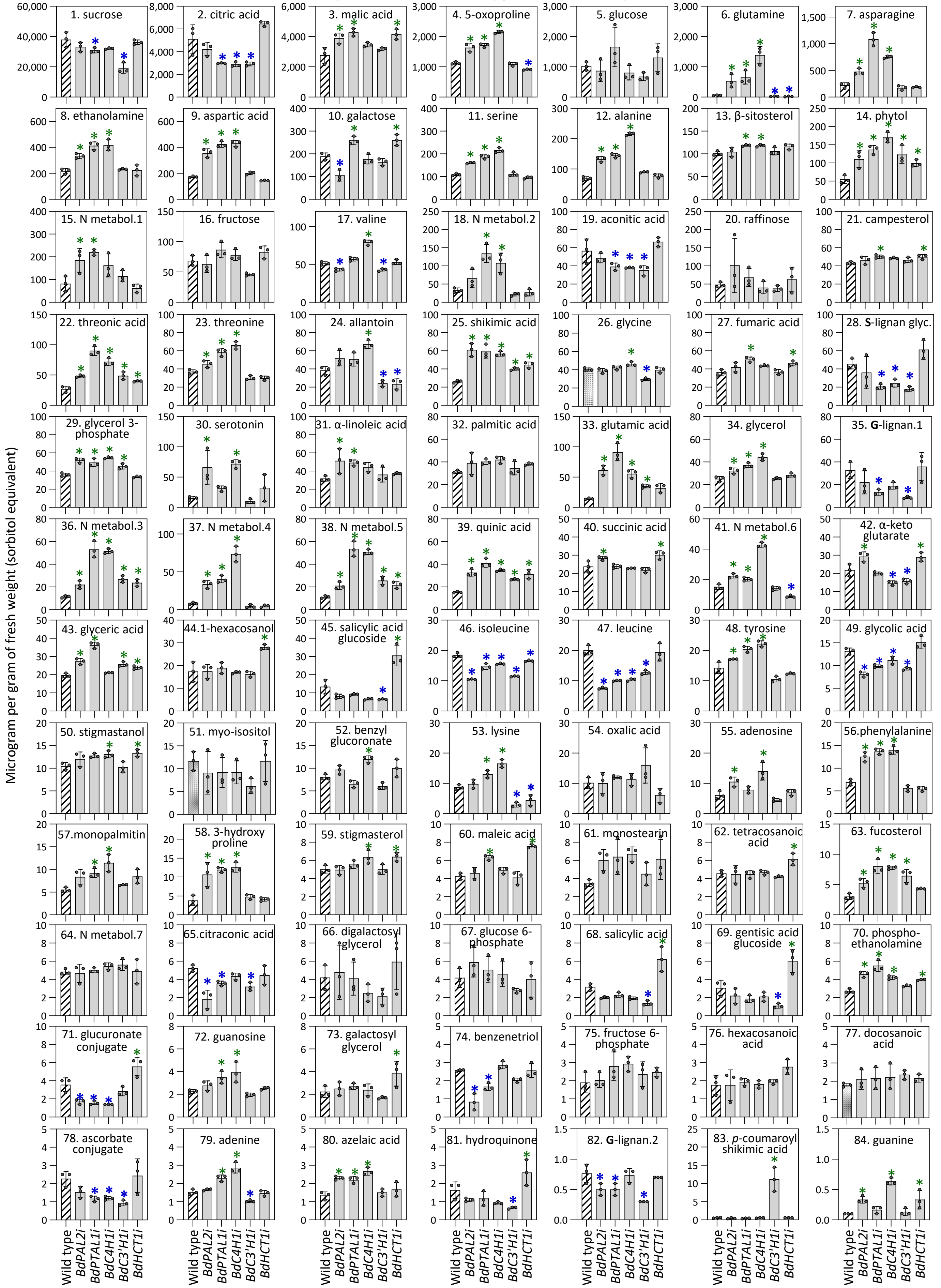

Supplemental Figure S13. Metabolite levels in mature stem tissues of Brachypodium RNAi lines (Supports Figure 4). Concentrations of soluble metabolites in µg/g dry weight as sorbitol equivalents in mature stem internodes harvested from plants at 30 days after germination and measured by GC-MS. Numbers (1-84) assigned based on average abundance. Error bars indicate ± SDs (n = 4). Green and blue asterisks indicate significantly up- and down-regulated metabolites, respectively ($P < 0.05$, one-way ANOVA with post-hoc Dunnett's test).

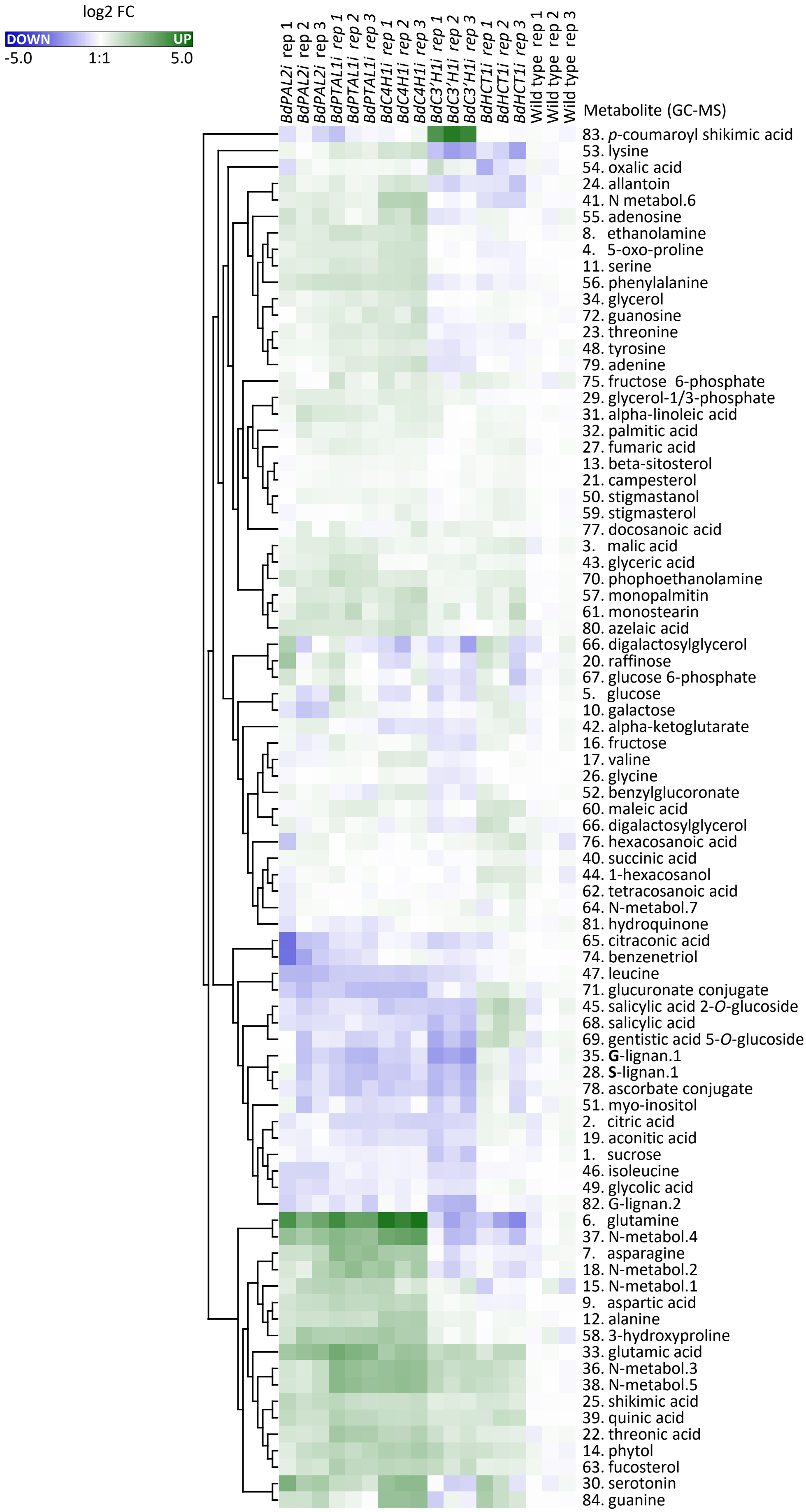

Supplemental Figure S14. Heatmap of metabolite levels in mature stem tissues of Brachypodium RNAi lines (Supports Figure 4). Heatmap displays log2 fold-changes (FC) in abundance of untargeted metabolites measured by GC-MS in RNAi-mediated lignin gene silenced lines. Numbers (1-84) assigned based on average abundance. Each square represents an independent biological replicates.

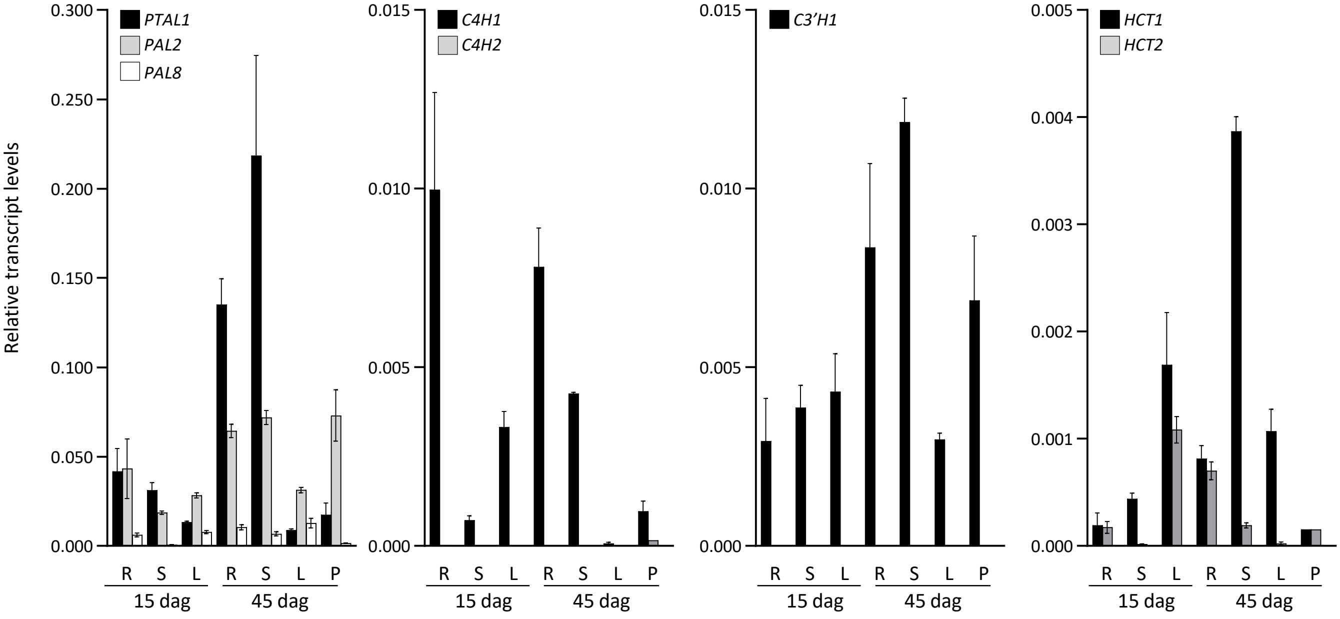

Supplemental Figure S15. Organ-specific expression of monolignol pathway genes in Brachypodium wild-type plants (Supports Figure 4). Tissues analyzed were roots (R), stems (S), leaves (L) and panicles (P) at 15 and 45 days after germination (dag). Among all PALs only *PTAL1*, *PAL2* and *PAL8* showed significant expression in the tissues analyzed. Transcript levels were measured by qPCR, and are expressed relative to the housekeeping gene Brachypodium tubulin (Bradi1g10150). Error bars indicate ± SDs (n = 3).

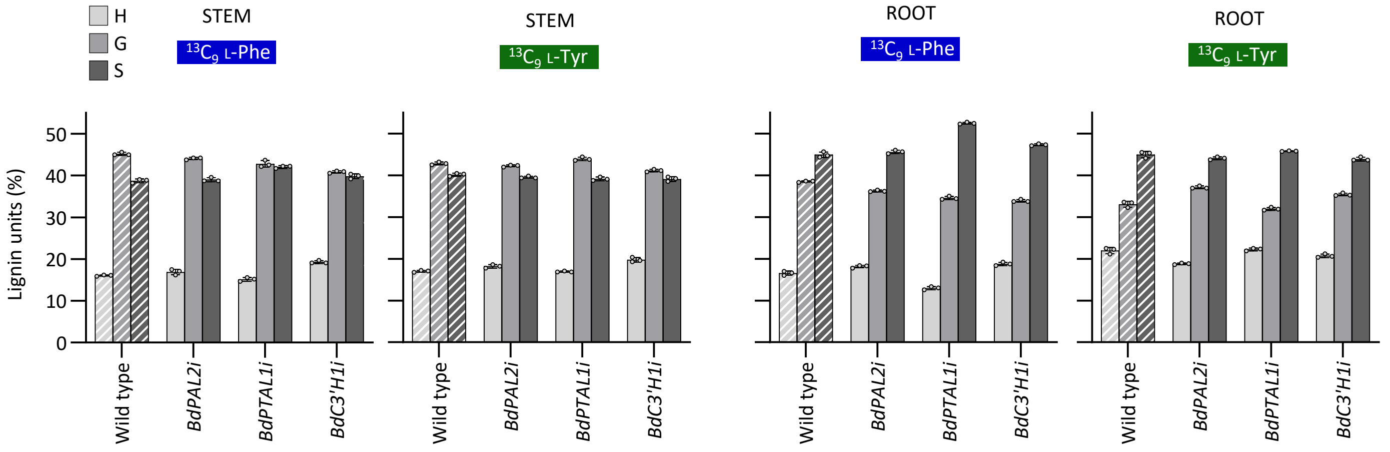

Supplemental Figure S16. Lignin composition of in vitro grown Brachypodium RNAi lines fed with ^{13}C-labeled precursors (Supports Figure 5). Lignin composition is given as percentage of lignin subunits extracted from root and stem internodes harvested from plants grown in vitro in culture media administrated with ^{13}C-Phe or ^{13}C-Tyr as indicated. Ten to fifteen plants were pooled together for each treatment and genotype, cell wall residues were extracted and lignin-derived monomers measured after thioacidolysis using GC-MS. Error bars indicate ± SDs (n = 3).

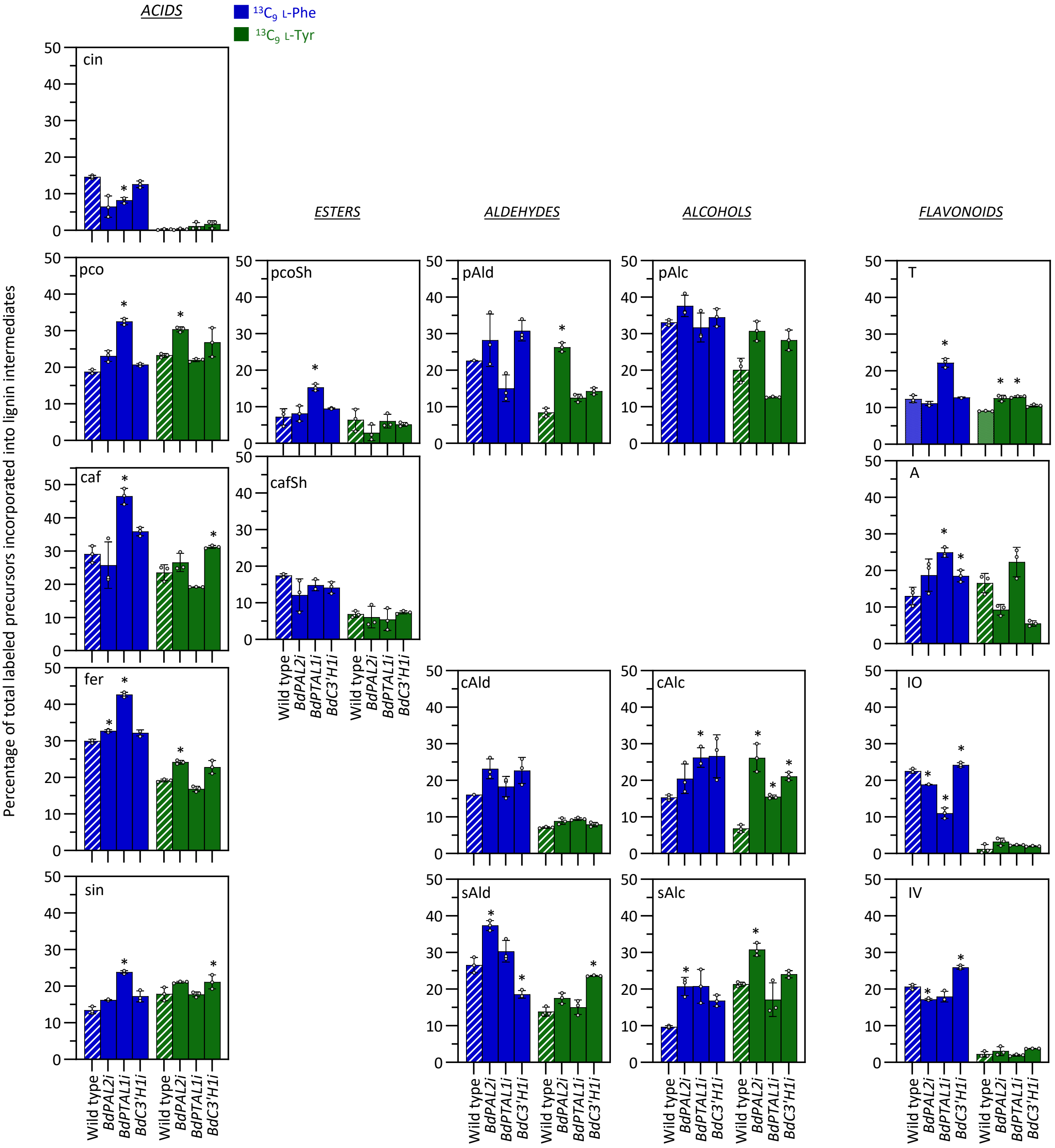

Supplemental Figure S17. Proportion of $^{13}C_9$-Phe (blue) and $^{13}C_9$-Tyr (green) labeled lignin intermediates in roots of Brachypodium RNAi lines (Supports Figure 5). Concentration of soluble metabolites (nmol/g dry weight) in mature stem internodes harvested from plants at 30 days after germination and measured by LC-MS/MS. Error bars indicate ± SDs (n = 3). Asterisks indicate significant differences (*P* < 0.05, two-way ANOVA with post-hoc Dunnett's test). Abbreviations: cin, cinnamate; pco, *p*-coumarate; caf, caffeate; fer, ferulate; sin, sinapate; pcoSh, *p*-coumaroyl shikimate; cafSh, caffeoyl shikimate; pAld, *p*-coumaraldehyde; cAld, coniferaldehyde; sAld, sinapaldehyde; pAlc, *p*-coumaryl alcohol; cAlc, coniferyl alcohol; sAlc, sinapyl alcohol, T, tricin; A, apigenin; IO, isoorientin; IV, isovitexin.

	Primer name	Primer sequence	Region length (bp)
A	PAL_RNAi-F	5’-GACTGGGTCATGAACAGCATG-3’	287
	PAL_RNAi-R	5’-GTGACATTGGCATTGAGCAGC-3’	
	PTAL_RNAi-F	5’-GAGCCATCTCGGAGAAGGA-3’	371
	PTAL_RNAi-R	5’-GATCCCGGCGTTCAGATG-3’	
	C4H_RNAi-F	5’-GGCAACTGGCTGCAGGT-3’	249
	C4H_RNAi-R	5’-GCCAGTGGTCGCCGTAC-3’	
	C3'H_RNAi-F	5’-TGAAGGAAAGCCATTGACAGTGAGGA-3’	272
	C3'H_RNAi-R	5’-TGATCTTCTTGGTTAGCCGGTCCC-5’	
	HCT1_RNAi-F	5’-TGGATTACCTGGAGCTGCA-3’	259
	HCT1_RNAi-R	5’-TTCCGGAACTTCTCCATGTGC-3’	
	HCT2_RNAi-F	5’-GACTCGGAGCCGCCGCAGGCCA-3’	360
	HCT2_RNAi-R	5’-CGTGTTCGCGCCGTCCGCCAGCG-3’	
B	PTAL1_F	5’-GCTGCGTGTACCTCACCG-3’	177
	PTAL1_R	5’-CGATGGATACGGGCCAATGGA-3’	
	PAL2_F	5’-GCACGGAGTACCTGACAGGG-3’	158
	PAL2_R	5’-TCTTCAGCTCGATCAGCACAGTG-3’	
	C4H1_F	5’-TGCAGCACACACACAGAAACACC-3’	118
	C4H1_R	5’-CGGAACCGCTTGCCCGT-3’	
	C4H2_F	5’-GTACAGTGTCCGTTCCCGCC-3’	136
	C4H2_R	5’-GGCAGCTTCAGCTTGCGG-3’	
	C3'H_F	5’-ACCTCCCATACCTGCTAGCTGT-3’	152
	C3'H_R	5’-GACCGCCCAAACATTCACCATGA-5’	
	HCT1_F	5’-TCGCAGCCTTCCTCCTGAA-3’	163
	HCT1_R	5’-GGCTCCATCTTCTAACCCACTGG-3’	
	TUB_F	5’-GCCTTTGTCCACTGGTATGT-3’	110
	TUB_F	5’-AACTCTGCACCAACCTCTTC-3’	
C	Hyg_F	5’-CGAAATTGCCGTCAACCAAGCTCT-3’	414
	Hyg_R	5’-CGACGTCTGTCGAGAAGTTT-3’	

Supplemental Table S1. Oligonucleotides used in this work. Section (A), primers for RNAi-targeted suppression; (B), primers for RT-qPCR. (C), primers for transgene integration analysis.

Compound	^{12}C- and ^{13}C-parent/daughter transitions (m/z)	MS parameters from MRM acquisition				Electrospray ionization source parameters (+ polarity)					
		DP (V)	EP (V)	CE (V)	CXP (V)	CG (psi)	ISV (V)	T (ºC)	CAD (psi)	GS1 (psi)	GS2 (psi)
cinnamate	149/103; 158/111	20	10	25	12	40	5000	550	Medium	50	60
p-coumarate	165/119; 174/127	20	10	25	14	40	5000	550	Medium	50	60
p-coumaraldehyde	147/119; 156/127	-50	-10	-24	-13	40	5000	550	Medium	50	60
p-coumaroyl alcohol	149/131; 158/140	-25	-10	-14	-13	40	5000	550	Medium	50	60
p-coumaroyl shikimate	319/163; 328/172	-50	-10	-20	-9	40	5000	550	Medium	50	60
caffeoyl shikimate	335/179; 344/188	-70	-10	-22	-9	40	5000	550	Medium	50	60
caffeate	179/135; 188/143	-35	-10	-20	-15	40	5000	550	Medium	50	60
ferulate	193/178; 202/187	-25	-10	-16	-7	40	5000	550	Medium	50	60
sinapate	225/175; 234/184	20	10	19	10	40	5000	550	Medium	50	60
coniferaldehyde	179/147; 188/156	30	10	17	10	40	5000	550	Medium	50	60
coniferyl alcohol	163/131; 172/140	45	10	13	12	40	5000	550	Medium	50	60
sinapaldehyde	209/177; 218/186	30	10	15	10	40	5000	550	Medium	50	60
sinapyl alcohol	209/194; 218/203	-30	-10	-16	-11	40	5000	550	Medium	50	60
tricin	331/315; 340/324	100	10	41	34	40	5000	550	Medium	50	60
apigenin	269/117; 278/125	-90	-10	-42	-13	40	5000	550	Medium	50	60
isovitexin	433/283; 442/292	80	10	35	14	40	5000	550	Medium	50	60
isoorientin	449/299; 458/308	50	10	39	14	40	5000	550	Medium	50	60

Supplemental Table S2. LC/MS-MS parameters used for the identification of intermediate metabolites in the lignin biosynthetic pathway. MRM: Multiple Reaction Monitoring, DP: Declustering Potential, EP: Entrance Potential, CE: Collision Energy and CXP: Collision cell Exit Potential in Volts (V) are shown for each metabolite measured. CG: Curtain Gas, ISV: IonSpay Voltage, T: Temperature, CAD: Collision Activated Dissociation, GS1: Nebulizing Gas, GS2: Heating Gas.
