# Supplementary material for: Proteomic and metabolic disturbances in lignin-modified Brachypodium distachyon
Source: Plant Cell. 2022 Jun 7;34(9):3339–63. doi: 10.1093/plcell/koac171 (PMC9421481; doi:10.1093/plcell/koac171)
Supplement: koac171_Supplementary_Data [file tpc22.00295.PeerReview.pdf › tpc22.00295.PeerReview.pdf]

Proteomic and Metabolic Disturbances in Lignin-Modified *Brachypodium distachyon*

Jaime Barros, Him K. Shrestha, Juan C. Serrani-Yarce, Nancy Engle, Paul E. Abraham, Timothy J. Tschaplinski, Robert L. Hettich, and Richard A. Dixon

Corresponding authors: Jaime Barros Jaime.Barros@missouri.edu; *Richard A. Dixon* Richard.Dixon@unt.edu

Review timeline:

**TPC2022-RA-00295**	Submission received:	March 28, 2022
	1st Decision:	May 5, 2022 *accept with minor revision*
**TPC2022-RA-00295R1**	1st Revision received:	May 20, 2022
	2nd Decision:	May 23, 2022 *acceptance pending, sent to science editor*
	Final acceptance:	June 2, 2022

REPORT: (The report shows the major requests for revision and author responses. Minor comments for revision and miscellaneous correspondence are not included. The original format may not be reflected in this compilation, but the reviewer comments and author responses are not edited, except to correct minor typographical or spelling errors that could be a source of ambiguity.)

TPC2022-RA-00295 1st Editorial decision – *accept with minor revision* **May 5, 2022**

The reviewers and editors involved in evaluation all greatly appreciated the work you have done and the discoveries made on the lignin pathway(s) in Brachypodium and don't suggest further experiments are required for an acceptable manuscript. This represents a substantial advance for the field. However, they do think that you could have done a better job relating your findings with those of other lignin-related studies in Brachypodium and in other plants, as outlined by them. Also, better descriptions and details in some areas, as indicated in the reviews, would improve the manuscript. Other smaller corrections and clarifications are sought to improve the documentation and the discussion. We look forward to your revised version.

--- Reviewer comments:
Reviewer #1 (Comments for the Author):
Summary:
The authors build on their previous studies related to the flux of Phe and Tyr precursors to building lignin in plants. Here they generate an informative set of RNAi lines which exhibit reduced expression in early steps of the phenylpropanoid pathway. Besides presenting a rich dataset of metabolic perturbances in these RNAi lines, the interpretation suggests a link between primary and secondary metabolism via glutamine. Ammonia lysases are revealed to be a major enzyme component of plant cells undergoing lignification. A particular novelty of the study is the indication that Brachypodium can make syringyl lignin only using Tyr, and we do not yet know all the enzymes involved. Comments are provided to help improve figures and tables and to consider their findings in the light of some other recent publications.

Major and Minor Comments
Introduction:
The Introduction provides a useful summary of how the main enzymatic steps in the phenylpropanoid pathway have been discovered as well as current differences between dicots and the grasses. These differences provide the rationale for pursuing this work, which involves genetic (RNAi), proteomic, and 13C-isotopic labeling to explore this pathway more comprehensively in Brachypodium.

Results:
Fig 1 Good - clear phenotypes showing weaker stems of RNAi lines - but I would have found it helpful to have a small photo (or supplemental figure) showing the internode locations that are collected and the stage of growth for the lignin analysis in Fig 1b.(not an essential addition but helpful to a reader not very familiar with Brachypodium).
It is good that they selected RNAi lines which mostly affected the target gene and not related genes (Supp Fug 3),

however I do wonder for the larger gene families with duplicate genes such as the PAL cluster if other members were also affected - if they have data they on the expression of other PALs for example they should share that in the supplemental figure 3 (not essential however).

Fig 1B shows clear effects on lignin staining and lignin content and ratios (Fig 1B) This is complemented nicely by the lignin metabolite analysis later (Fig 5). Overall a suitable set of experimental lines to address the effects on metabolic flux.

Fig 2. Very useful to get an overview of where metabolites might be expected to accumulate in the various RNAi lines. Since the figure is already colored they could have made better use of color to differentially highlight the RNAi targets - and use these same colors in the X-axis of the graphs.

Table 1: OK - as data for other family members are provided in Supp Tables 1 and 2. However see comments in the Materials and Methods about providing gene model IDs from the genome of Brachypodium inbred line Bd21-3 and not the reference genome Bd21 since that it sit he line they used for measurements and the genome is available. If it makes the table too cluttered then provide these in a supplementary table.

Fig 3. Effect of RNAi on enzymes that are different from the targeted one for example - why are so many PALs at lower levels when only one is subject to RNAi?

The shaded boxes on the left column are very helpful to get an overview of what the graphs are telling and all family members are included.

Fig 4: As space permits they could add more identifiers to metabolites that are significantly increased or decreased as they did for glutamine for example - otherwise figure is hard to interpret without consulting the detailed supplementary tables 10-14. For Part B a small table below each graph could be used to indicate the function of proteins whose altered levels are of noted significance.

Fig 5: Lignin analysis. Graph shows the preferential incorporation of labelled Phe or Tyr into H, G or S lignin in WT vs three of the RNAi lines. Fig is OK and color coding blue, green and grey helps interpretation.

Fig 6: Model of RNAi impacts on lignin and growth -OK

Discussion:

Implications of the high abundance of PTAL1 - I was surprised that no-one has definitively identified a kinase that regulates PAL since evidence for PAL phosphorylation has been known since the early 1990 and even likely threonine residues as the target of phosphorylation. So the authors make a good point that this is an avenue for future exploration.

However, they might reference a recent review in this area from Sulis et al., 2021, and the possible role of a calmodulin-like domain protein kinase (CDPK) in this process (Allwood et al., 1999)

Sulis DB, Wang JP. Regulation of Lignin Biosynthesis by Post-translational Protein Modifications. Front Plant Sci. 2020 Jul 2;11:914. doi: 10.3389/fpls.2020.00914. PMID: 32714349; PMCID: PMC7343852.

Allwood EG, Davies DR, Gerrish C, Ellis BE, Bolwell GP. Phosphorylation of phenylalanine ammonia-lyase: evidence for a novel protein kinase and identification of the phosphorylated residue. FEBS Lett. 1999 Aug 20;457(1):47-52. doi: 10.1016/s0014-5793(99)00998-9. PMID: 10486561.

Crosstalk between Primary and Secondary Metabolism

I caution against the overinterpretation of metabolic perturbances due to RNAi interference - backups are expected and the ability of plant cells to maintain homeostasis pushed to the limit. However this area of study has value as scientists try to modify lignin content for increased biofuel production and may lead to ways in which the negative growth effects are minimized. In this respect the altered downregulation of enzymes in later genes in the PTAL line is of interest and value - but may also reflect regulation at the transcriptional level.

Link to SA metabolism.

They might include in their interpretation the recent observations by Kim et al. 2021 that exogenous ammonia can cause a reduction in SA levels - possibly by acting as a signal molecular. Given the perturbation of ammonia and amino acids pools in their RNAi lines - such a mechanism may be relevant to interpreting their findings also - possibly include in Fig 6.

Kim JY, Song JT, Seo HS. Ammonium-mediated reduction in salicylic acid content and recovery of plant growth in Arabidopsis siz1 mutants is modulated by NDR1 and NPR1. Plant Signal Behav. 2021 Sep 2;16(9):1928819. doi: 10.1080/15592324.2021.1928819. Epub 2021 May 14. PMID: 33989128; PMCID: PMC8281091.

Paragraph on sucrose.
I found this to be more speculative and lacking a likely link to the observed phenotypes and could easily be dropped from the discussion.
Possible alternative Pathways for Lignin formation in Grasses.
This interesting possibility is raised by the high levels of 13C9-Tyr incorporated into p-coumarate, sinapate, sinapaldehyde, sinapyl alcohol, and S-units in the Bd C3'H1i lines - and they suggest two possibilities based on work in Arabidopsis.
They should also comment on the recent paper in sorghum where the tyrosine and phenylalanine derived metabolomes were examined including in two bmr mutants affecting CAD and COMT respectively. Are the Tyr and Phe derived metabolites and the pooling seen there similar to what is seen in this study in Brachypodium? - also did the sorghum study see the same lack of Tyr incorporation into the B-ring of isoorientin and isovitexin reported here? (may have to examine their supplemental data).
Simpson Jeffrey P., Olson Jacob, Dilkes Brian, Chapple Clint. (2021) Identification of the Tyrosine- and Phenylalanine-Derived Soluble Metabolomes of Sorghum. Frontiers in Plant Science VOL 12
www.frontiersin.org/article/10.3389/fpls.2021.714164

Materials and Methods:
In this study, the authors employ Brachypodium inbred line Bd21-3. The gene models that they provide appear to be from the reference Bd genome (*Brachypodium distachyon* (Bd21) V3.2 released in 2018). However the genome of the Brachypodium 21-3 line is available on Phytozome since Nov 2019. It is said that the organization of genes is mostly but not always the same in both genomes and this may impact the interpretation of the results. The following quote is from Phytozome "However, Bd21-3 is not a descendent of Bd21 and while they are closely related, they have a considerable number of sequence differences that limit the utility of the Bd21 reference genome for precise studies of Bd21-3 like the identification of specific mutations".
For example in the reference genome PTAL1 (Bradi3G49250) is present on chr 3 as part of a cluster of 4 PAL genes - including Bradi3G49260, Bradi3G49270, and Bradi3G49280. Such tandem repeat clusters of PAL genes have been seen in other grass species including corn. If I use PTAL1 (Bradi3G49250) from Bd21 to search Bd21-3, I find it has 100% identify to BdiBd21-3.3G0653700 which has a similar location relative to the surrounding 3 PAL like genes.
For PAL2 they use Bradi3G49260 which is immediately downstream of Bradi3G49250 and appears to be a tandem duplication (both have 1 intron whereas the other two PALs do not).
They should provide a supplementary folder in which they indicate the models in the Bd21-3 genome (since that is what they used for experimentation) -Or add these model numbers to Supp Table 1.
References: Ok - but see suggestions for some additions in above comments

Supplementary Tables and Figures:
Supp Fig 3: It would have been better if they had provided some evidence that non-targeted PAL family members were not affected in their expression - i.e. that RNAi did not have off-target effects.
Supp Table 2: Part of Table title is missing and Table is spread out over several pages and not readable. It would also be helpful if they added the known or predicted function for the proteins whose abundance is indicated
Supp Fig 8: A side summary bar as in Fig 3 would be helpful to the reader (not essential)

Minor Spelling and grammar errors
L 514 "peroxidases can be phosphorylated" should be "......that can be ....."

Reviewer #2 (Comments for the Author):
The manuscript by Barros et al. details an integrated metabolomic, proteomic and stable isotopic labeling approach to interrogate how RNAi knockdown of key genes in the monolignol biosynthetic pathway globally affected protein and metabolite levels along with metabolic fluxes in stems of the model grass Brachypodium distachyon. Their analyses showed that in stem tissues PTAL1 was the fourth most abundant protein (only RuBisCO subunits and EF1 were found to be more abundant), which suggests the importance of this branch point between primary and secondary metabolism. Moreover, their combined data suggest that important crosstalk between lignin biosynthesis and primary metabolic pathways occurs and may involve abundance of NH3/NH4+ released from tyrosine and phenylalanine by TAL and PAL; the authors hypothesize that perturbation of NH3/NH4+ flux to protein biosynthesis

may underly some of the observed knockdown phenotypes. Also, stable isotope labeling experiments (13C-labeled tyrosine and phenylalanine) uncovered preferential fluxes through different branches of the monolignol biosynthetic pathway; insights were also gleaned into how RNAi knockdown of key biosynthetic genes affected these fluxes.

Taken together, this is a well written and exceptional comprehensive study that provides systems level insights into lignin-related secondary metabolism and how perturbations can feed back causing primary metabolism effects. Moreover, this study provides important insights into how a model monocot grass (Brachypodium) synthesizes lignin differently from dicot species, in the process providing new leads for important follow up studies. While this study is a bit muddied by the use of RNAi constructs which in some instances targeted knockdown of more than just the targeted gene (single gene knockout or knockdown mutants would have provided a cleaner study), by targeting a variety of different genes along the monolignol biosynthetic pathway, an unprecedented view of lignin biosynthetic pathway fluxes and related protein and metabolic perturbations in a grass comes in to focus.

This reviewer has only minor points to make:
1) Page 8, line 213: A reference is listed incorrectly. "[9]" should be replaced with the correct reference.
2) Figure 3: A blue asterisk in the CCoAOMT3 graph is mis-positioned. It should be over the second column and not within the first column.
3) Page 10, lines 289-290: The text states that PAL2 was targeted because it has the highest transcript levels in the stems among all monofunctional PALs, referring to Supplemental Figure S7. However, Supplemental Figure S7 does not show transcript levels for all the PAL isoforms. Please include that data and/or reference Cass et al. (2015) J. Exp. Bot. 66:4317-4335, which shows RNA-seq transcript levels (Brachypodium stem plus leaf sheaths) for the PALs in Table 2.
4) Supplemental Figure S2: Please note in the figure legend what the two different shades of blue delineate.
5) Supplemental Figure S3: Please include statistical analyses showing any significant differences.
6) Supplementary Table 1: The formatting is randomly split between multiple pages in the pdf. Please be sure this is not an issue for the published version.
7) Page 18, line 530: This should read "Supplemental Figure S8" and not "Supplemental Figure S7".

Reviewer #3 (Comments for the Author):
This is an interesting manuscript and I think the present article brings novelty. The authors have undertaken a combined metabolomic and proteomic approach to investigate lignin metabolism in Brachypodium. They use RNAi lines targeting PTAL, PAL2, C4H1, C3'H and HCT1 lines.
It is a huge and comprehensive work. Most data are convincing despite more details on mutant growth phenotypes would have been desirable.
I wonder why authors did not use CRISPR-Cas lines instead of RNAi. Using CRISPR-Cas lines would avoid residual activities of the targeted gene-protein. Is there any reason for choosing this strategy?
The RNAi lines show significant reduction of above ground biomass and reduced lignin content at 30 DAG. This result might be expected if the RNAi lines have some development delay compared with WT as stated page 20, for BdC3'H lines which "show retarded growth phenotype". It would have been interesting to get more information about their development (number of internodes, leaves...). Did all lines flower at the same time ? I guess not, at least for C3'H RNAi lines. Lignin deposition is more impacted by plant development stage and not only by the duration of growth. To bypass this issue, it would have been better to analyze lignin content at dried stage (after 3/4 months for example) to really quantify the impact on lignification and bypass growth delay. If the authors made this analysis and possess data, I would suggest to include them in the manuscript.
Authors investigated putative lignin biosynthesis genes including laccases and peroxidases in the genome of Brachypodium. They found around 90 genes. In supplementary data set 1 and 3, the reference gene identities are for Bd21 genome and not Bd21-3. Please add a column with Bd21-3 gene id (the accession used in this study).
Along the manuscript, genes or proteins are often tagged as "lignin" genes (or proteins) when there are no proof of it to date (and to my knowledge) , for instance (page 19): " the BdPTAL1i lines exhibited the largest lignin reductions concomitant with reduced levels of all three monolignols, and downregulated abundance of 23 lignin peroxidases. »
How do the authors know that these 23 peroxidases are involved in lignification in Brachypodium and not in another metabolism? By the way, it is surprising that the authors do not mention that some laccase genes or proteins were

shown to be involved in lignification in Brachypodium. This is not discussed and completely ignored in the manuscript.
As a general point, literature about Brachypodium research needs to be improved. Previous works on Brachypodium (except those of the authors) are never or rarely cited. For instance, Brachypodium cell wall-related proteomics have been published by other groups.
At last, in contrast to dicots, grasses are known to possess coumarate/ferulate-enriched xylans. This is a grass specificity. The presence of these compounds might also explain for example why all Phe-labeled aldehydes were not reduced by CAD. ALDH might have a role in the production of ferulate/coumarate pools that could be used to the production of ferulated-xylans.
This is consistent with the high abundance of the three soluble proteins (PTAL, C3H and COMT) in grasses. This important aspect is not addressed in the text, and I think it would be worthwhile to do so.

Supp Table 1 please add a column with Brachypodium gene ID to avoid confusion with previously published papers. please add a column with refs if already published for Brachypodium.
FigS5A: why some proteins such as CESA4 are included in the chart with the Y axis legend: lignin protein levels in WT stems.
Supp figS4: are "total aerial biomass" data necessary while stem and leaves biomasses are shown?

TPC2022-RA-00295R1 1st Revision received May 20, 2022

Reviewer comments and **author responses**:

Reviewer #1:

Point 1. The Introduction provides a useful summary of how the main enzymatic steps in the phenylpropanoid pathway have been discovered as well as current differences between dicots and the grasses. These differences provide the rationale for pursuing this work, which involves genetic (RNAi), proteomic, and 13C-isotopic labeling to explore this pathway more comprehensively in Brachypodium.

Results: Fig 1 Good - clear phenotypes showing weaker stems of RNAi lines - but I would have found it helpful to have a small photo (or supplemental figure) showing the internode locations that are collected and the stage of growth for the lignin analysis in Fig 1b. (not an essential addition but helpful to a reader not very familiar with Brachypodium).

> **RESPONSE: A complete description of the samples is now included in the Figure 1 caption. We decided to address this comment in the figure caption rather than using a picture or figure to be also in compliance with the Author Revision Checklist requirements.**

Point 2. It is good that they selected RNAi lines which mostly affected the target gene and not related genes (Supp Fig 3), however I do wonder for the larger gene families with duplicate genes such as the PAL cluster if other members were also affected - if they have data they on the expression of other PALs for example they should share that in the supplemental figure 3 (not essential however). Fig 1B shows clear effects on lignin staining and lignin content and ratios (Fig 1B) This is complemented nicely by the lignin metabolite analysis later (Fig 5). Overall a suitable set of experimental lines to address the effects on metabolic flux.

> **RESPONSE: We measured the expression levels of other members of the target gene families to select the parental lines in the R0 generation. These data have been incorporated as Supplemental Figure S3A, which also includes a statistical analysis as suggested below by Reviewer #2. Please note that among all monofunctional PALs, only *PAL2* has significant transcript levels in the stems. This observation is consistent with previous results reported by Cass et al. in J. Exp. Bot. 66:4317-4335 (2015).**

Point 3. Table 1: OK - as data for other family members are provided in Supp Tables 1 and 2. However see comments in Materials and Methods about providing gene model IDs from the genome of Brachypodium inbred line Bd21-3 and not the reference genome Bd21 since that it is the line they used for measurements and the genome is available. If it makes the table too cluttered then provide these in a supplementary table.

RESPONSE: This comment has been raised also by Reviewer #3. The gene model IDs from the genome of Brachypodium inbred line Bd21-3 are now provided in Supplementary Data Set S1 and S3.

Point 4. Fig 3. Effect of RNAi on enzymes that are different from the targeted one for example - why are so many PALs at lower levels when only one is subject to RNAi? The shaded boxes on the left column are very helpful to get an overview of what the graphs are telling and all family members are included.

RESPONSE: Considering that these effects were more evident in the knockdown lines with severely reduced lignin deposition (*BdPTAL1i* and *BdC4H1i*), we hypothesize that other lignin proteins were subject to feedback downregulation. Our data shows that this occurs widely across the proteome, which argues against off-target transcript silencing, and it could be mediated through the ammonia released during lignin synthesis (Figure 6).

Point 5. Crosstalk between Primary and Secondary Metabolism. I caution against the overinterpretation of metabolic perturbances due to RNAi interference - backups are expected and the ability of plant cells to maintain homeostasis pushed to the limit. However this area of study has value as scientists try to modify lignin content for increased biofuel production and may lead to ways in which the negative growth effects are minimized. In this respect the altered downregulation of enzymes in later genes in the PTAL line is of interest and value - but may also reflect regulation at the transcriptional level.

RESPONSE: We edited this paragraph to address this point and the comments raised below by Reviewer #3 on the regulation of the lignin polymerization step via laccases and peroxidases.

Point 6. Link to SA metabolism. They might include in their interpretation the recent observations by Kim et al. 2021 that exogenous ammonia can cause a reduction in SA levels - possibly by acting as a signal molecule. Given the perturbation of ammonia and amino acids pools in their RNAi lines - such a mechanism may be relevant to interpreting their findings also - possibly include in Fig 6. Kim JY, Song JT, Seo HS. Ammonium-mediated reduction in salicylic acid content and recovery of plant growth in Arabidopsis siz1 mutants is modulated by NDR1 and NPR1. Plant Signal Behav. 2021 Sep 2,16(9):1928819. doi: 10.1080/15592324.2021.1928819. Epub 2021 May 14. PMID: 33989128, PMCID: PMC8281091.

RESPONSE: Thank you, this is a really interesting paper. It will be informative to perform similar evaluations to study the regulation of growth and SA content by ammonium using other dwarf mutants. This reference is now incorporated in the discussion. SA was not included in the model Figure because our results indicate that SA is not the cause of the growth phenotype observed in the Brachypodium RNAi lines.

Point 7. In this study, the authors employ Brachypodium inbred line Bd21-3. The gene models that they provide appear to be from the reference Bd genome (*Brachypodium distachyon* (Bd21) V3.2 released in 2018). However the genome of the Brachypodium 21-3 line is available on Phytozome since Nov 2019. It is said that the organization of genes is mostly but not always the same in both genomes and this may impact the interpretation of the results. The following quote is from Phytozome "However, Bd21-3 is not a descendent of Bd21 and while they are closely related, they have a considerable number of sequence differences that limit the utility of the Bd21 reference genome for precise studies of Bd21-3 like the identification of specific mutations". For example in the reference genome PTAL1 (Bradi3G49250) is present on chr 3 as part of a cluster of 4 PAL genes - including Bradi3G49260, Bradi3G49270, and Bradi3G49280. Such tandem repeat clusters of PAL genes have been seen in other grass species including corn. If I use PTAL1 (Bradi3G49250) from Bd21 to search Bd21-3, I find it has 100% identify to BdiBd21-3.3G0653700 which has a similar location relative to the surrounding 3 PAL like genes. For PAL2 they use Bradi3G49260 which is immediately downstream of Bradi3G49250 and appears to be a tandem duplication (both have 1 intron whereas the other two PALs do not).

They should provide a supplementary folder in which they indicate the models in the Bd21-3 genome (since that is what they used for experimentation) -Or add these model numbers to Supp Table 1.

RESPONSE: Thank you for sharing this observation. Recently, we also noticed similar monofunctional PAL tandem duplicates in poplar (Potri010G224100; Potri010G224200). The gene model IDs from Brachypodium Bd21-3 are now provided as Supplemental Data Set S1 and S3. We also included a note with this information in the section "Accession numbers".

Point 8. Supp Fig 3: It would have been better if they had provided some evidence that non-targeted PAL family members were not affected in their expression - i.e. that RNAi did not have off-target effects.

RESPONSE: Supplemental Figure S3A now provides the transcript levels of other members of the target gene families used for the selection of the parental lines. Note that among all ammonia-lyases, only *PAL2* and *PTAL1* have notable expression levels in stems (Cass et al., 2015).

Point 9. Supp Table 2: Part of Table title is missing and Table is spread out over several pages and not readable. It would also be helpful if they added the known or predicted function for the proteins whose abundance is indicated

RESPONSE: This is a comment also made by Reviewer #2. We will try to make the Supplementary Data Sets available for the reviewers as single Excel files (.xls).

Reviewer #2:

Point 1. (Minor comments.)

RESPONSE: Thank you for the positive review. All the comments above have been addressed in the revised version of the manuscript.

Reviewer #3:

Point 1. I wonder why authors did not use CRISPR-Cas lines instead of RNAi. Using CRISPR-Cas lines would avoid residual activities of the targeted gene-protein. Is there any reason for choosing this strategy?

RESPONSE: When we started this project, we were not set up to do genome editing in Brachypodium. However, the foremost reason why we decided to apply an RNAi silencing approach instead of a loss-of-function strategy was because total knock-out of some lignin pathway genes — particularly those blocking the shikimate esters pathway — are prone to cause highly dwarf or lethal phenotypes, and RNAi silencing is a suitable approach when studying the effect of essential genes.

Point 2. The RNAi lines show significant reduction of above ground biomass and reduced lignin content at 30 DAG. This result might be expected if the RNAi lines have some development delay compared with WT as stated page 20, for BdC3'H lines which "show retarded growth phenotype". It would have been interesting to get more information about their development (number of internodes, leaves...). Did all lines flower at the same time? I guess not, at least for C3'H RNAi lines. Lignin deposition is more impacted by plant development stage and not only by the duration of growth. To bypass this issue, it would have been better to analyze lignin content at dried stage (after 3/4 months for example) to really quantify the impact on lignification and bypass growth delay. If the authors made this analysis and possess data, I would suggest to include them in the manuscript.

RESPONSE: The length and number of internodes in the RNAi lines is now provided as Supplementary Figure S4A. All RNAi lines were harvested when the first inflorescence was emerging, and we did not notice flowering differences among genotypes. The quoted sentence from the paragraph on sucrose was removed, as suggested above by Reviewer #1, and we actually meant 'reduced' instead of 'retarded' growth.

We agree and understand the last concern in this comment; it is difficult to disentangle lignin deposition and plant development. Plants were harvested for analyses before senescence to be able to perform the proteome analyses; therefore, we do not have lignin data from mature dried plants. Our study would clearly benefit from comprehensive time-course studies, and this is something we aim to address in future work, as is now acknowledged in the last sentence of the Discussion.

Point 3. Along the manuscript, genes or proteins are often tagged as "lignin" genes (or proteins) when there are no proof of it to date (and to my knowledge) , for instance (page 19): " the BdPTAL1i lines exhibited the largest lignin reductions concomitant with reduced levels of all three monolignols, and downregulated abundance of 23 lignin peroxidases." How do the authors know that these 23 peroxidases are involved in lignification in Brachypodium and not in another metabolism? By the way, it is surprising that the authors do not mention that some laccase genes or

proteins were shown to be involved in lignification in Brachypodium. This is not discussed and completely ignored in the manuscript.

RESPONSE: To address the first point, we have incorporated the following sentence at the end of the paragraph describing our genomic approach to identify the enzymes involved lignin formation in Brachypodium stems: "This approach allowed us to identify the closest orthologs to annotated lignin biosynthetic enzymes in Arabidopsis. However, some of these genes still remain to be functionally characterized." We also referenced in Supplemental Data Set S1 the identification of the Arabidopsis monolignol biosynthesis gene families from TAIR: https://arabidopsis.org/browse/genefamily/Raes.jsp

Source: Raes, J., Rohde, A., Christensen, J., Van de Peer, Y., Boerjan, W. Genome-wide characterization of the lignification toolbox in Arabidopsis thaliana. Plant Phys 133: 1051-71.

We changed the sentence quoted above to: "... the BdPTAL1i lines exhibited the largest lignin reductions concomitant with reduced levels of all three monolignols, several lignans, and downregulated abundance of 23 proteins annotated in the KEGG database as lignin peroxidases. Although laccases are not annotated in KEGG as lignin oxidizing enzymes, LAC7 was found to be the most significantly downregulated lignin protein in the BdPTAL1i lines (Figure 3)". We also incorporated some additional discussion on lignin laccases and their regulation at the end of this paragraph.

Point 4. As a general point, literature about Brachypodium research needs to be improved. Previous works on Brachypodium (except those of the authors) are never or rarely cited. For instance, Brachypodium cell wall-related proteomics have been published by other groups.

RESPONSE: We have incorporated some references and analyzed the cell-wall proteomics data reported by Douché et al., 2021 in Supplemental Figure S5B. Thank you.

Point 5. At last, in contrast to dicots, grasses are known to possess coumarate/ferulate-enriched xylans. This is a grass specificity. The presence of these compounds might also explain for example why all Phe-labeled aldehydes were not reduced by CAD. ALDH might have a role in the production of ferulate/coumarate pools that could be used to the production of ferulated-xylans. This is consistent with the high abundance of the three soluble proteins (PTAL, C3H and COMT) in grasses. This important aspect is not addressed in the text, and I think it would be worthwhile to do so.

RESPONSE: Thank you again for this suggestion. We were wondering for a while what could be a putative metabolic sink for this flux in the pathway. We incorporated a paragraph in the Discussion and some references to address this possibility.

Point 6. Supp figS4: are "total aerial biomass" data necessary while stem and leaves biomasses are shown?

RESPONSE: We kept these data because we believe that it is informative to see the total amount of aerial biomass per plant at first sight.

**TPC2022-RA-00295R1 2nd Editorial decision – *acceptance pending***	**May 23, 2022**

We are pleased to inform you that your paper entitled "Proteomic and metabolic disturbances in lignin-modified Brachypodium distachyon" has been accepted for publication in The Plant Cell, pending a final minor editorial review by journal staff. At this stage, your manuscript will be evaluated by a Science Editor with respect to its presentation of scientific content, compliance with journal policies, and presentation for a broad readership.

**Final acceptance from Science Editor**	**June 2, 2022**
